# Variation and covariation of external shape and cross‐sectional geometry in the human metacarpus

**DOI:** 10.1002/ajpa.24866

**Published:** 2023-11-06

**Authors:** Samuel B. Tanner, Ameline Bardo, Thomas W. Davies, Christopher J. Dunmore, Richard E. Johnston, Nicholas J. Owen, Tracy L. Kivell, Matthew M. Skinner

**Affiliations:** ^1^ School of Anthropology and Conservation University of Kent Canterbury UK; ^2^ UMR 7194 ‐ Histoire Naturelle de l'Homme Préhistorique (HNHP) CNRS‐Muséum National d'Histoire Naturelle Paris France; ^3^ Department of Human Origins Max Planck Institute for Evolutionary Anthropology Leipzig Germany; ^4^ Advanced Imaging of Materials (AIM) Facility, Faculty of Science and Engineering, Bay Campus Swansea University Swansea UK; ^5^ Applied Sports Technology Exercise and Medicine Research Centre (A‐STEM), School of Engineering and Applied Sciences, Bay Campus Swansea University Swansea UK

**Keywords:** cortical bone strength, geometric morphometrics, Mary Rose, metacarpals, robusticity

## Abstract

**Objectives:**

Analyses of external bone shape using geometric morphometrics (GM) and cross‐sectional geometry (CSG) are frequently employed to investigate bone structural variation and reconstruct activity in the past. However, the association between these methods has not been thoroughly investigated. Here, we analyze whole bone shape and CSG variation of metacarpals 1–5 and test covariation between them.

**Materials and Methods:**

We analyzed external metacarpal shape using GM and CSG of the diaphysis at three locations in metacarpals 1–5. The study sample includes three modern human groups: crew from the shipwrecked Mary Rose (*n* = 35 metacarpals), a Pre‐industrial group (*n* = 50), and a Post‐industrial group (*n* = 31). We tested group differences in metacarpal shape and CSG, as well as correlations between these two aspects of metacarpal bone structure.

**Results:**

GM analysis demonstrated metacarpus external shape variation is predominately related to changes in diaphyseal width and articular surface size. Differences in external shape were found between the non‐pollical metacarpals of the Mary Rose and Pre‐industrial groups and between the third metacarpals of the Pre‐ and Post‐industrial groups. CSG results suggest the Mary Rose and Post‐industrial groups have stronger metacarpals than the Pre‐industrial group. Correlating CSG and external shape showed significant relationships between increasing external robusticity and biomechanical strength across non‐pollical metacarpals (*r*: 0.815–0.535; *p* ≤ 0.05).

**Discussion:**

Differences in metacarpal cortical structure and external shape between human groups suggest differences in the type and frequency of manual activities. Combining these results with studies of entheses and kinematics of the hand will improve reconstructions of manual behavior in the past.

## 1 INTRODUCTION

The ability to manufacture and use tools has played a key role in human evolutionary history (e.g., Ambrose, 2001; Panger et al., 2002; Shea, 2017) and as such there is a wealth of research that has focused on hand morphology and use in humans, and nonhuman primates, to infer the manipulative capabilities of extinct hominins (e.g., Bardo et al., 2020; Byrne et al., 2001; Dunmore et al., 2020; Karakostis et al., 2018; Marzke, 1997; Napier, 1956). This research has been devoted to understanding when, where, and how the human hand developed its precision dexterity and the functional changes that enabled the hand to withstand the biomechanical forces associated with making and using stone tools (e.g., Dunmore et al., 2020; Galletta et al., 2019; Karakostis et al., 2021; Marzke, 2013; Prang et al., 2021; Williams‐Hatala et al., 2018). Experimental studies of grip types, muscle activity and external loads experienced by the hand during tool manufacture or use can also provide crucial information about the biomechanical environment of different hand bones and joints (e.g., Key et al., 2020; Key & Dunmore, 2015; Marzke & Shackley, 1986; Rolian et al., 2011; Williams‐Hatala et al., 2021).

Much research on the hand has focused on the modern human thumb and/or the index and middle fingers, due to the important role they play in numerous forceful pad‐to‐pad precision grips that are thought to be necessary to create early technologies (e.g., Hamrick et al., 1998; Marzke et al., 1999; Niewoehner et al., 2003; Rolian et al., 2011; Williams et al., 2012; see review in Kivell et al., 2023). Furthermore, the relatively large musculature and enhanced opposability of the human thumb compared to other primates is argued to be a defining human adaptation (Key & Dunmore, 2015; Marzke, 1997; Marzke et al., 1999; Niewoehner et al., 2003; Rolian et al., 2011; Shrewsbury et al., 2003; Williams et al., 2012). As a result of this focus on the thumb, there has been considerably less research into the non‐pollical rays of the hand. This paper builds on previous research through a comprehensive examination of external and internal bony structures across the entire metacarpus of three distinct human samples.

### 1.1 Measuring metacarpal shape and cortical bone structure

Several aspects of bone structure can be examined in an attempt to reconstruct behavior including the architecture of trabecular bone (e.g., Kivell, 2016; Tsegai et al., 2013), the distribution of cortical bone in the diaphysis (e.g., Lieberman et al., 2004; Ruff & Hayes, 1983; Stock & Pfeiffer, 2001), the size and morphology of entheses (e.g., Karakostis et al., 2017, 2018, 2019, 2021; Lieverse et al., 2013), and external bone shape (e.g., Bardo et al., 2020; Niewoehner, 2005). The link between bone structure and behavior can be investigated via a process called bone functional adaptation in which the shape and structure of bone actively (re‐)model during life in response to biomechanical loading through the activity of osteoclasts and osteoblasts (Currey, 2002; Eriksen, 1986, 2010; Goodship et al., 1979; Ruff et al., 2006). It is possible to reconstruct aspects of behavior from bone in individuals with no associated behavioral histories by comparing them to those with known functional/behavioral repertoires and evaluating any similarities or differences (Carter & Beaupré, 2001; Currey, 2002; Martin et al., 1998; Ruff et al., 2006; Su & Carlson, 2017). For the purpose of this study, it is worth noting, however, that unlike cortical and trabecular bone (Eriksen, 1986, 2010), aspects of external bone shape, particularly articular surfaces, have been shown to be less plastic throughout life despite changes in loading, age or body mass (Lieberman et al., 2001; Ruff et al., 1991).

The thickness and distribution of diaphyseal cortical bone around the medullary cavity is proportional to a bone's resistance to force in different directions (Ruff & Hayes, 1983). This cross‐sectional geometry (CSG) can be measured at different points along the diaphysis to approximate the biomechanical strength of the bone in response to different types of loading, and, in turn, can be used to make inferences about behavior (Bauchau & Craig, 2009; Griffin & Richmond, 2005; O'Neill & Ruff, 2004; Salathe et al., 1989). CSG analysis has been applied to various long bones across the skeleton to compare bone strength and infer differences in activity levels and behavior between human populations (e.g., Hagihara, 2021; Larsen & Ruff, 1991; Lazenby, 1998; Ruff, 1992; Ruff & Hayes, 1983; Stock & Pfeiffer, 2001), extant primates (e.g., Demes & Jungers, 1989; Marchi, 2005; Ruff, 1987; Ruff & Runestad, 1992), and fossil hominins (e.g., Kubicka et al., 2022; Trinkaus & Ruff, 2012; Zipfel et al., 2020).

While some earlier studies of internal bone architecture in response to changes and differences in behavior have found varying levels of support for a link between internal bone structure and behavior (e.g., Barak et al., 2011; Fajardo et al., 2007; Pontzer et al., 2006; Shaw & Ryan, 2012), there is a growing body of evidence using recent methods for a strong link between internal bone structure and behavioral variation (see Dunmore et al., 2020; Kivell, 2016; Patel et al., 2020; Syeda et al., 2023; Tsegai et al., 2017). However, it must be noted that bone form is not only effected by behavior but can also be influenced by other factors such as age (Maggio et al., 1997; Trotter et al., 1960; Villotte et al., 2022), sex (Jepsen et al., 2015; Trotter et al., 1960), and genetics (Judex et al., 2004; Wallace et al., 2015), which may impact an investigation such as this incorporating samples from multiple distinct populations.

In contrast to measures of internal bone, there has been considerably more debate over behavioral reconstructions based on external bone morphology (e.g., Begun & Kivell, 2011; Lewin, 1983; Lovejoy, 2009; Richmond & Strait, 2000; Stern, 1975; Susman, 2004; Ward, 2002, 2013; Wood & Harrison, 2011). Regions of external bone shape such as articular facets are considered to be more constrained by genetics and function than aspects of diaphyseal structure, and therefore may be less able to adapt to, and thus record, habitual loads in life compared to aspects of internal bone structure (Currey, 2002; Lieberman et al., 2001; Ruff et al., 1991; Ruff et al., 2006; Ruff & Runestad, 1992).

Traditionally, external metacarpal shape has been assessed through linear measurements such as the maximum length, articular length, base dorsopalmar height, and base mediolateral width (see Bush et al., 1982; Morrish & Hlusko, 2014; North & Rutledge, 1983; Smith, 2000), and in some cases these measurements have been used to estimate stature and sex across a range of human populations (e.g., Alabi et al., 2020; DeSilva et al., 2014; Khanpetch et al., 2012; Kimura, 1992; Meadows, 1990; Musgrave & Harneja, 1978). More recently, researchers have utilized a 3D geometric morphometric (GM) approach to comparatively analyze extant hominid metacarpal morphology and make interpretations of function in extinct hominin specimens. For example, studies have focused on the shaft of the first metacarpal (Bowland et al., 2021; Morley et al., 2020) and its distal or proximal articulations (Bardo et al., 2020; Galletta et al., 2019; Marchi et al., 2017; Niewoehner, 2005). To date, only a few studies have considered the shape of the third metacarpal and its articulations (Rein, 2019; Rein & Harvati, 2013), and none have been conducted on the second, fourth and fifth metacarpals of which we are aware.

Allometry can also be an important factor in interpreting variation in external bone shape. Previous interspecific metacarpal studies of modern humans, great apes, and fossil hominins did not find a significant allometric impact on aspects of metacarpal shape (Bardo et al., 2020; Bowland et al., 2021; Galletta et al., 2019; Morley et al., 2020; Niewoehner, 2005), but a wider primate sample including modern humans, great apes, fossil hominins, cercopithecoids, and platyrrhine metacarpals displayed a significant relationship between size and shape (Rein & Harvati, 2013); however these studies are not entirely comparable to this current investigation as we only deal with intraspecific variation. In this study, we use whole surface GM to capture the shape of both the proximal and distal articular surfaces and the diaphysis of MC1‐5 and combine this with analyses of CSG. We test for allometry on metacarpal shape with the expectation that results will be consistent with previous GM studies that have used solely hominoid samples (Bardo et al., 2020; Bowland et al., 2021; Galletta et al., 2019; Morley et al., 2020; Niewoehner, 2005) and shown size to be a minor contributor to metacarpal shape variation.

Internal and external approaches to studying bone functional adaptation are common and provide us with valuable insights into behavior (e.g., Bardo et al., 2020; Bird et al., 2021; Doershuk et al., 2019; Dunmore et al., 2019; Dunmore et al., 2020; Gross et al., 2014; Karakostis et al., 2017; Kivell, 2016; Mulder, 2020; Plochocki et al., 2006; Profico, Bondioli, et al., 2021; Profico, Zeppilli, et al., 2021; Saers et al., 2019; Zhao et al., 2021). Many of these investigations have been framed within an evolutionary context and hence used broad samples of extant hominids to reconstruct elements of behavior in extinct hominins. However, investigations that have relied solely on human samples to identify potential differences based on occupation and inferred hand use suggest that aspects of external bone shape such as entheses (Karakostis et al., 2017; Karakostis & Hotz, 2022) can also distinguish between individuals in different behavioral categories (occupations with high intensity manual loading vs. lower intensity/mechanized jobs) in the same manner that the study of internal bone routinely offers (e.g., Doershuk et al., 2019; Profico, Zeppilli, et al., 2021; Saers et al., 2019). Nonetheless, it is rare for both internal and external aspects of bone structure to be analyzed in conjunction (see Kubicka & Myszka, 2020).

### 1.2 Objectives and hypotheses

The aim of this study is to investigate variation and covariation in external shape (using 3D GM) and diaphyseal structure (using CSG) of the metacarpus within and between three samples of recent *Homo sapiens*. These samples include Pre‐industrial and Post‐industrial individuals, as well as, a unique sample of soldiers and sailors from a medieval warship, the Mary Rose (Stirland, 2005), which have been reported to have robust bones with several indicators of high activity levels and repetitive, high intensity loading in the arm and across the skeleton, including enlarged shoulder dimensions, the high frequency of an *os acromiale*, and enlargements to the greater trochanter of the femur (Stirland, 2005, 2012). The objectives of the study are to: (1) examine the major patterns of external shape variation in each metacarpal and whether these are consistent among our human groups; (2) test for allometric trends in external metacarpal shape; (3) test for differences in diaphyseal CSG both across the metacarpus and between human groups; and (4) test for correlations between external metacarpal shape and cross‐sectional properties of the diaphysis.

Regarding the first objective, we test the null hypothesis that all three human groups will not be significantly different in terms of their whole bone shape. For our second objective, we hypothesize that there will be no allometric signal in external metacarpal shape in this intraspecific study given the results of previous interspecific studies of hominoid metacarpals (Bardo et al., 2020; Bowland et al., 2021; Galletta et al., 2019; Morley et al., 2020; Niewoehner, 2005). Based on bone functional adaptation, we hypothesize for our third objective that the Mary Rose and Pre‐Industrial populations, which we assume engaged in intense manual daily activities, will have greater cross‐sectional properties than our Post‐Industrial sample, but that the patterns of cross‐sectional properties across the palm will be the same for all three groups (i.e., MC1 largest, followed by MC2 + 3, followed by the MC4 + 5). For our fourth objective, we hypothesize that the main components of shape variation, quantified through principal component analysis of the GM shape data for each metacarpal, will be positively correlated with CSG properties due to a relationship between these highlighted aspects of shape variation and activity.

## 2 MATERIALS AND METHODS

### 2.1 Sample

The composition of the study sample is provided in Table 1 (also see Table S1). The sample comprises 116 metacarpals (a mix of left and right sides) from three spatiotemporally diverse human groups. The metacarpals of the Mary Rose sample (*n* = 35; 10 from two complete hands; 25 unassociated) are likely all from males in the 16th century (England), with ~85% of all crew members falling between the ages of 19–29 (Stirland, 1985; Stirland & Waldron, 1997). Historical evidence indicates these individuals were either sailors or soldiers and thus, are likely to have experienced habitually high manual loads (Stirland, 2005, 2012). This sample has marked external indicators of skeletal robusticity, including enlarged left shoulder dimensions and enhanced muscle attachment sites for the gluteal and thigh muscles (Stirland, 2005, 2012).

**TABLE 1:** Study sample breakdown by human group and metacarpal.

Group	Metacarpal				
	MC1	MC2	MC3	MC4	MC5
Mary Rose	5	7	10	7	6
Post‐Industrial	6	7	7	6	5
Pre‐Industrial	9	11	9	10	11
**Total**	**20**	**25**	**26**	**23**	**22**

The Pre‐industrial group is composed of six different samples from Australia, Canada, the Chatham Islands, Egypt, Greenland, and Tierra del Fuego (*n* = 50; 25 from five complete hands, 25 unassociated) and ranges from 5th century to late 19th century time periods (Table 1). All these individuals are assumed to come from foraging groups that likely utilized subsistence activities that generated high loads in the dominant and non‐dominant hand (Hayden, 1981; Key, 2016; Key et al., 2017; Key & Dunmore, 2015; Kitanishi, 1995; Rolian et al., 2011; Stock & Pfeiffer, 2004; Williams‐Hatala et al., 2018, 2021) and therefore we expect individuals from this group to possess robust metacarpals.

The Post‐industrial group contains individuals from an 18th–19th Century German cemetery in Göttingen (*n* = 31; 5 from one complete hand, 26 unassociated). While no specific information about activity levels or occupation is associated with these individuals, we assume that physical manual activities were less intense due in part to a wider adoption of tools and machinery, combined with higher rates of sedentariness in post‐industrial populations (Chirchir et al., 2017; Malina & Little, 2008; Trinkaus, 2016). All specimens used in this study were examined externally and internally for evidence of growth plates and all were considered skeletally mature. The impact of sex and age in this dataset is unable to be tested as there is limited data available on the sex of the Pre‐ and Post‐industrial samples, and the ages of all sampled individuals.

### 2.2 Microtomography

Microtomographic scans of the samples were obtained using either a BIR ACTIS 225/300 high‐resolution microCT scanner (130 kV and 100 μA using a 0.25 mm brass filter), a SkyScan 1173 (100–130 kV and 90–130 μA), or a Nikon XTH225 MicroCT Scanner (85–160 kV and 140–190 μA using a 0.25 mm copper or a 0.5 mm aluminum filter) at an average isotopic voxel size of 39.6 μm (range of 25.1–55.5 μm). Scans were reconstructed as 16‐bit TIFF stacks.

### 2.3 Geometric morphometric analysis

**FIGURE 1:**
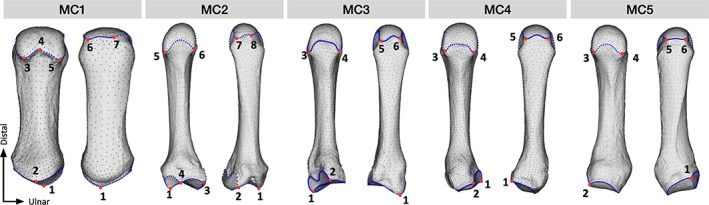
Landmarking protocol for each metacarpal. A set of left metacarpals is shown, depicting placed landmarks on metacarpals 1–5. Red = fixed landmarks, Blue = curve landmarks, Black = surface semi‐landmarks. From left to right, each metacarpal example shows the palmar and dorsal views.

Image stacks were first processed in Avizo 6.3 (Thermofisher Scientific, USA) where the volumetric metacarpal images were meshed as surfaces and saved as .ply files, followed by the removal of the internal structure within MeshLab (Cignoni et al., 2008). Finally, the resulting surfaces were cleaned in Geomagic (3D Systems, Inc., USA), and as we used a mix of right and left bones, we mirrored metacarpals where necessary to match template specimens in order to ensure homologous shape comparisons. Fixed (anatomical) landmarks and the sliding semi‐landmarks on curves were placed manually on the articular surfaces of each metacarpal surface model in Avizo 6.3 using landmarking protocols outlined in Table 2 and shown in Figure 1 (see Figures S1–S5 for more detail). Five template specimens of the metacarpus were produced, with the breakdown of landmark numbers for each metacarpal as follows: MC1–1002 total landmarks (7 fixed, 128 on curves, 876 on surface), MC2–1292 total (8 fixed, 144 on curves, 1140 on surface), MC3–1670 total (6 fixed, 260 on curves, 1404 on surface), MC4–1790 total (6 fixed, 176 on curves, 1608 on surface), MC5–1298 total (6 fixed, 176 on curves, 1116 on surface). Surface sliding semi‐landmarks were placed at high spatial density to allow the overall shape to be quantified in as much detail as possible. Significant intra‐ and inter‐observer error in landmark placement was tested using the Procrustes distance between repeats (Figure S6 and Table S3). For these tests, five specimens were repeatedly landmarked five times by one author (ST) and once by another researcher, with 1 day between repeats.

**TABLE 2:** Landmark definitions for GM analysis of metacarpal shape.

		Landmark number/definition and path of curve landmarks
MC1	Proximal	Apex of trapezium articulation on the dorsal side Apex of trapezium articulation on the palmar side Path of Curves: 1–2–1 (trapezium articular surface)
	Distal	3 Ulnar, palmar corner of distal articular surface 4 Middle of the palmar surface of the distal articular surface, at the apex of the invagination 5 Radial, palmar corner of distal articular surface 6 Radial, dorsal corner of distal articular surface 7 Ulnar, dorsal corner of distal articular surface Path of Curves: 3–4–5–6–7–3 (distal articular surface)
MC2	Proximal	Radial, dorsal corner of the trapezoid articular surface that connects to the trapezium articular surface Ulnar, dorsal corner of the trapezoid articular surface that connects to the MC3 articular surface Ulnar, palmar corner of the trapezoid articular surface that connects to the MC3 articular surface Radial, palmar corner of trapezoid articular surface, where it meets the facet for the trapezium Path of Curves: 1–2–3–4–1 (trapezoid facet), 2–3 (MC3 facet), 4–1 (trapezium articular surface)
	Distal	5 Radial palmar corner of distal articular surface 6 Ulnar palmar corner of distal articular surface 7 Ulnar dorsal corner of distal articular surface 8 Radial dorsal corner of distal articular surface Path of Curves: 5–6–7–8–5 (distal articular surface)
MC3	Proximal	Radial, dorsal corner where the capitate and MC2 articular surfaces meet Radial, palmar corner where the capitate and MC2 articular surfaces meet Path of Curves: 1–2–1 (capitate articular surface), 1–2 (MC2 facet)
	Distal	3 Radial, palmar corner of distal articular surface 4 Ulnar palmar corner of distal articular surface 5 Ulnar dorsal corner of distal articular surface 6 Radial, dorsal corner of distal articular surface Path of Curves: 3–4–5–6–3 (distal articular surface)
MC4	Proximal	Ulnar, dorsal corner where the hamate and MC5 articular surface meet Ulnar, palmar corner where the hamate and MC5 articular surfaces meet Path of Curves: 1–2–1 (hamate articular surface), 2–1 (MC5 facet)
	Distal	3 Radial, palmar corner of distal articular surface 4 Ulnar palmar corner of distal articular surface 5 Ulnar dorsal corner of distal articular surface 6 Radial, dorsal corner of distal articular surface Path of Curves:3–4–5–6–3 (distal articular surface)
MC5	Proximal	Radial, dorsal corner where the MC4 and hamate facets meet Radial, palmar corner where the MC4 and hamate facets meet Curve paths: 1–2–1 (hamate articular surface), 1–2 (MC4 facet)
	Distal	3 Radial palmar corner of distal articular surface 4 Ulnar palmar corner of distal articular surface 5 Ulnar dorsal corner of distal articular surface 6 Radial dorsal corner of distal articular surface Path of Curves: 3–4–5–6–3 (distal articular surface)

*Note*: Numbers are for the fixed landmarks around the proximal and distal articular surfaces and the path of all curve landmarks around articular surfaces are also defined. Note that some articulations on the base of MC3 and MC4 (where they articulate with each other) were not landmarked due to high intraspecific variability in form.

Geometrically homologous semi‐landmarks on curves were derived in R using the Morpho (v2.9; Schlager, 2017) and Geomorph packages (v4.0.1; Adams et al., 2022; Baken et al., 2021; Collyer & Adams, 2018, 2021). A smooth curve was fit through the curve landmark sets using a cubic *spline* function, and then a fixed number of equally spaced semi‐landmarks were placed along each curve section using the *digit.curves* function. Surface semi‐landmarks were then projected on to each specimen and relaxed against the template specimen using the *placePatch* and *relaxLM* functions. Curve semi‐landmarks were then allowed to slide along tangents to the curves, and surface semi‐landmarks along tangent planes to the surface using the *slider3D* function, to minimize the bending energy of the thin‐plate spline interpolation function between each specimen and the Procrustes average for the sample (Gunz & Mitteroecker, 2013). The sliding procedure was performed twice, with the semi‐landmarks being projected back onto the curves/surface after each round of sliding using the *project_to_curve* function from the princurve package (Hastie et al., 2022), after which the semi‐landmarks were considered to be geometrically homologous and were converted into shape coordinates using generalized least squares Procrustes superimposition (using the *procSym* function) which standardizes position, scale and orientation of landmarks for each specimen in the sample (Rohlf & Slice, 1990).

To assess variation in external shape, principal component analysis (PCA) was conducted on the Procrustes coordinates of each ray separately. Pairwise group differences in external shape were tested for using permutational Procrustes ANOVAs with Bonferroni corrections, where each metacarpal was considered separately. To test for allometric effects on external shape, permutational Procrustes ANOVAs were conducted between shape coordinates and two measurements of size: maximum metacarpal length and centroid size (i.e., the square root of the sum of squared distances between each landmark and the centroid; Klingenberg, 2014). All permutational Procrustes ANOVA tests were conducted using the *procD.lm* function in the Geomorph R package with 9999 iterations. For an assessment of variation in whole bone shape within and between groups, using the Procrustes distances between individuals, see section 10 in Data S1.

Surface models were created to visualize shape deformations associated with the extremes of the PC1 and PC2 axes, by warping the template surface of each metacarpal to the target shape (i.e., coordinates computed for each extreme, +/− 1.5 standard deviations of the relevant PC axis) using the Morpho package in R. Group mean shapes were also visualized by warping the template specimen surfaces to group mean coordinates. Heat maps of distances between group mean models were constructed within MeshLab using the sampling filter “distance from reference mesh” for each metacarpal comparison.

### 2.4 CSG analysis

**FIGURE 2:**
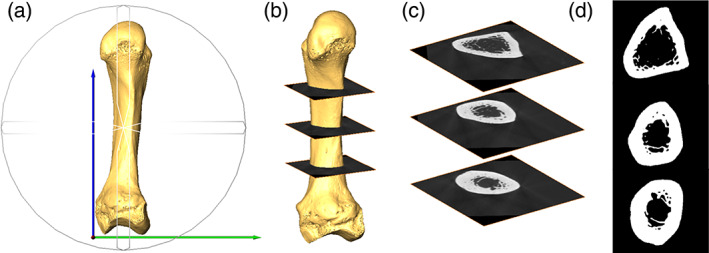
Methodological steps for metacarpal cross‐sectional geometry analyses. (a) Standard anatomical orientation of a second metacarpal, as an example, with the long axis of bone parallel to the *y*‐axis; (b) image highlighting the extraction of the cross‐sections at 33%, 50%, and 66% of the total bone length; (c) an example of the sampled cross‐sections; and (d) binarized cross‐sections.

Volumetric metacarpal models were reoriented in Avizo 6.3 into a standard anatomical position as previously described by Marchi (2005); Figure 2), and three cross‐sections at 33%, 50% and 66% of the metacarpal length were extracted from each reconstructed microCT volume (Figure 2). Three measures of CSG were determined using the BoneJ plugin (Domander et al., 2021) in ImageJ (Schindelin et al., 2012): cross‐sectional area (*CSA*), the maximum second moment of area (*I*_max_) and the minimum second moment of area (*I*_min_). The reorientation process has been shown to significantly affect the results of CSG analysis (Ruff & Hayes, 1983) and thus we tested the effect of 10 reorientations of a single first metacarpal (MC1) on CSG values. The results demonstrated that the three CSG variables (*CSA*, *I*_max_, *I*_min_) varied <0.31% between orientations and so the potential effect of reorientation error on the results was considered negligible (Table S2).

In this study, *CSA* is used as it is proportional to the relative resistance of a diaphysis to axial compression and tension. By combining the maximum and minimum second moments of area (*I*_max_ + *I*_min_) we calculated the polar moment of inertia (*J*), which has been shown to be proportional to the strength of a diaphysis to torsional deformation (Griffin & Richmond, 2005; Lieberman et al., 2004). As some of the CSG data was not normally distributed (Table S4), group and metacarpal differences in *J* and *CSA* were tested by using pairwise Wilcoxon rank sum tests with post hoc Bonferroni corrections. All values of *J* and *CSA* presented in this paper were not standardized by length in order to preserve the absolute differences across the metacarpus. However, we have included test results using length standardized data in section 9 of Data S1; with the standardization of CSG values providing very similar results to the non‐standardized data.

### 2.5 Analyses of external and internal shape covariation

The Pearson's correlation coefficient of CSG variables, *J* and *CSA*, with the scores of the first two principal components from the PCA of Procrustes coordinates, were calculated to test for co‐variation between CSG and external metacarpal shape. This was repeated for both CSG variables at each of the diaphyseal cross‐sections (33%, 50% and 66%) for each metacarpal, with Bonferroni corrections for multiple testing. Principal components of shape variation beyond the first and second are not discussed in this paper as there were very few significant correlations noted between PCs3‐5 with J and CSA.

## 3 RESULTS

### 3.1 Geometric morphometric investigation of metacarpal shape

**FIGURE 3:**
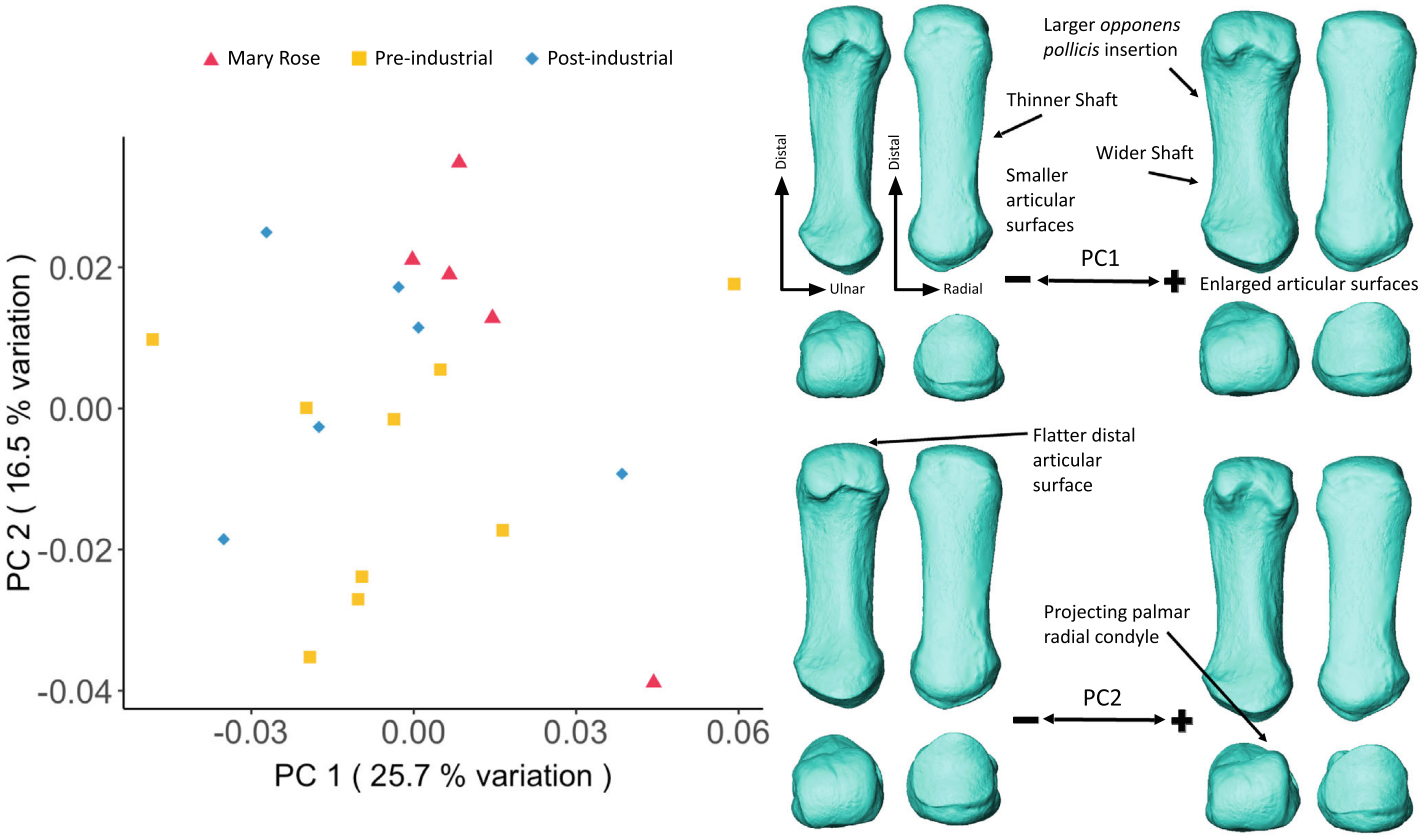
First metacarpal external shape variation. On the left, PCA plot of PC1 and PC2, showing variation in the first metacarpal shape and, on the right, surface warps depicting the morphological changes captured by each extreme of the principal component axes, with shape changes labeled (top images show palmar (left) and dorsal views (right); bottom images show distal (left) and proximal (right) views).

The PCA of MC1 shape variation shows a broad overlap between all three groups (Figure 3). PC1 (25.7% of the total variation) shows variation in robusticity with negative scores associated with thinner shafts and relatively small articular surfaces, and positive scores with thicker shafts, relatively large articular surfaces, and a larger *m. opponens pollicis* insertion. PC2 (16.5% of the total variation) captures changes in distal MC1 shape with positive values indicating a more ulnarly deviated distal articular head, and rounder distal articular surface and a relatively larger and extended palmar radial condyle than the negative values.

**FIGURE 4:**
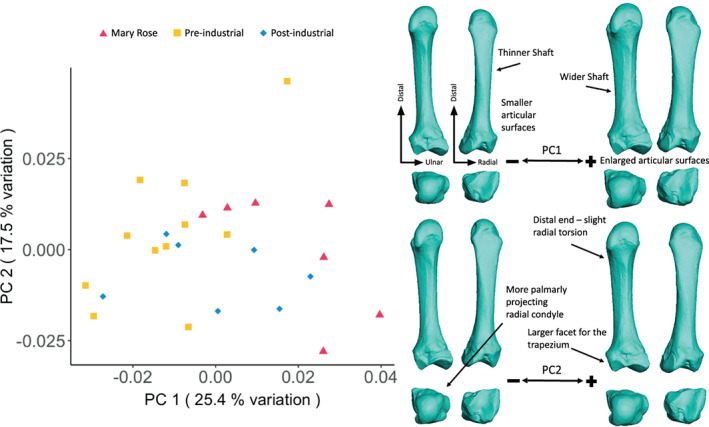
Second metacarpal external shape variation. On the left, PCA plot of PC1 and PC2, showing variation in the second metacarpal shape and, on the right, surface warps depicting the morphological changes captured by each extreme of the principal component axes, with shape changes labeled (top images show palmar [left] and dorsal [right] views; bottom images show distal [left] and proximal [right] views).

The PCA of MC2 shape variation (Figure 4) displays slight separation between the Pre‐industrial group and Mary Rose and Post‐industrial groups, with some areas of overlap. PC1 (25.4% of the total variation) reflects changes in external robusticity similar to that found for the MC1, and provides separation of the Pre‐industrial group (negative values) presenting a more gracile morphology than those from the Mary Rose (positive values) presenting a more robust morphology indicate a morphology with a more palmarly‐projecting distal radial condyle, facet for the trapezium, compared to the positive PC2 values showing a more radially‐projecting distal radial condyle, a distal end with slight radial torsion, and a larger facet for the trapezium.

**FIGURE 5:**
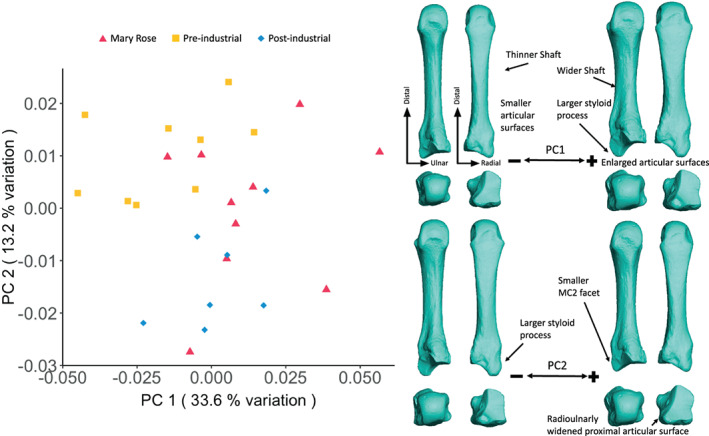
Third metacarpal external shape variation. On the left, PCA plot of PC1 and PC2, showing variation in third metacarpal shape and, on the right, surface warps depicting the morphological changes captured by each extreme of the principal component axes, with shape changes labeled (top images show palmar [left] and dorsal [right] views; bottom images show distal [left] and proximal [right] views).

While there is some overlap between groups, the PCA of MC3 shape variation (Figure 5) shows a degree of separation of the Pre‐industrial group from the Mary Rose and, especially, the Post‐industrial groups; with the Pre‐industrial group placed on the negative side of PC1 (33.6% of the total variation) and the positive side of PC2 (13.2% of the total variation). Negative PC1 scores are associated with a more gracile metacarpal with smaller articular surfaces and a thinner diaphysis, while positive PC1 scores are associated with a more robust metacarpal with enlarged articular surfaces, a wider diaphysis, and also a relatively large and proximally extended styloid process. Negative PC2 scores indicate an MC3 with a larger styloid process, and hence a larger MC2 facet, and a radio‐ulnarly thinner proximal articular surface. Positive values of PC2 indicate a smaller styloid process, a smaller MC2 facet, and an ulnarly‐deviated proximal end.

**FIGURE 6:**
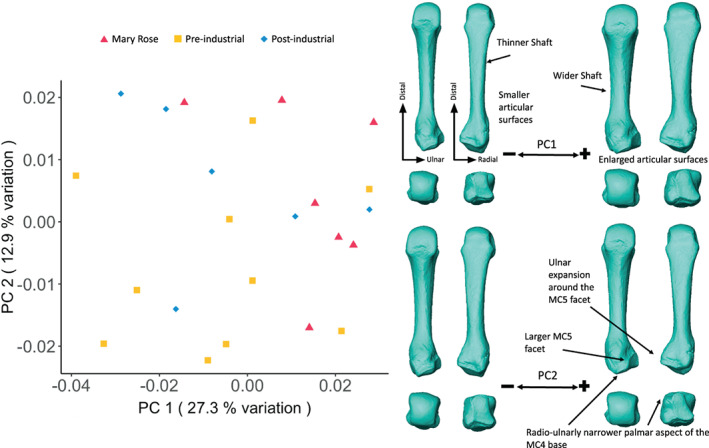
Fourth metacarpal external shape variation. On the left, PCA plot of PC1 and PC2, showing variation in the fourth metacarpal shape and, on the right, surface warps depicting the morphological changes captured by each extreme of the principal component axes, with shape changes labeled (top images show palmar [left] and dorsal [right] views; bottom images show distal [left] and proximal [right] views).

The PCA of MC4 shape variation (Figure 6) displays a substantial overlap between the groups with the Mary Rose group clustering at the positive end of PC1 (27.3% of the total variation), which shows a relatively more robust morphology with wider shaft, and larger articular surfaces than the negative end of PC1. There is overlap across all groups along PC2 (12.9% of the total variation), with negative values showing a MC4 that has a notably smaller MC5 articulation compared to the positive values of PC2 that show a MC4 with a larger MC5 articulation, pronounced radio‐ulnar expansion of the dorsal side of the MC4 base, and a radio‐ulnarly narrowed palmar aspect of the MC4 base.

**FIGURE 7:**
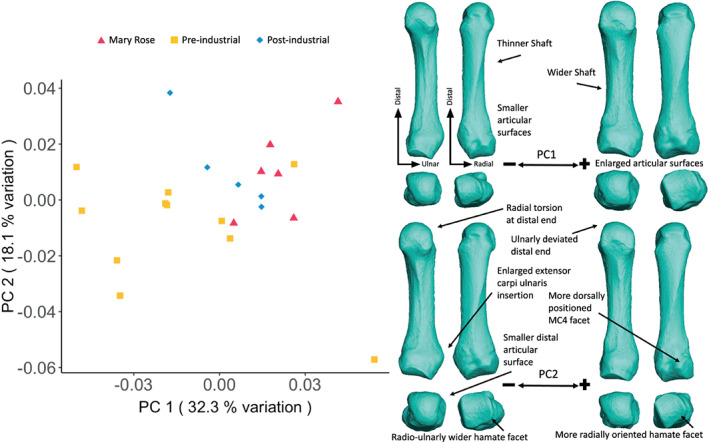
Fifth metacarpal external shape variation. On the left, PCA plot of PC1 and PC2, showing variation in the fifth metacarpal shape and, on the right, surface warps depicting the morphological changes captured by each extreme of the principal component axes, with shape changes labeled (top images show palmar [left] and dorsal [right] views; bottom images show distal [left] and proximal [right] views).

The PCA of MC5 shape variation (Figure 7) displays some slight separation between the groups along PC1 (32.3% of the total variation), however, there is much overlap. The Mary Rose individuals cluster with positive values of PC1, while those from the Pre‐industrial group showcase more negative values of PC1, with overlap between the two groups; and the Post‐industrial acting as an intermediate between the two. Negative PC1 scores are associated with gracile MC5s, with thinner shafts and smaller articular surfaces, while positive PC1 values indicate a morphology that is more robust, with wider shafts, and enlarged articular surfaces. PC2 (18.1% of the total variation) slightly separates the Mary Rose and Post‐industrial groups (positive side of PC2), from the Pre‐industrial group (negative side of PC2). Negative values of PC2 indicate MC5s with a radially‐deviated distal end, a smaller distal articular surface, an enlarged *extensor carpi ulnaris* insertion site, and a radio‐ulnarly wider articulation for the hamate. Positive PC2 values indicate an ulnarly‐deviated and larger distal end, a more dorsally‐positioned and proximodistal‐oriented MC4 facet, and a radio‐ulnarly narrower and more radially‐oriented hamate facet.

**FIGURE 8:**
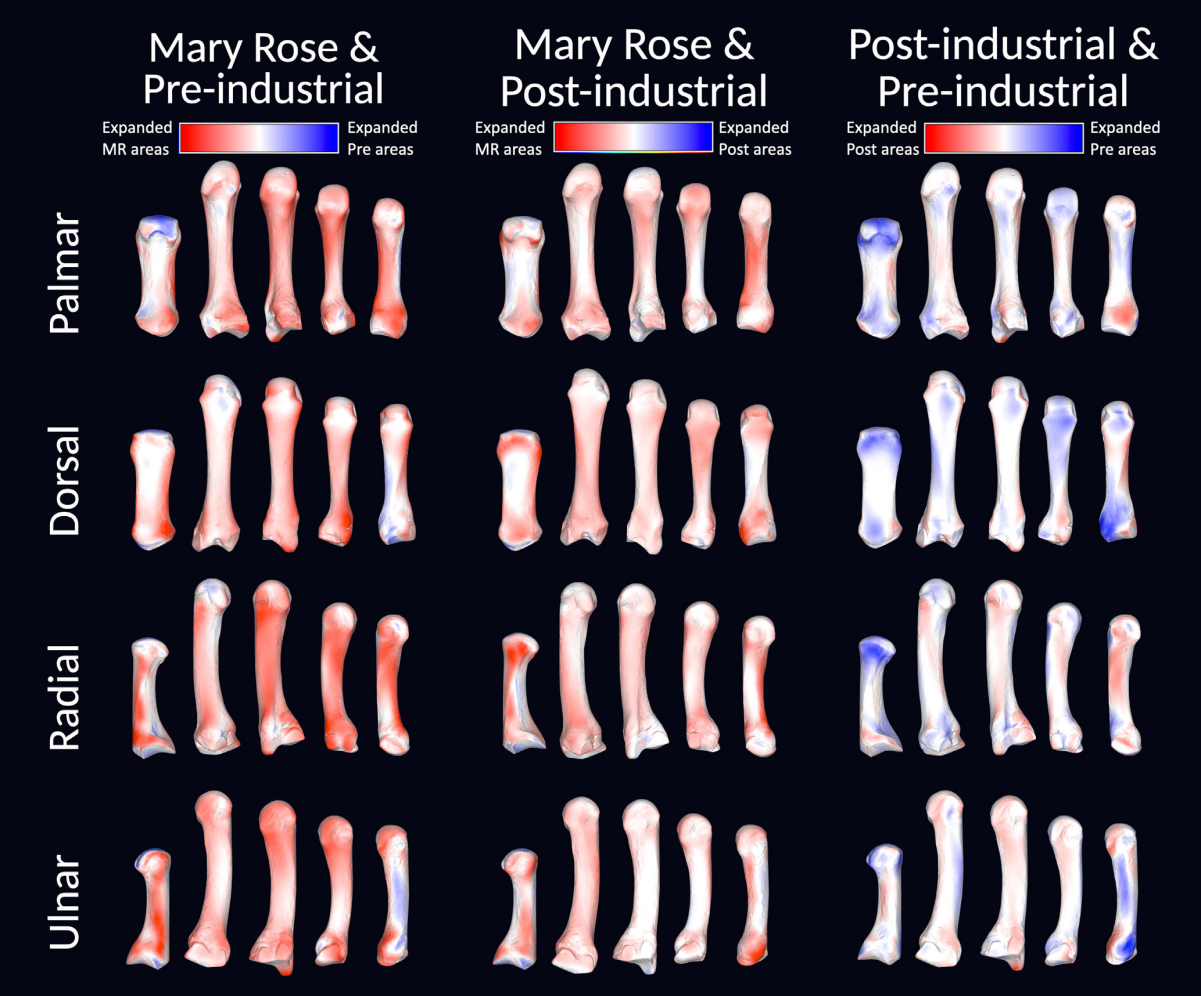
Comparisons of mean metacarpal shapes between groups. Metacarpal shape differences are depicted with distance heatmaps overlayed onto scaled warps of template specimens, showing the MC1 (left) to MC5 (right) for each group comparison. In the comparisons between the Mary Rose (MR) and Pre‐industrial, and between the Mary Rose and Post‐industrial, red indicates areas that are more pronounced or expanded on the Mary Rose metacarpal mean models, and blue indicates expanded areas on the Pre‐ and Post‐industrial mean models. In the Pre‐ and Post‐industrial comparison, red indicates areas more expanded on the Post‐industrial mean models and blue indicates pronounced areas on the Pre‐industrial mean models.

By comparing the mean shapes of each metacarpal between groups (Figure 8; see also Figure S7) we see that the greatest differences in mean shape are between the Mary Rose and Pre‐Industrial groups, however, there is much overlap. The differences highlighted by the Mary Rose and Pre‐industrial comparison are seemingly the result of more robust metacarpal morphology in the Mary Rose group, with wider diaphyses, enlarged articular surfaces, more prominent muscle insertion sites across the metacarpus (e.g., *m. opponens pollicis* insertion on the radial MC1 diaphysis), and a flatter distal articular surface (more domed in the Pre‐industrial group; see Figure S7). Differences between the Mary Rose and Post‐industrial groups' metacarpal shapes are less pronounced than with the Pre‐industrial group, however, the Mary Rose group is clearly, if only slightly, more robust in external morphology than the Post‐industrial group; with wider diaphyses and expanded regions of the proximal and distal ends across all metacarpals. There is less consistent differentiation between the mean shapes of the Pre‐ and Post‐industrial metacarpals, with the most pronounced differences occurring on the distal end of the MC1, across the MC5, and to the styloid process of the MC3 (pronounced in the Post‐industrial group).

Pairwise permutational ANOVAs of Procrustes shape coordinates are presented in Table 3 and highlight differences in terms of total metacarpal shape variation between groups. The Mary Rose group differs significantly in shape from the Pre‐industrial group for MC2–MC5. The Mary Rose group does not differ significantly from the Post‐industrial group for any metacarpal. Pre‐ and Post‐industrial groups only differ significantly in the shape of the MC3.

**TABLE 3:** Pairwise Procrustes ANOVA of distances between group metacarpal shapes, with a Bonferroni correction.

	Mary Rose—Pre		Mary Rose—Post		Pre—Post	
Metacarpal	Distance	*p*	Distance	*p*	Distance	*p*
MC1	0.033	0.450	0.033	0.638	0.022	>0.999
MC2	**0.033**	**0.002**	0.023	0.589	0.022	0.479
MC3	**0.034**	**0.001**	0.022	0.603	**0.031**	**0.046**
MC4	**0.029**	**0.043**	0.025	0.551	0.025	0.315
MC5	**0.042**	**0.003**	0.029	>0.999	0.035	0.184

*Note*: Bold = *p* ≤ 0.05.

### 3.2 Allometric patterns of metacarpal shape

We tested for allometric influence of metacarpal size on their external shape with permutational ANOVAs between Procrustes shape coordinates and measurements of size. The results indicate there is no significant effect of metacarpal size on whole bone morphology across all sampled metacarpals, with the result of using centroid size and maximum metacarpal length providing similar R^2^ and p values across metacarpals 1, 2, 3, and 5 (Table 4).

**TABLE 4:** Permutational Procrustes ANOVA of shape coordinates with centroid size and metacarpal length for each metacarpal, with a Bonferroni correction.

	Centroid size		Metacarpal length	
Metacarpal	*R* ^2^	*p*	*R* ^2^	*p*
MC1	0.098	0.181	0.087	0.301
MC2	0.069	0.342	0.076	0.197
MC3	0.040	>0.999	0.050	>0.999
MC4	0.028	>0.999	0.080	0.212
MC5	0.078	0.585	0.083	0.366

### 3.3 Analysis of metacarpal cross‐sectional geometry

**FIGURE 9:**
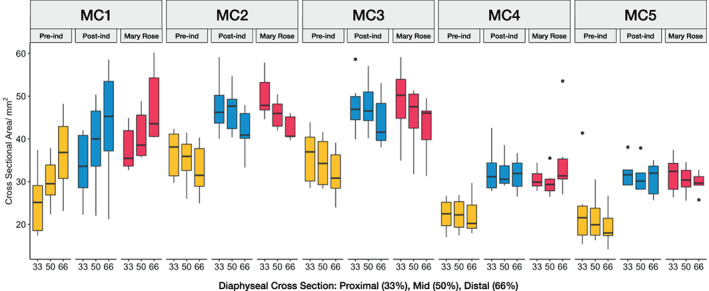
Diaphyseal cross‐sectional area (*CSA*) distribution in MC1‐MC5 at each cross‐section. From left to right, cross sections at 33% (proximal slice), 50% (mid‐diaphysis slice) and 66% (distal slice) are shown for each metacarpal and each human group. Note the general trend of greater *CSA* in the MC1‐MC3 versus MC4‐MC5.

The values of the polar moment of inertia (*J)* and cross‐sectional area (*CSA)* presented in this paper were not standardized by anybody measurements in order to preserve the absolute differences in CSA and J across the metacarpus (but see section 9 in Data S1 for results using CSG data standardized by metacarpal length). Variation in cross‐sectional area (*CSA*) reveals several distinct patterns that distinguish between human groups, across the metacarpus and along the diaphysis of each metacarpal (Figure 9). In each metacarpal, and at each position along the diaphysis, the Pre‐industrial group exhibits significantly lower *CSA* values than the Mary Rose group (*p* < 0.05; Table 5), and while the CSA values from the Post‐industrial group appear to be larger than those from the Pre‐industrial group, they are not significantly greater. There are three different patterns of *CSA* distribution along metacarpal diaphyses that are consistently present in each group. The first pattern is present in MC1 with smaller *CSA* values at the proximal end that progressively increase distally. The second pattern is present in MC2 and MC3, which express the opposite pattern with a decrease in *CSA* values from proximal to distal. The third pattern is present in MC4 and MC5 that exhibit similar *CSA* values along the diaphysis. Across the metacarpus there is also a clear decrease in *CSA* between MC1‐3 and MC4‐5 indicating stronger diaphyses in the former (Table 5), with this pattern being consistent within each group. Using a pooled sample, Wilcoxon rank sum tests show that in several cases these differences in *CSA* between metacarpals, at the same positions along the diaphysis, are statistically significant (Table S5).

**TABLE 5:** Differences in *J* and *CSA* values between human groups across all metacarpals.

		Proximal (33%)			Mid‐diaphyseal (50%)			Distal (66%)		
		MR	Pre	Post	MR	Pre	Post	MR	Pre	Post
	MR	‐	**0.030**	>0.999	‐	**0.036**	>0.999	‐	0.087	>0.999
MC1	Pre	**0.036**	‐	0.108	**0.036**	‐	0.264	0.180	‐	0.340
	Post	>0.999	**0.023**	‐	>0.999	**0.023**	‐	>0.999	0.110	‐
		MR	Pre	Post	MR	Pre	Post	MR	Pre	Post
	MR	‐	**0.004**	0.953	‐	**0.008**	>0.999	‐	**0.022**	>0.999
MC2	Pre	**<0.001**	‐	0.135	**<0.001**	‐	0.167	**<0.001**	‐	0.205
	Post	>0.999	**0.002**	‐	>0.999	**<0.001**	‐	>0.999	**0.005**	‐
		MR	Pre	Post	MR	Pre	Post	MR	Pre	Post
	MR	‐	**0.027**	>0.999	‐	**0.034**	>0.999	‐	**0.012**	>0.999
MC3	Pre	**0.002**	‐	0.099	**0.005**	‐	0.129	**<0.001**	‐	0.075
	Post	>0.999	**0.009**		>0.999	**0.002**	‐	>0.999	**0.001**	‐
		MR	Pre	Post	MR	Pre	Post	MR	Pre	Post
	MR	‐	**0.009**	>0.999	‐	**0.025**	>0.999	‐	**0.004**	0.703
MC4	Pre	**<0.001**	‐	**0.031**	**<0.001**	‐	0.235	**<0.001**	‐	0.185
	Post	>0.999	**<0.001**	‐	0.703	**<0.001**	‐	>0.999	**0.006**	‐
		MR	Pre	Post	MR	Pre	Post	MR	Pre	Post
	MR	‐	0.055	>0.999	‐	0.073	>0.999	‐	**0.021**	>0.999
MC5	Pre	**0.021**	‐	0.245	**0.021**	‐	0.145	**0.002**	‐	**0.028**
	Post	>0.999	**0.041**	‐	>0.999	**0.041**	‐	>0.999	**0.007**	‐

*Note*: Pairwise Wilcoxon rank sum tests with a Bonferroni correction. *J* on upper right (gray), cross‐sectional area (*CSA*) on lower left of each section. Bold = *p* ≤ 0.05.

Abbreviation: MR, Mary Rose.

**FIGURE 10:**
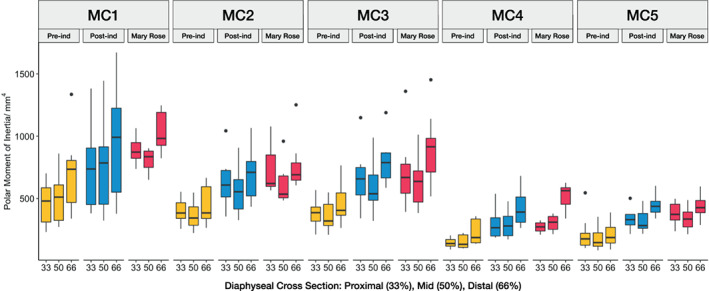
Diaphyseal polar moment of inertia (*J*) distribution across MC1‐MC5 at each cross‐section. From left to right, cross sections at 33% (proximal slice), 50% (mid‐diaphysis slice), and 66% (distal slice) are shown for each metacarpal and each human group. Note the general trend of greater *J* in the MC1‐MC3 versus MC4‐MC5.

Resistance to torsional deformation, measured as the polar moment of inertia or *J*, also reveals several patterns that distinguish between groups, across the metacarpus and along the diaphysis of each metacarpal (Figure 10). Similar to *CSA*, *J* is significantly lower in the Pre‐industrial group compared to broadly similar values in the Post‐industrial and Mary Rose groups (*p* < 0.05, Table 5). Across the metacarpus, MC1 has the highest *J*, followed by MC2 and MC3, and then MC4 and MC5 have the smallest values, with this pattern being consistent across each human group. Using a pooled sample, Wilcoxon rank sum tests show that this difference between MC1‐MC3 and MC4‐MC5 *J* values is statistically significant (Table S5). Within the diaphysis of each metacarpal, the distal end (66% slice) tends to have a higher resistance to torsion than the proximal or mid‐shaft regions, which tend to be similar.

### 3.4 Cross‐sectional geometry and external morphology correlations

We also tested for correlations between the cross‐sectional properties *CSA* and *J* with components of external morphology (based on PC1 and PC2 scores of Procrustes shape coordinates) in a pooled sampled for each metacarpal. *J* and PC1 are significantly correlated across all sampled cross‐sections of the MC2, MC3, and MC5 and the distal cross‐section of the MC4 (*p* < 0.05; Table 6), with the MC5 33% cross‐section exhibiting the strongest correlations between PC1 and *J* (*r* = 0.815). *CSA* is significantly correlated with all cross‐sections of the MC5, with the proximal and mid‐diaphyseal cross‐sections of the MC2 and only the proximal cross‐section of the MC3 (*p* < 0.05; Table S8). It is also worth noting that there are several correlations of PC1 with *J* and *CSA* that tend toward significance across MC1–MC4 (*p‐*values between 0.053 and 0.094; Table 6). Additionally, there are no significant correlations of PC2 with *CSA* and *J* across all metacarpals and cross‐sections (Table S9).

**TABLE 6:** Pearson's correlations of CSG and shape (PC1).

Metacarpal	Cross‐section	J		CSA	
		*r*	*p*	*r*	*p*
MC1	33	0.354	0.379	−0.015	>0.999
	50	0.502	0.073	0.333	0.453
	66	0.511	0.064	0.332	0.460
MC2	33	**0.709**	**<0.001**	**0.628**	**<0.001**
	50	**0.686**	**<0.001**	**0.556**	**0.012**
	66	**0.702**	**<0.001**	−0.710	0.053
MC3	33	**0.698**	**<0.001**	**0.527**	**0.017**
	50	**0.637**	**0.001**	0.442	0.071
	66	**0.699**	**<0.001**	0.437	0.083
MC4	33	0.390	0.197	0.155	>0.999
	50	0.457	0.085	0.157	>0.999
	66	**0.535**	**0.025**	0.235	0.842
MC5	33	**0.815**	**<0.001**	**0.691**	**<0.001**
	50	**0.676**	**0.002**	**0.613**	**0.007**
	66	**0.650**	**0.003**	**0.523**	**0.037**

*Note*: Correlations of the polar moment of inertia (*J*) and cross‐sectional area (*CSA*) with the first principal component of shape for all metacarpals, at each cross‐section, with Bonferroni corrections. Bold values = *p* ≤ 0.05.

## 4 DISCUSSION

This study aimed to investigate shape variation and correlation between external and internal morphology of metacarpals 1–5 in three temporo‐geographically diverse human groups, assumed to differ in their manual activities. Our prediction that the three sample groups would differ in terms of whole metacarpal shape was partially supported, as there were differences between the Mary Rose and Pre‐industrial non‐pollical metacarpals. We correctly predicted that there would be no allometric impact on whole bone shape, in line with previous research of hominoid metacarpal shape. However, our predictions about the CSG differences across the groups were only partially supported and may seemingly result from incorrect assumptions about the activity of both the Pre‐ and Post‐industrial groups, and/or how other (currently untestable) factors such as sex, age, and genetics may be influencing the results; and also the potential impact of neutral evolution driving greater variation in shape and structure in these geographically distinct groups. We tested the prediction that external shape would positively correlate with CSG properties and found strong evidence for a link between the two, specifically between shape changes linked with robusticity and the polar moment of inertia. Principal components of shape variation beyond the first and second are not discussed in this paper as there were no distinct patterns of group separation and few significant correlations between these other PCs with the CSG data from all sampled metacarpal cross‐sections; these lower components of shape variation may be driven by other, currently untestable, factors such as age and sex. Each set of results is discussed in more detail below.

### 4.1 Metacarpal external shape variation

Contrary to our null hypothesis, our 3D GM analysis revealed variation both within and between human groups and that this variation relates predominantly to shape changes associated with robusticity. These differences include increases in diaphysis width, articular surface size, and in elevations associated with entheses (e.g., the *m. opponens pollicis* insertion on the MC1). Expansion of these aspects of metacarpal morphology that reflect greater robusticity are thought to provide resistance to larger loads and to ultimately prevent bone failure under higher strain (Marzke & Marzke, 1987; Micklesfield et al., 2011; Nikander et al., 2010; Plochocki et al., 2006).

Beyond morphological features linked to robusticity, other shape differences (captured by PC2) have been previously linked with the stability and/or the range of motion across metacarpal joints. For example, the enlargement of the MC2 trapezium facet and MC3 styloid process may permit larger loads to stabilize the carpometacarpal joints during forceful precision and power grips and to avoid subluxation or fracture (Marzke & Marzke, 1987; Tocheri, 2007). Similarly, the MC1 shape analysis highlighted the larger and flatter distal articular surface and larger palmar radial condyle, which have been suggested to lower the range of motion and increase stability at the metacarpophalangeal joint and may increase the ability of the thumb to resist high loads commonly produced during powerful pad to pad precision grips (Galletta et al., 2019).

We found significant differences between groups in terms of both total shape variation (using Procrustes shape coordinates) and in its major axes of shape variation (PC1 and PC2), with the greatest differences in shape occurring between the Mary Rose and the Pre‐Industrial groups. These two groups differed primarily in their degree of overall robusticity, with the Mary Rose sample, comprising male medieval soldiers and sailors, possessing metacarpals with greater external robusticity, which may be a potential response to engaging in more intense habitual manual activities than the Pre‐Industrial group (Plochocki et al., 2006). Additionally, the mean model of the Mary Rose MC5 has a more ulnarly‐deviated distal head, a more radially‐oriented hamate facet, and larger entheses to the fifth digit (Figure S7) relative to the MC5 of Pre‐Industrial individuals. These morphological features may suggest more frequent use of powerful hand grips requiring greater supination of the fifth digit when the fingers are flexed, such as during a power ‘squeeze’ grip (Marzke et al., 1992). As soldiers and sailors on board the Mary Rose, repetitive and heavy manual labor during daily activities would be expected (Stirland, 2012). However, it is important to acknowledge that the Mary Rose sample is assumed to be comprised of males only while sex remains unknown and likely mixed for our Pre‐ and Post‐Industrial samples; and as such we are unable to test for the impact of sex on this sample.

Our inability to assess variation in shape related to sex is one of the main limitations of this study. Differences in the external morphology of human male and female bones have been attributed to the sexual division of labor, such as differences in entheseal patterns across the hand of males and females related to occupation and habitual grips (Karakostis & Hotz, 2022), and entheseal changes of the upper and lower limbs suggesting sex divisions across multiple populations (e.g., Havelková et al., 2011; Laffranchi et al., 2020; Villotte et al., 2010). Thus, the more robust nature of the Mary Rose metacarpals and their significant separation from the Pre‐industrial metacarpals may be linked to sex differences in manual loading rather than population‐level differences. While the men of the Mary Rose represent a unique archeological sample of young male individuals undertaking high‐intensity manual labor (Stirland, 2012), if this sample was more representative of a typical Medieval population and included some females, it is likely that differences in shape between the Mary Rose and Pre‐industrial groups would be more limited.

Unlike sex, we were able to test the potential impact of size on metacarpal external shape. In support of our third hypothesis, we found no evidence to support an allometric relationship between metacarpal size (using either centroid size or maximum metacarpal length) and external shape (using Procrustes aligned coordinates). Previous studies of metacarpal shape have identified weak or no allometric relationships between shape and size in hominoids (e.g., Bardo et al., 2020; Bowland et al., 2021; Galletta et al., 2019: Morley et al., 2020; Niewoehner, 2005), and suggests that variation in activity, among other factors such as genetics, may be one of the main factors driving variation in metacarpal shape in this study sample. However, without detailed profiles of individuals within our study sample, we must assume that differences in sex, age, hormones (in addition to activity), and the interaction between them all, may also be in part responsible for the morphological differences we find across our sample.

### 4.2 Variation in metacarpal cross‐sectional geometry

Contrary to our predictions, group differences in CSG revealed that the Mary Rose and Post‐industrial groups (rather than the Mary Rose and Pre‐Industrial groups) had metacarpals with greater resistance to torsional deformation (inferred by *J*) and axial compression and tension (inferred by *CSA*) (Lieberman et al., 2004) compared with the Pre‐industrial group. These differences suggest that the Mary Rose and Post‐Industrial groups were habitually undertaking manual activities of a higher intensity than the Pre‐industrial group. This result is not surprising for the Mary Rose sample given they are known to be medieval sailors and soldiers, and as such would have undertaken routine heavy manual loading (Stirland, 2012), but is unexpected for the Post‐Industrial group. Despite limited information about the demographics of our Pre‐ and Post‐Industrial samples, we predicted that the Post‐industrial group may have had a greater reliance on industrial tools and a decreased reliance on physical prowess, while the Pre‐industrial humans would engage in more frequent and/or high‐intensity manual behaviors. Our CSG results, highlighting lower levels in the Pre‐industrial group and higher levels in the Post‐industrial group, indicate that the assumptions made about these groups may need to be re‐evaluated for future investigations. It may be that these Post‐industrial individuals were undertaking heavy manual loading and using powerful and forceful grips at similar levels to the men of the Mary Rose, while the Pre‐industrial individuals may have undertaken lower‐intensity manual loading.

As previously stated, there is conceivably a sex bias within this study. In addition to the differences between the Mary Rose and other groups being the potential result of a sexual division of labor, female long bones have been previously documented as possessing less cortical bone mass relative to body size and bone size compared to male long bones (Jepsen et al., 2015), with the male skeleton being described as denser than the female skeleton (Trotter et al., 1960). In addition, although the Mary Rose sample is considered to be comprised of young (~19–29 years of age) males, the age of our Pre‐ and Post‐Industrial samples is unknown and thus we cannot rule out the effect of age on both internal and external aspects of bone structure, including decrease in bone density (e.g., Trotter et al., 1960), cortical thinning (e.g., Maggio et al., 1997), and entheseal changes (e.g., Villotte et al., 2022) that are associated with the aging process.

The distribution of cross‐sectional properties, *J* and *CSA*, across the metacarpus highlighted two distinct groupings: the MC1–3, and MC4–5. The significant differences between metacarpals may be a result of their relative importance and use during habitual manipulation. The human thumb has a major and unique role in many different types of forceful precision and power grips (e.g., Hamrick et al., 1998; Marzke, 1997; Marzke et al., 1992; Napier, 1956; Rolian et al., 2011; Williams‐Hatala et al., 2018). For all human groups, the MC1 has *J* and *CSA* values that were similar, especially for the distal‐most (66%) cross‐section, to those of the MC2 and MC3, despite being a much shorter bone in proximodistal length. Thus, relative to metacarpal length, the MC1 in humans provides comparatively higher *CSA* and *J* compared to the non‐pollical metacarpals. The largest values of *J* and *CSA* at the distal‐most cross‐section of the MC1 also reflect the prominent enthesis for the *m. opponens pollicis* in many specimens. This muscle is critical for flexing and abducting the MC1, and facilitates the opposition of thumb and finger pads, which in turn provides humans with the ability to grip and control large objects with one hand (Marzke et al., 1999; Smutz et al., 1998).

The MC2 and MC3 are two of the most stable metacarpals within the hand, with narrow ranges of motion at the carpometacarpal joints, providing an anchor for the thumb to rotate around with contraction of the *m. adductor pollicis* (El‐shennawy et al., 2001). The index and middle digits are also most frequently involved in precision grips with the thumb (Dollar, 2014; Marzke, 1997). Moreover, experimental studies show that the index and middle digits (as well as the thumb) experience the highest loads during both static and dynamic gripping (Gurram et al., 1995; Williams‐Hatala et al., 2018). The higher loading combined with more frequent use of both digits likely explains their large metacarpal *CSA* values (similar to that of the MC1) compared with the MC4 and MC5.

The fifth finger has been shown to play an important stabilizing role during power‐squeeze grips and precision grips (Key et al., 2019; Marzke et al., 1998) and the MC5 is considered the most robust of the non‐pollical metacarpals (MC2‐MC5) relative to length (Marzke et al., 1992). However, the torsional/bending strength of the MC4 and MC5, inferred by a polar stress–strain index, has been previously shown to be less than that of the MC1–MC3 (Wong et al., 2018). Thus, it was not surprising that the values of *CSA* and *J* were significantly lower in the MC4 and MC5 compared to the MC1–MC3 in a pooled human sample (Table S5). Further, this pattern of metacarpals one, two, and three having significantly higher *CSA* and *J* was consistent across the Mary Rose and Pre‐industrial human groups despite presumed differences in habitual hand use, but not within the Post‐industrial group (Tables S6–S8).

### 4.3 Correlating whole metacarpal shape with diaphyseal structure

Correlations between *J* and *CSA* with the first two principal components of shape highlight that a change in metacarpal morphology from gracile to robust is strongly correlated with an increase in the biomechanical strength of bone. We found a stronger link between increasing external robusticity and increasing resistance to torsion (inferred from *J*) compared to increasing resistance to axial compression and tension (inferred from *CSA*). Thus, metacarpals with traditional attributes of a robust form, such as a wider diaphysis and expanded articular surfaces, are likely to have an internal cortical structure more suited to withstanding higher mechanical loads, particularly to torsion (e.g., Marzke & Marzke, 1987; Micklesfield et al., 2011; Nikander et al., 2010; Plochocki et al., 2006). Furthermore, while the non‐pollical metacarpals (MC2–MC5) presented many significant correlations of PC1 with *J* and *CSA*, the MC1 shows no significant correlations between PC1 and cross‐sectional properties, and could imply that the external morphology of the pollical metacarpal has a different relationship with internal cortical structure compared to the non‐pollical metacarpals; something that could perhaps be related to the developmental uniqueness of the thumb in comparison to the palmar fingers (Morrish & Hlusko, 2014; Reno et al., 2008).

While significant correlations were found between PC1 scores from the shape analysis and most of the cross‐sectional properties across the non‐pollical metacarpals, PC2 is not significantly correlated with either *J* or *CSA* across all sampled metacarpals. This result indicates that the changes in shape captured by PC2, such as alterations to articular surface orientations or the relative size of condyles, are not correlated with diaphyseal cortical bone modeling. As some areas of external shape, particularly the morphology of articulations, have been shown to be more stable throughout life even with changes in loading, age or body mass (Lieberman et al., 2001; Ruff et al., 1991), it may be more likely that the shape variation highlighted by PC2 is the result of intrinsic factors not linked to behavior such as hormones, sex, and/or genetic variation (Klingenberg, 2014). Future investigations on samples with available data on the biological profiles of all sampled individuals could test if there is an influence of sex or age, or the interaction between them, on these highlighted aspects of metacarpus shape variation.

## 5 CONCLUSIONS

The analysis of metacarpal external shape provided some group separation based on the difference in observable robusticity (related to diaphyseal width and articular surface size), and the patterns of cross‐sectional properties along the metacarpus were largely the same across all sampled groups, however the magnitudes of the cross‐sectional area and polar moment of inertia differed between groups. We suggest that group differences in habitual manual activity are driving these differences in external and internal robusticity, particularly between the Mary Rose and Pre‐industrial groups. The repetitive and heavy manual loading experienced by the sailors and soldiers of the Mary Rose likely resulted in visibly and structurally more robust metacarpals that are able to withstand larger levels of torsion, and axial compression and tension.

This study also presented evidence for strong relationships between increasing indicators of robusticity—such as increasing articular surface area, enlarging specific bony elevations associated with entheses, and a wider diaphysis—with increasing the mechanical strength of metacarpals. Thus, features of robusticity may be useful indicators of bone strength, particularly in torsion. Further incorporation of additional methods to capture bone structural variation (both external and internal) and the utilization of a larger sample of humans, ideally with associated profiles, will facilitate greater understanding of the manner in which activity impacts bone structure and aid in the production of more accurate reconstructions of behavior in the past.

## AUTHOR CONTRIBUTIONS

**Samuel B. Tanner:** Conceptualization (lead); data curation (lead); formal analysis (lead); investigation (lead); methodology (equal); project administration (lead); visualization (lead); writing – original draft (lead); writing – review and editing (lead). **Ameline Bardo:** Formal analysis (supporting); visualization (supporting); writing – review and editing (supporting). **Thomas W. Davies:** Formal analysis (supporting); investigation (supporting); methodology (equal); visualization (supporting); writing – review and editing (supporting). **Christopher J. Dunmore:** Formal analysis (supporting); methodology (supporting); software (supporting); writing – review and editing (equal). **Richard E. Johnston:** Resources (supporting); writing – review and editing (supporting). **Nicholas J. Owen:** Resources (supporting); writing – review and editing (supporting). **Tracy L. Kivell:** Formal analysis (supporting); supervision (supporting); writing – original draft (supporting); writing – review and editing (equal). **Matthew M. Skinner:** Conceptualization (supporting); formal analysis (equal); methodology (equal); project administration (supporting); resources (equal); software (lead); supervision (lead); visualization (equal); writing – original draft (supporting); writing – review and editing (equal).

## CONFLICT OF INTEREST STATEMENT

The authors declare that there is no conflict of interest.

## Supporting information


**Data S1:** Supporting Information.

## Data Availability

The raw data that support the findings of this study are available on request from the corresponding author. The microCT data cannot be made publicly available due to restrictions of the respective curatorial institutions.
