# Supplementary material for: Variation and covariation of external shape and cross‐sectional geometry in the human metacarpus
Source: Am J Biol Anthropol. 2023 Nov 6;183(2):e24866. doi: 10.1002/ajpa.24866 (PMC10952563; doi:10.1002/ajpa.24866)
Supplement: Supplementary file 1 — Data S1: Supporting Information. [file AJPA-183-0-s001.docx]

**Supplementary Information**

1. Study sample

Supplementary Table 1 presents a comprehensive breakdown of the study sample by individual populations. The Mary Rose and Post-industrial groups are both made up of metacarpals from one skeletal collection (the Mary Rose shipwreck and a German cemetery, respectively), while the Pre-industrial group is made up metacarpals from six distinct temporally and spatially separated groups of humans.

Supplementary Table 1. Sample distribution by individual populations

| Group | Metacarpal Number | | | | |
| --- | --- | --- | --- | --- | --- |
|  | 1 | 2 | 3 | 4 | 5 |
| Mary Rose | 5 | 7 | 10 | 7 | 6 |
| **Göttingen Cemetery | 6 | 7 | 7 | 6 | 5 |
| *Nubian | 1 | 1 | 1 | 1 | 1 |
| *Inuit | 1 | 1 | 1 | 1 | 1 |
| *Amerindian | 2 | 1 | 0 | 1 | 1 |
| *Moriori | 2 | 2 | 2 | 2 | 2 |
| *Australian | 0 | 2 | 2 | 2 | 2 |
| *Tierra del Fuego | 3 | 4 | 3 | 4 | 4 |
| Total | 20 | 25 | 26 | 23 | 22 |

*These groups make up the Pre-Industrial sample, and ** make up the Post-Industrial sample

2. Reorientation Error in Avizo 6.3

Supplementary Table 2 presents the results of conducting 10 repeats of an MC1 reorientation in Avizo 6.3, with several days separating each repeat. This was conducted as the orientation of a bone has been shown to significantly affect the CSG values calculated, and as the process of reorienting each bone was conducted by hand, and by the same individual the reliability of this process was questioned. The results of this intra-observer error test suggest that the reorientation of metacarpals into a standard palmar position was conducted reliably and in a repeatable manner as the percentage error of CSA, Imax and Imin are within the range of 0.119-0.309% difference across all three variables and slices. This implies that any variation in cross-sectional properties across the sample is unlikely to result from reorientation error, but instead indicate something about the biomechanical properties of the bone.

Supplementary Table 2. Reorientation Error Analysis with the mean percentage error for all cross-sections and for all geometric properties calculated.

| Cross-Section | CSA (% error) | Imax (% error) | Imin (% error) |
| --- | --- | --- | --- |
| 33 | 0.215 | 0.248 | 0.299 |
| 50 | 0.309 | 0.199 | 0.240 |
| 66 | 0.237 | 0.258 | 0.203 |

3. Landmarking metacarpals

Supplementary Figure 1 presents the landmarking protocol used for the first metacarpal (images were created using a left template specimen).

Order of connecting the main landmarks to create the curves (pictured below in blue): Proximal Curve – 1, 2, 1; Distal Curve – 3, 4, 5, 6, 7, 3.


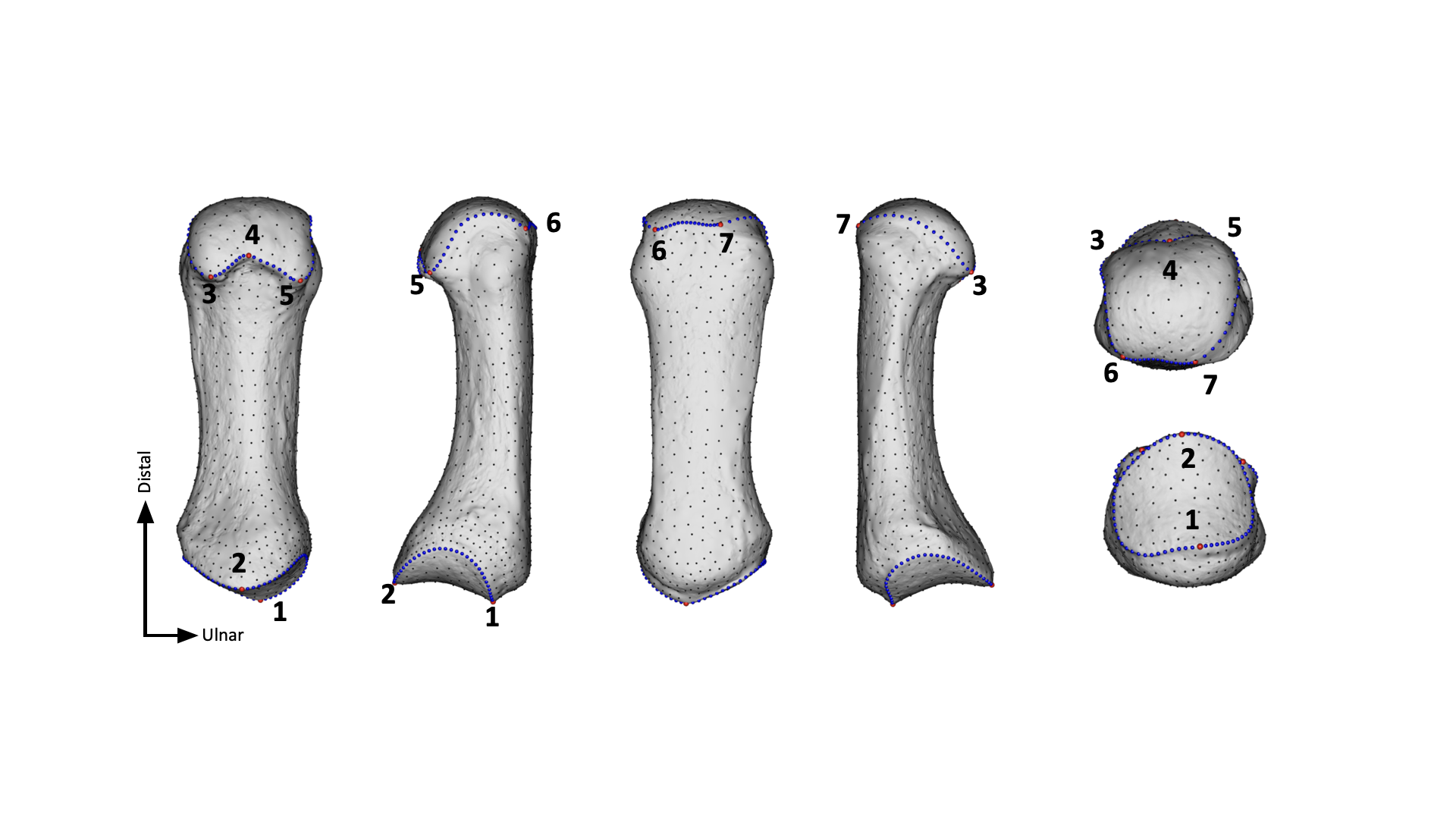


Supplementary Figure 1. Landmarking Protocol for the first metacarpal, on a left sided model. Highlighted are the fixed (anatomical) landmarks (red; labelled 1-7), the sliding semi-landmarks on curves (blue), and the sliding semi-landmarks on the surface (black; these were only placed once onto a template specimen). Left – Right: palmar, ulnar, dorsal, radial, distal (top), proximal (bottom).

Supplementary Figure 2 presents the landmarking protocol used for the second metacarpal (images were created using a left specimen).

Order of connecting the main landmarks to create the curves (pictured below in blue): Proximal Curve – 1, 2, 3, 4, 1; Distal Curve –5, 6, 7, 8, 5; Trapezium Facet Curve – 4, 1; MC3 Facet Curve – 2, 3.


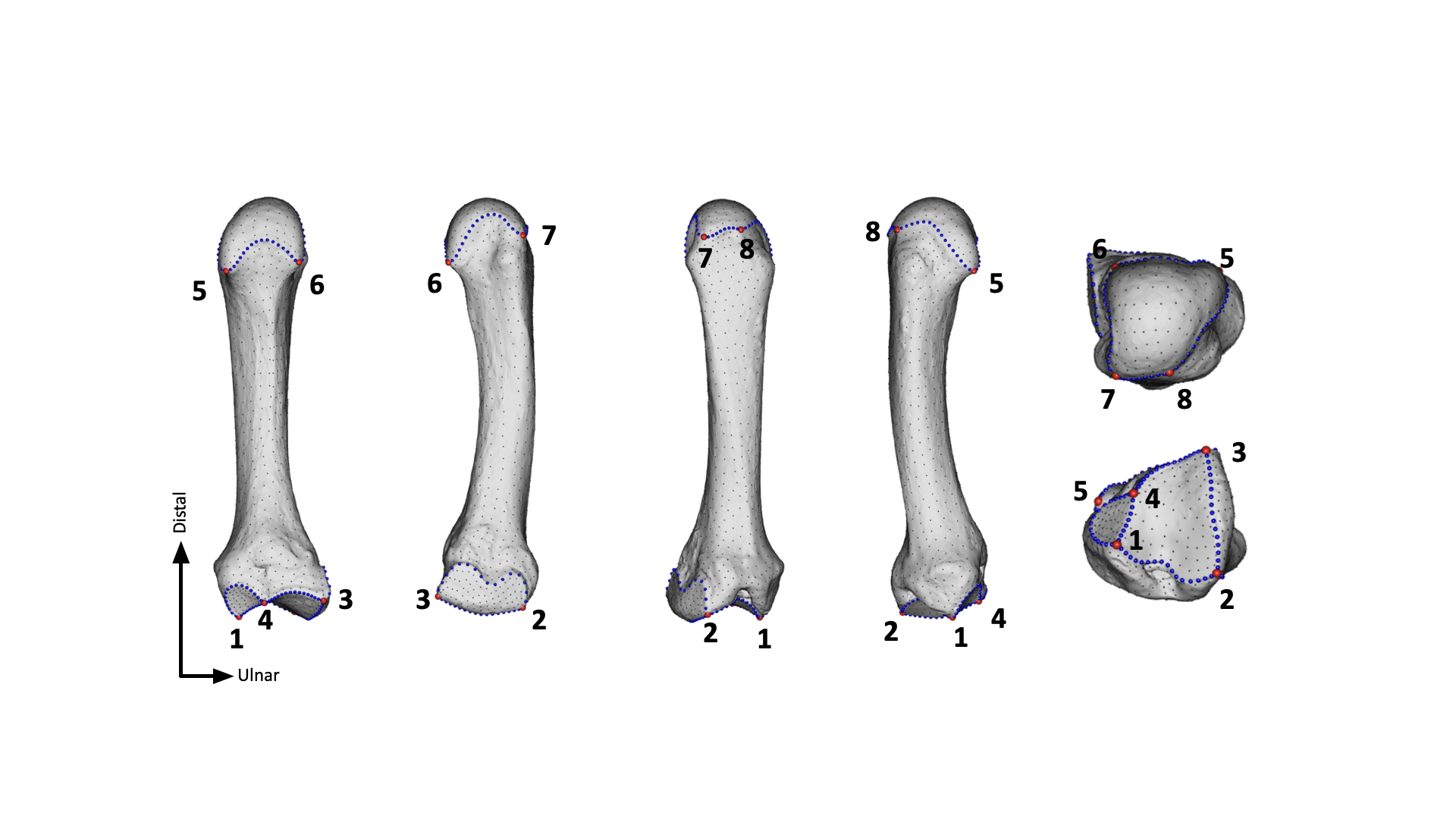


Supplementary Figure 2. Landmarking Protocol for the second metacarpal, on a left sided model. Highlighted are the fixed (anatomical) landmarks (red; labelled 1-8), the sliding semi-landmarks on curves (blue), and the sliding semi-landmarks on the surface (black; these were only placed once onto a template specimen). Left – Right: palmar, ulnar, dorsal, radial, distal (top), proximal (bottom).

Supplementary Figure 3 presents the landmarking protocol used for the third metacarpal (images were created using a left specimen).

Order of connecting the main landmarks to create the curves (pictured below in blue): Proximal Curve – 1, 2, 3, 4, 1; Distal Curve –5, 6, 7, 8, 5; MC2 Facet Curve – 2, 1. Note that on the ulnar side of the proximal end, the articulations for the fourth metacarpal are not landmarked. This was due to the high variability of those articulations, appearing as one (as in the example below) or two separate articulations (see Supplementary Section 9.3) across many of the sampled individuals, which made the process of repeatably landmarking the MC4 facets impossible; as such they were excluded from the landmarking process.


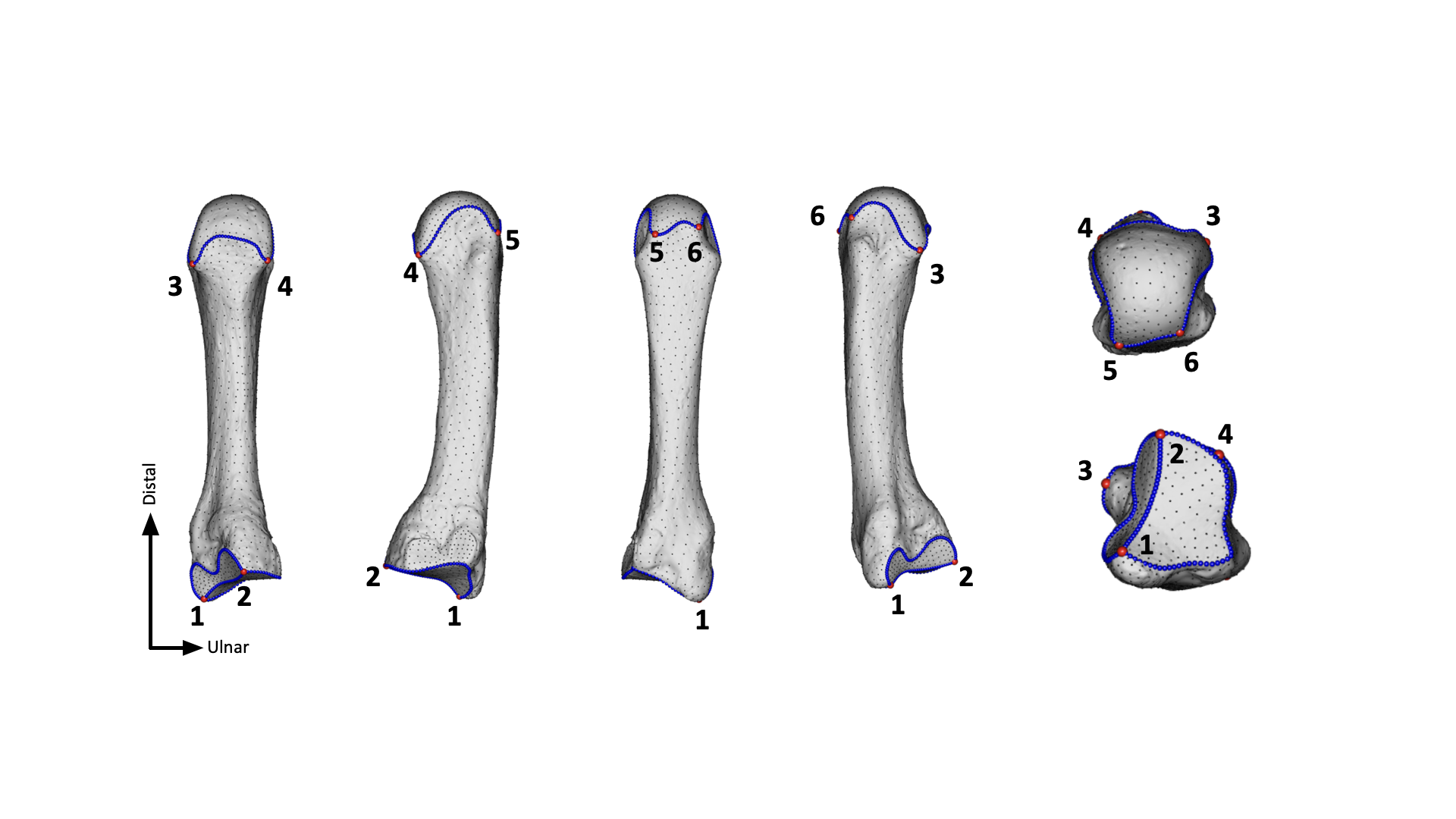


Supplementary Figure 3. Landmarking Protocol for the third metacarpal, on a left sided model. Highlighted are the fixed (anatomical) landmarks (red; labelled 1-6), the sliding semi-landmarks on curves (blue), and the sliding semi-landmarks on the surface (black; these were only placed once onto a template specimen). Left – Right: palmar, ulnar, dorsal, radial, distal (top), proximal (bottom).

Supplementary Figure 4 presents the landmarking protocol used for the fourth metacarpal (images were created using a left specimen).

Order of connecting the main landmarks to create the curves (pictured below in blue): Proximal Curve – 1, 2, 1; Distal Curve – 3, 4, 5, 6, 3; MC5 Facet Curve – 1, 2. Note that on the radial side of the proximal end, the articulations for the third metacarpal are not landmarked. This was due to the high variability of those articulations, appearing as one (see Supplementary Section 9.4) or two separate articulations (as in the example below) across many of the sampled individuals, which made the process of repeatably landmarking the MC4 facets impossible; as such they were excluded from the landmarking process.


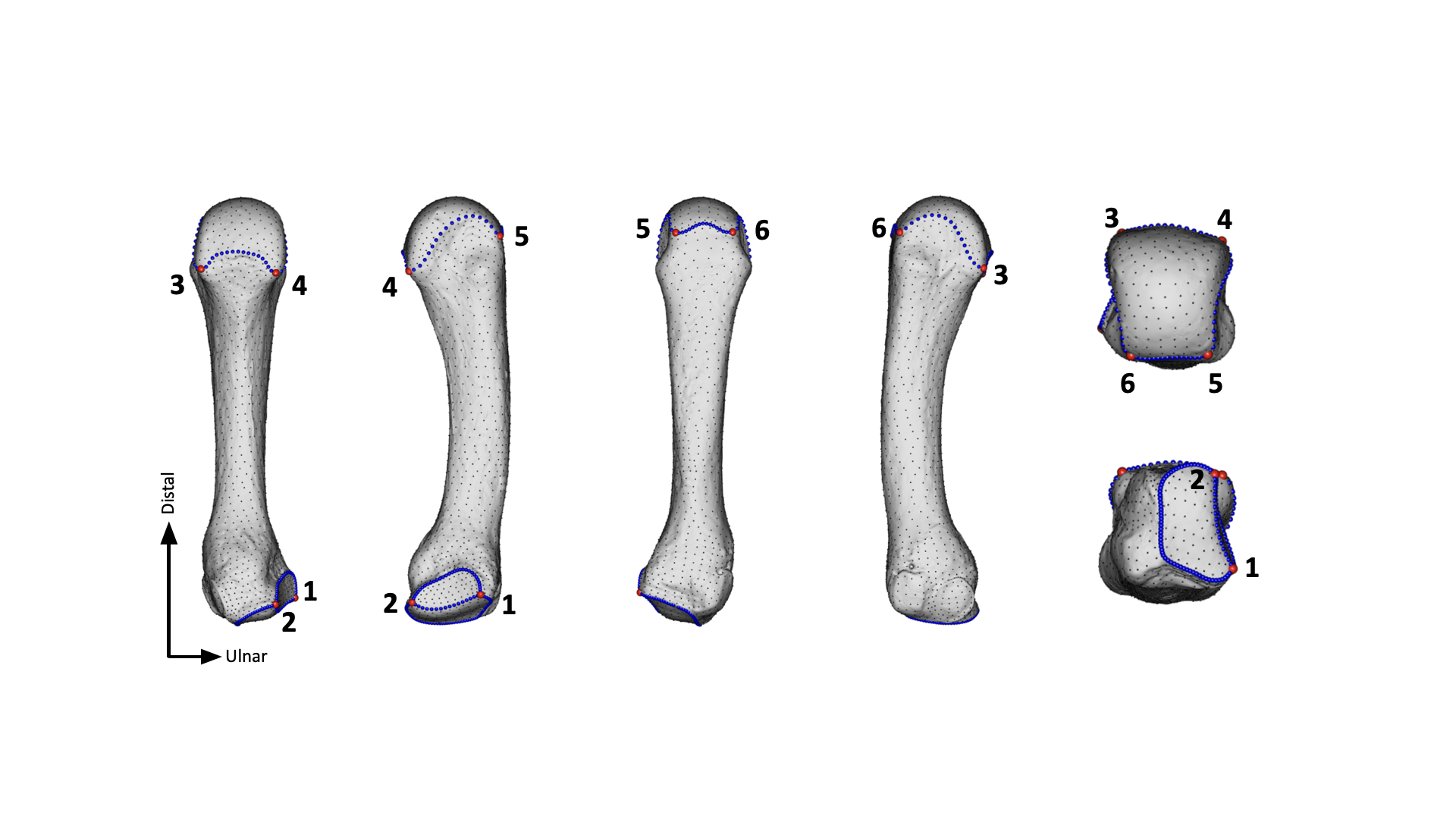


Supplementary Figure 4. Landmarking Protocol for the fourth metacarpal, on a left sided model. Highlighted are the fixed (anatomical) landmarks (red; labelled 1-6), the sliding semi-landmarks on curves (blue), and the sliding semi-landmarks on the surface (black; these were only placed once onto a template specimen). Left – Right: palmar, ulnar, dorsal, radial, distal (top), proximal (bottom).

Supplementary Figure 5 presents the landmarking protocol used for the fifth metacarpal (images were created using a left template specimen).

Order of connecting the main landmarks to create the curves (pictured below in blue): Proximal Curve – 1, 2, 1; Distal Curve –3, 4, 5, 6, 3; MC4 Facet Curve – 2, 1.


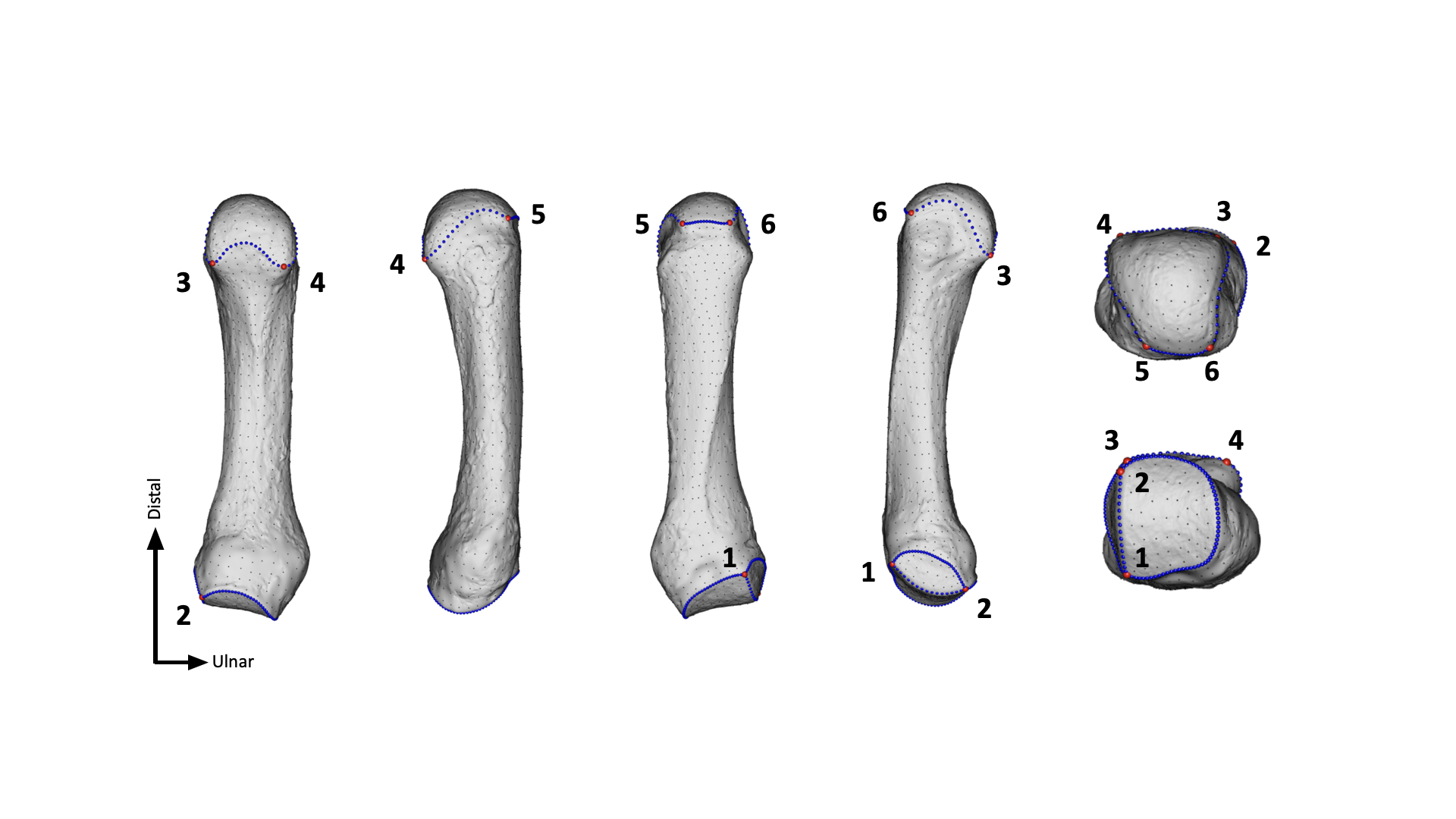


Supplementary Figure 5. Landmarking Protocol for the fifth metacarpal, on a left sided model. Highlighted are the fixed (anatomical) landmarks (red; labelled 1-6), the sliding semi-landmarks on curves (blue), and the sliding semi-landmarks on the surface (black; these were only placed once onto a template specimen). Left – Right: palmar, ulnar, dorsal, radial, distal (top), proximal (bottom).

Supplementary Figure 6 presents the results of conducting repeatability tests (both inter- and intra-observer error testing) on five different fifth metacarpals. Each metacarpal was landmarked 5 times by one author (ST) and once by a peer (NGS), with one day between repeats. After a Procrustes transformation a PCA was run on the data. Each colour represents a different specimen, with the interobserver repeats appearing as darker shades of the colours of each specimen. The first two PCs (total 78.3% of the variance) show that the variation among repetitions of the same specimens is lower than that between specimens.


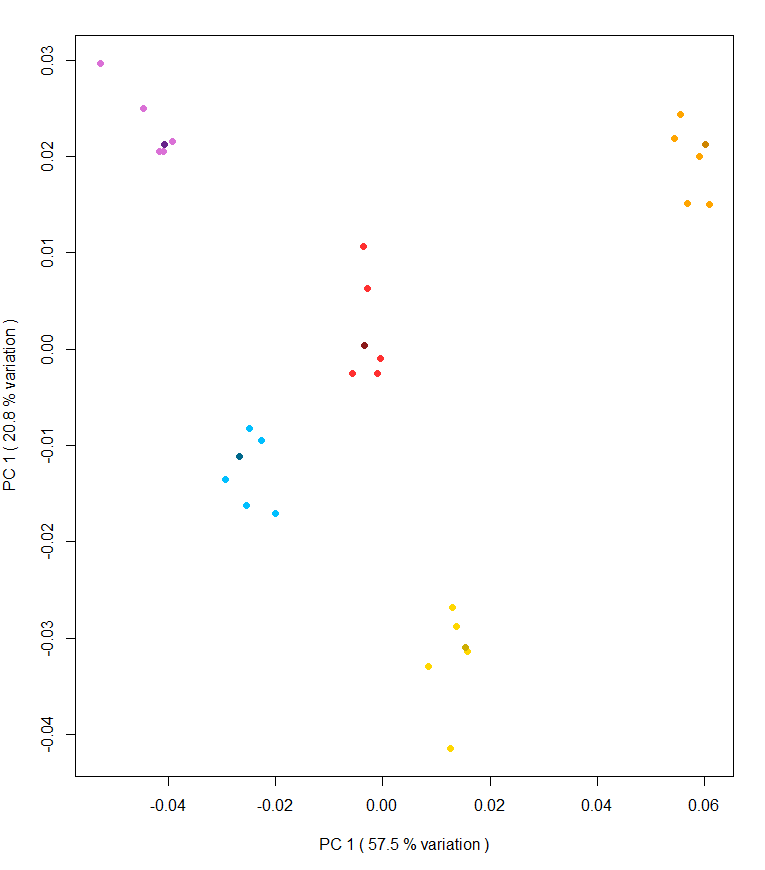


Supplementary Figure 6. PCA of Landmarking Intra- and Inter-observer error repeat testing of fifth metacarpals using fixed anatomical landmarks. PCs 1 and 2 display 78.3% of the variation in the repeats sample. Each colour represents a different specimen, and the singular darker shade of each colour represents the inter-observer repeat of landmarking (conducted by NGS).

Supplementary Table 3 presents the statistical analysis of the Euclidean distance between the means of each specimen repeated five times by one author (ST) for intra-observer testing, and those repeated once by a peer (NGS) for inter-observer testing. The results show that the inter-observer repeats are not statistically significantly different from those placed by me (ST).

Supplementary Table 3. Landmarking Intra- and Inter-observer error statistical analysis, using pairwise Euclidean distance between repeats. Bold values indicate *p* ≤ 0.05

|  | 2 | 3 | 4 | 5 | 1R | 2R | 3R | 4R | 5R |
| --- | --- | --- | --- | --- | --- | --- | --- | --- | --- |
| 1 | **0.002** | **0.003** | 0.343 | 0.675 | >0.999 | **0.020** | 0.060 | 0.786 | 0.957 |
| 2 |  | 0.725 | **0.000** | **0.001** | **0.016** | >0.999 | 0.974 | **0.000** | **0.016** |
| 3 |  |  | **0.000** | **0.003** | 0.052 | 0.962 | >0.999 | **0.005** | **0.042** |
| 4 |  |  |  | 0.368 | 0.823 | **0.000** | **0.004** | >0.999 | 0.855 |
| 5 |  |  |  |  | 0.953 | **0.011** | **0.040** | 0.852 | >0.999 |
| 1R |  |  |  |  |  | 0.227 | 0.431 | 0.525 | 0.730 |
| 2R |  |  |  |  |  |  | 0.748 | 0.022 | 0.203 |
| 3R |  |  |  |  |  |  |  | 0.129 | 0.388 |
| 4R |  |  |  |  |  |  |  |  | 0.596 |

1 – 5 represents the mean shape of each specimen that was repeated.

A number followed by R is used to indicate the specimens that were repeated by a peer for the interobserver error testing.

4. Normality check of cross-sectional geometry data.

Supplementary Table 4 Showcases the p value results from Shapiro-Wilks tests of the distribution of each cross-sectional property at each slice and each metacarpal sampled. Some p values are significant (p<0.05), indicating that there are 2 data distributions that are not normally distributed. Rather than transform the data points to make them more normally distributed it was decided to instead conduct any statistical analysis with the cross-sectional geometry data using non-parametric tests, such as the Wilcoxon sign rank test.

Supplementary Table 4. Test of normality for each metacarpal, slice, for both the polar moment of inertia and cross-sectional area. Bold values are used to denote where p <0.05.

| Metacarpal | 33% Slice | |  | 50% Slice | |  | 66% Slice | |
| --- | --- | --- | --- | --- | --- | --- | --- | --- |
|  | J | CSA |  | J | CSA |  | J | CSA |
| 1st | 0.620 | 0.262 |  | 0.240 | 0.950 |  | 0.580 | 0.457 |
| 2nd | 0.220 | 0.350 |  | 0.246 | 0.937 |  | 0.319 | 0.218 |
| 3rd | 0.080 | 0.565 |  | 0.444 | 0.325 |  | 0.737 | 0.662 |
| 4th | **0.043** | 0.690 |  | 0.136 | 0.460 |  | 0.252 | **0.012** |
| 5th | 0.199 | 0.816 |  | 0.308 | 0.183 |  | 0.374 | 0.108 |

5. Metacarpal group mean models

Supplementary Figure 7 showcases the mean metacarpus models for each sampled group. The differences between groups are extremely subtle, with the only discernible differences being the more robust shape of the Mary Rose metacarpals, and any others are much harder to interpret. So, while these models are informative, it was decided that heat maps of distance between models overlayed onto the specimens would be more useful to more appropriately highlight areas of difference in pairwise group comparisons (Fig.8 in-text).


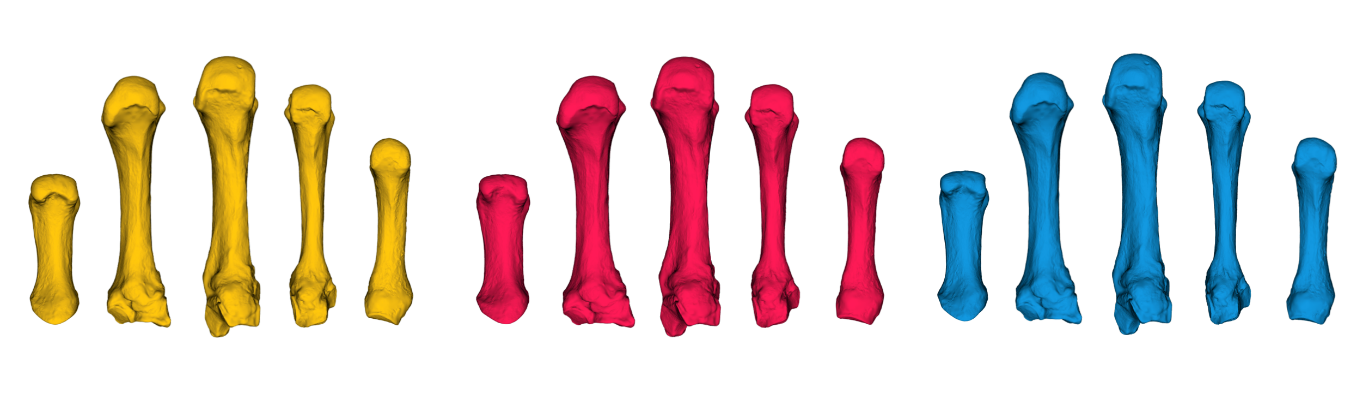
Supplementary Figure 7: Scaled warps of template specimens to group mean Procrustes shape coordinates. Yellow = Pre-industrial, Red = Mary Rose, Blue = Post-industrial

6. Size of all metacarpals

Supplementary Figure 8 displays the distribution of size, using ln (Centroid Size) and a pooled human sample across all metacarpals. MC1s are the shortest, with the MC5 being slightly larger, then with the MC2 and MC4 being of similar sizes, and the MC3 being the largest.


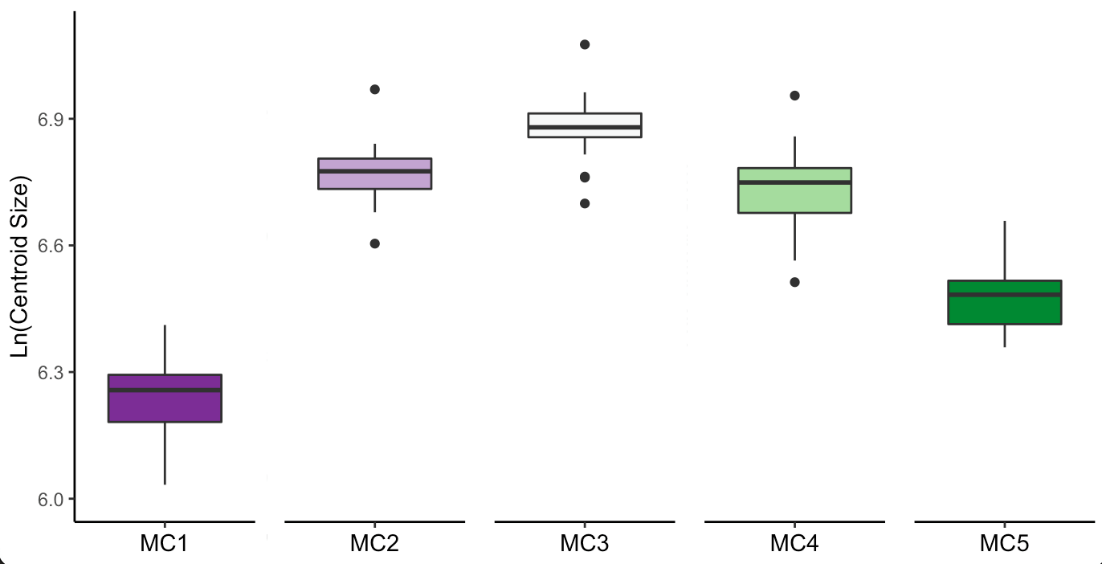


Supplementary Figure 8. Centroid size distribution across metacarpals, using a pooled human sample. ● denotes where there is an outlier that exceeds +/- 3 standard deviations of the mean.

Supplementary Figure 9 displays the distribution of size, using ln (Centroid Size), across groups and metacarpals. MC1s are the shortest, with the MC5 being slightly larger, then with the MC2 and 4 being of similar sizes and the MC3 being the largest.


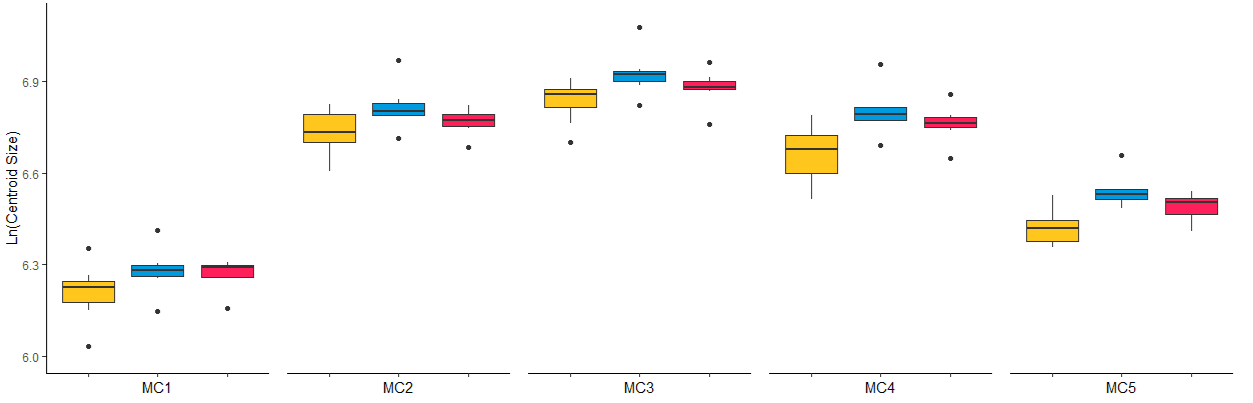


Supplementary Figure 9. Centroid size distribution across groups and metacarpals. Yellow = Pre-industrial, Blue = Post-industrial, Red = Mary Rose. ● denotes where there is an outlier that exceeds +/- 3 standard deviations of the mean.

7. Differences in terms of cross-sectional area and the polar moment of inertia between metacarpals

Wilcoxon rank sum tests were conducted on the values of *CSA* and *J* between metacarpals using a pooled human sample (Table.6). Regardless of position along the diaphysis (i.e., 33%, 50% or 66%) there is a consistent trend for the MC2 and MC3 to differ from MC4 and MC5 in CSA. MC1 differs from the MC2 and MC3 proximally and from MC4 and MC5 at the mid-diaphysis and distally in *CSA*. For *J*, there was a consistent pattern along the diaphysis for MC1, MC2 and MC3 to each differ from MC4 and MC5.

Supplementary Table 5: *J* and *CSA* differences between metacarpals in a pooled human sample, using pairwise Wilcoxon rank sum tests with a Bonferroni correction. *J* on upper right (grey), *CSA* on lower left of each section. Bold values=p≤0.05.

| Proximal Cross-Section (33%) | | | | | |
| --- | --- | --- | --- | --- | --- |
|  | MC1 | MC2 | MC3 | MC4 | MC5 |
| MC1 | - | >0.999 | >0.999 | **<0.001** | **<0.001** |
| MC2 | **<0.001** | - | >0.999 | **<0.001** | **<0.001** |
| MC3 | **<0.001** | >0.999 | - | **<0.001** | **<0.001** |
| MC4 | >0.999 | **<0.001** | **<0.001** | - | >0.999 |
| MC5 | >0.999 | **<0.001** | **<0.001** | >0.999 | - |
| Mid-Diaphyseal Cross-Section (50%) | | | | | |
|  | MC1 | MC2 | MC3 | MC4 | MC5 |
| MC1 | - | 0.400 | >0.999 | **<0.001** | **<0.001** |
| MC2 | 0.374 | - | >0.999 | **<0.001** | **<0.001** |
| MC3 | 0.136 | >0.999 | - | **<0.001** | **<0.001** |
| MC4 | **0.004** | **<0.001** | **<0.001** | - | >0.999 |
| MC5 | **0.003** | **<0.001** | **<0.001** | >0.999 | - |
| Distal Cross-Section (66%) | | | | | |
|  | MC1 | MC2 | MC3 | MC4 | MC5 |
| MC1 | - | 0.172 | >0.999 | **<0.001** | **<0.001** |
| MC2 | >0.999 | - | >0.999 | **<0.001** | **<0.001** |
| MC3 | >0.999 | >0.999 | - | **<0.001** | **<0.001** |
| MC4 | **<0.001** | **<0.001** | **<0.001** | - | >0.999 |
| MC5 | **<0.001** | **<0.001** | **<0.001** | >0.999 | - |

The below Supplementary Tables (7,8,9) showcase the differences in *J* and *CSA* between metacarpals within each sampled group. In general, the differences highlighted within the pooled human sample (Supplementary Table 6) are seen across the Mary Rose and Pre-industrial groups (Supplementary Tables 7 & 8), that the MC1-3 differ signifcantly from the MC4-5. It is the Post-industrial group, while displaying the same trends of the MC1-3 differing from the MC4-5 in Figures 9 & 10, that does not showcase the same pattern of significant differences between the MC1-3 and MC4-5 (Supplementary Table 9).

Supplementary Table 6: *J* and *CSA* differences between metacarpals in the **Mary Rose** sample, using pairwise Wilcoxon rank sum tests with a Bonferroni correction. *J* on upper right (grey), *CSA* on lower left of each section. Bold values=p≤0.05.

| Proximal Cross-Section (33%) | | | | | |
| --- | --- | --- | --- | --- | --- |
|  | MC1 | MC2 | MC3 | MC4 | MC5 |
| MC1 | - | >0.999 | 0.553 | **0.025** | **0.043** |
| MC2 | 0.051 | - | >0.999 | **0.006** | **0.012** |
| MC3 | 0.193 | >0.999 | - | **0.001** | **0.016** |
| MC4 | 0.177 | **0.006** | **0.001** | - | 0.350 |
| MC5 | >0.999 | **0.012** | **0.005** | >0.999 | - |
| Mid-Diaphyseal Cross-Section (50%) | | | | | |
|  | MC1 | MC2 | MC3 | MC4 | MC5 |
| MC1 | - | >0.999 | 0.753 | **0.025** | **0.043** |
| MC2 | >0.999 | - | >0.999 | **0.006** | **0.023** |
| MC3 | >0.999 | >0.999 | - | **0.001** | **0.030** |
| MC4 | **0.025** | **0.006** | **0.002** | - | >0.999 |
| MC5 | **0.043** | **0.012** | **0.010** | >0.999 | - |
| Distal Cross-Section (66%) | | | | | |
|  | MC1 | MC2 | MC3 | MC4 | MC5 |
| MC1 | - | 0.732 | >0.999 | **0.025** | **0.043** |
| MC2 | >0.999 | - | >0.999 | **0.012** | **0.012** |
| MC3 | >0.999 | >0.999 | - | **0.020** | **0.005** |
| MC4 | 0.177 | 0.262 | 0.330 | - | >0.999 |
| MC5 | **0.043** | **0.012** | **0.010** | >0.999 | - |

Supplementary Table 7: *J* and *CSA* differences between metacarpals in the **Pre-industrial** sample, using pairwise Wilcoxon rank sum tests with a Bonferroni correction. *J* on upper right (grey), *CSA* on lower left of each section. Bold values=p≤0.05.

| Proximal Cross-Section (33%) | | | | | |
| --- | --- | --- | --- | --- | --- |
|  | MC1 | MC2 | MC3 | MC4 | MC5 |
| MC1 | - | >0.999 | >0.999 | **<0.001** | **0.005** |
| MC2 | **0.012** | - | >0.999 | **<0.001** | **0.008** |
| MC3 | **0.040** | >0.999 | - | **<0.001** | **0.031** |
| MC4 | >0.999 | **<0.001** | **<0.001** | - | >0.999 |
| MC5 | >0.999 | **0.002** | **0.008** | >0.999 | - |
| Mid-Diaphyseal Cross-Section (50%) | | | | | |
|  | MC1 | MC2 | MC3 | MC4 | MC5 |
| MC1 | - | >0.999 | >0.999 | **<0.001** | **0.004** |
| MC2 | 0.251 | - | >0.999 | **<0.001** | **0.006** |
| MC3 | 0.770 | >0.999 | - | **0.002** | **0.012** |
| MC4 | **0.076** | **<0.001** | **<0.001** | - | >0.999 |
| MC5 | **0.074** | **<0.001** | **0.004** | >0.999 | - |
| Distal Cross-Section (66%) | | | | | |
|  | MC1 | MC2 | MC3 | MC4 | MC5 |
| MC1 | - | 0.562 | >0.999 | **0.002** | **0.001** |
| MC2 | >0.999 | - | >0.999 | **0.011** | **0.006** |
| MC3 | >0.999 | >0.999 | - | **0.010** | **0.005** |
| MC4 | **0.030** | **0.003** | **0.015** | - | >0.999 |
| MC5 | **0.008** | **<0.001** | **<0.001** | 0.986 | - |

Supplementary Table 8: *J* and *CSA* differences between metacarpals in the **Post-industrial** sample, using pairwise Wilcoxon rank sum tests with a Bonferroni correction. *J* on upper right (grey), *CSA* on lower left of each section. Bold values=p≤0.05.

| Proximal Cross-Section (33%) | | | | | |
| --- | --- | --- | --- | --- | --- |
|  | MC1 | MC2 | MC3 | MC4 | MC5 |
| MC1 | - | >0.999 | >0.999 | 0.087 | 0.173 |
| MC2 | **0.047** | - | >0.999 | 0.140 | 0.177 |
| MC3 | 0.082 | >0.999 | - | 0.140 | 0.101 |
| MC4 | >0.999 | **0.023** | **0.047** | - | >0.999 |
| MC5 | >0.999 | **0.025** | **0.025** | >0.999 | - |
| Mid-Diaphyseal Cross-Section (50%) | | | | | |
|  | MC1 | MC2 | MC3 | MC4 | MC5 |
| MC1 | - | >0.999 | >0.999 | 0.087 | 0.173 |
| MC2 | >0.999 | - | >0.999 | 0.221 | 0.303 |
| MC3 | >0.999 | >0.999 | - | 0.140 | 0.177 |
| MC4 | 0.152 | **0.012** | **0.012** | - | >0.999 |
| MC5 | 0.303 | **0.025** | **0.025** | >0.999 | - |
| Distal Cross-Section (66%) | | | | | |
|  | MC1 | MC2 | MC3 | MC4 | MC5 |
| MC1 | - | >0.999 | >0.999 | 0.152 | 0.173 |
| MC2 | >0.999 | - | >0.999 | 0.350 | 0.480 |
| MC3 | >0.999 | >0.999 | - | **0.047** | **0.051** |
| MC4 | 0.087 | 0.082 | **0.012** | - | >0.999 |
| MC5 | 0.087 | 0.101 | **0.025** | >0.999 | - |

8. Correlations of cross-sectional properties with components of shape variation.

Supplementary Table 9: Pearson’s correlations of CSG and shape (PC2). Correlations of the polar moment of inertia (*J*) and cross-sectional area (*CSA*) with the second principal component of shape for all metacarpals, at each cross-section. Bold values = p≤0.05.

| Metacarpal |  | Cross-Section |  | J | |  | CSA | |
| --- | --- | --- | --- | --- | --- | --- | --- | --- |
|  |  |  |  | r | p |  | r | p |
| MC1 |  | 33 |  | 0.334 | 0.451 |  | 0.298 | 0.606 |
|  |  | 50 |  | 0.403 | 0.234 |  | 0.514 | 0.062 |
|  |  | 66 |  | 0.437 | >0.999 |  | 0.439 | 0.158 |
| MC2 |  | 33 |  | -0.413 | 0.120 |  | -0.454 | 0.068 |
|  |  | 50 |  | -0.368 | 0.211 |  | -0.392 | 0.159 |
|  |  | 66 |  | -0.380 | 0.181 |  | -0.358 | 0.237 |
| MC3 |  | 33 |  | -0.066 | >0.999 |  | -0.190 | >0.999 |
|  |  | 50 |  | -0.066 | >0.999 |  | -0.282 | 0.487 |
|  |  | 66 |  | -0.068 | >0.999 |  | -0.250 | 0.654 |
| MC4 |  | 33 |  | 0.359 | 0.278 |  | 0.447 | 0.097 |
|  |  | 50 |  | 0.405 | 0.165 |  | 0.449 | 0.094 |
|  |  | 66 |  | 0.327 | 0.382 |  | 0.151 | >0.999 |
| MC5 |  | 33 |  | 0.014 | >0.999 |  | 0.066 | >0.999 |
|  |  | 50 |  | 0.199 | >0.999 |  | 0.282 | 0.610 |
|  |  | 66 |  | 0.261 | 0.722 |  | 0.376 | 0.253 |

9. Standardising the cross-sectional geometry data by metacarpal length.

We discuss the impact of standardising both J and CSA by metacarpal length on all statistical analyses involving J and CSA. (1) Group differences and (2) the correlations of J and CSA with the first two principal components of shape variation.

Supplementary Table 10: Differences in metacarpal length between groups. Pairwise T-tests with a Bonferroni correction, Bold = p≤0.05. MR=Mary Rose, Pre=Pre-industrial, Post=Post-industrial.

| Metacarpal | MR-Pre | MR-Post | Pre-Post |
| --- | --- | --- | --- |
| MC1 | 0.910 | >0.999 | 0.260 |
| MC2 | 0.785 | 0.573 | **0.044** |
| MC3 | 0.157 | 0.239 | **0.004** |
| MC4 | 0.100 | >0.999 | **0.014** |
| MC5 | 0.146 | 0.519 | **0.006** |

There are significant differences in metacarpal lengths between groups, but these are only between the Pre- and Post-industrial samples across MCs 2-5 (p<0.05), with the Mary Rose as an intermediate between the two. Even given the larger metacarpal sizes of the Post-industrial group compared to the Pre-industrial, there are more significant differences in J and CSA between the Mary Rose and Pre-industrial groups compared to the differences between the Pre- and Post-industrial groups. This suggests that size may not be not significantly impacting values of J and CSA across the groups, and it is more likely that other factors including variation in activity are contributing to any group differences.

9.1 Group differences in terms of J/length and CSA/length

While the trends of group differences are largely the same after standardizing by length, (i.e. The Mary Rose and Post-industrial groups do not significantly differ, and while those from the Post-industrial group are significantly different from the Pre-industrial sample at many sampled locations across the metacarpus, there are more significant differences between the Mary Rose and Pre-industrial groups) it does produce slightly different results for group differences in terms of J and CSA. Compared to the unstandardized data, the Pre- and Post-industrial groups are significantly different in terms of J/length at 5 additional sampled cross-sections (see table below), while five cross-sections lose significance in terms of group difference for CSA/length and one for the group differences in J/length loses significance. The differences in J/length between the Mary Rose and Pre-industrial groups are significant for one additional cross-section, while one cross-section loses its significant difference in terms of CSA/length. And there are no changes in significance for differences between the Mary Rose and Post-industrial groups (i.e., there remain no significant differences between the two groups).

Supplementary Table 11: Differences in Length Standardised *J* and *CSA* values between human groups across all metacarpals. Pairwise T tests with a Bonferroni correction. *J/*length on upper right (grey), *CSA/*length on lower left of each section. Bold = p≤0.05. ‘MR’, Mary Rose.

|  |  | Proximal (33%) | | |  | Mid-Diaphyseal (50%) | | |  | Distal (66%) | | |
| --- | --- | --- | --- | --- | --- | --- | --- | --- | --- | --- | --- | --- |
|  |  | MR | Pre | Post |  | MR | Pre | Post |  | MR | Pre | Post |
|  | MR | - | **0.013** | 0.620 |  | - | 0.170 | >0.999 |  | - | 0.360 | >0.999 |
| MC1 | Pre | **0.009** | - | 0.139 |  | 0.057 | - | 0.330 |  | 0.210 | - | 0.900 |
|  | Post | 0.596 | 0.106 | - |  | >0.999 | 0.173 | - |  | >0.999 | 0.850 | - |
|  |  |  |  |  |  |  |  |  |  |  |  |  |
|  |  | MR | Pre | Post |  | MR | Pre | Post |  | MR | Pre | Post |
|  | MR | - | **0.001** | 0.366 |  | - | **0.003** | 0.764 |  | - | **0.006** | 0.804 |
| MC2 | Pre | **<0.001** | - | **0.036** |  | **<0.001** | - | 0.068 |  | **0.001** | - | 0.111 |
|  | Post | 0.220 | **0.005** | - |  | >0.999 | **<0.001** | - |  | >0.999 | **0.016** | - |
|  |  |  |  |  |  |  |  |  |  |  |  |  |
|  |  | MR | Pre | Post |  | MR | Pre | Post |  | MR | Pre | Post |
|  | MR | - | **0.012** | >0.999 |  | - | **0.010** | >0.999 |  | - | **0.001** | 0.634 |
| MC3 | Pre | **0.001** | - | 0.109 |  | **0.002** | - | 0.099 |  | **0.001** | - | 0.066 |
|  | Post | 0.712 | **0.026** | - |  | >0.999 | **0.004** | - |  | >0.999 | **0.007** | - |
|  |  |  |  |  |  |  |  |  |  |  |  |  |
|  |  | MR | Pre | Post |  | MR | Pre | Post |  | MR | Pre | Post |
|  | MR | - | **0.009** | >0.999 |  | - | **0.003** | >0.999 |  | - | **<0.001** | 0.323 |
| MC4 | Pre | **0.003** | - | **0.004** |  | **0.007** | - | **0.017** |  | **0.002** | - | **0.036** |
|  | Post | >0.999 | **0.001** | - |  | >0.999 | **0.002** | - |  | 0.542 | 0.104 | - |
|  |  |  |  |  |  |  |  |  |  |  |  |  |
|  |  | MR | Pre | Post |  | MR | Pre | Post |  | MR | Pre | Post |
|  | MR | - | **0.047** | >0.999 |  | - | **0.014** | >0.999 |  | - | **0.001** | >0.999 |
| MC5 | Pre | **0.049** | - | 0.311 |  | **0.003** | - | 0.064 |  | **<0.001** | - | **0.002** |
|  | Post | >0.999 | 0.122 | - |  | >0.999 | **0.009** | - |  | >0.999 | **<0.001** | - |

9.2 Results of the correlation tests

For the correlations of J/length with PC1, the tests at the MC1.50+66 and MC4.33+50 cross-sections become significantly correlated, while the MC2.66 cross-section now becomes insignificant. For CSA/length correlations with PC1, the tests at MC2.33 and MC3.50+66 cross-sections become significantly correlated.

PC2 is not significantly correlated with J and CSA when standardised by length across any of the sampled metacarpal cross-sections, which is the same result obtained using the non-standardised CSG data.

The correlation results using the standardised CSG data are incredibly similar to those obtained using the non-standardised data with both highlighting the lack of a relationship between CSG variables and PC2, and strengthened positive correlations between CSG variables and PC1; so a strong relationship between increasing biomechanical strength of metacarpals (primarily an increase in the resistance to torsional deformation) and an increase to a form with notable ‘robust’ external characteristics (such as enlarged articular surfaces and wider diaphyses).

One noteworthy difference in these correlations with the standardised data is that the correlations between PC1 and J/length at the 50% and 66% cross-sections of the MC1 have now become significantly correlated. But it is also worth noting that the p values of these tests were previously close to significance, with p values of 0.073 and 0.064 respectively.

Supplementary Table 12: Correlations of the polar moment of inertia (*J*) and cross-sectional area (*CSA*) standardised by metacarpal length with the first principal component of shape for all metacarpals, at each cross-section, with Bonferroni corrections. Bold values = p≤0.05.

| Metacarpal |  | Cross-Section |  | J | |  | CSA | |
| --- | --- | --- | --- | --- | --- | --- | --- | --- |
|  |  |  |  | r | p |  | r | p |
| MC1 |  | 33 |  | 0.431 | 0.173 |  | 0.043 | >0.999 |
|  |  | 50 |  | **0.596** | **0.017** |  | 0.452 | 0.136 |
|  |  | 66 |  | **0.606** | **0.014** |  | 0.435 | 0.165 |
| MC2 |  | 33 |  | **0.778** | **<0.001** |  | **0.748** | **<0.001** |
|  |  | 50 |  | **0.749** | **<0.001** |  | **0.688** | **<0.001** |
|  |  | 66 |  | 0.752 | 0.245 |  | **0.545** | **0.015** |
| MC3 |  | 33 |  | **0.760** | **<0.001** |  | **0.632** | **0.002** |
|  |  | 50 |  | **0.707** | **<0.001** |  | **0.549** | **0.011** |
|  |  | 66 |  | **0.763** | **<0.001** |  | **0.521** | **0.019** |
| MC4 |  | 33 |  | **0.496** | **0.048** |  | 0.334 | 0.359 |
|  |  | 50 |  | **0.569** | **0.014** |  | 0.349 | 0.307 |
|  |  | 66 |  | **0.631** | **0.004** |  | 0.359 | 0.277 |
| MC5 |  | 33 |  | **0.844** | **<0.001** |  | **0.750** | **<0.001** |
|  |  | 50 |  | **0.725** | **<0.001** |  | **0.714** | **<0.001** |
|  |  | 66 |  | **0.707** | **<0.001** |  | **0.607** | **0.008** |

Supplementary Table 13: Correlations of the polar moment of inertia (*J*) and cross-sectional area (*CSA*) standardised by metacarpal length with the second principal component of shape for all metacarpals, at each cross-section, with Bonferroni corrections. Bold values = p≤0.05.

| Metacarpal |  | Cross-Section |  | J | |  | CSA | |
| --- | --- | --- | --- | --- | --- | --- | --- | --- |
|  |  |  |  | r | p |  | r | p |
| MC1 |  | 33 |  | 0.264 | 0.782 |  | 0.154 | >0.999 |
|  |  | 50 |  | 0.347 | 0.403 |  | 0.388 | 0.273 |
|  |  | 66 |  | 0.378 | 0.302 |  | 0.323 | 0.495 |
| MC2 |  | 33 |  | -0.355 | 0.245 |  | -0.311 | 0.389 |
|  |  | 50 |  | -0.305 | 0.414 |  | -0.226 | 0.831 |
|  |  | 66 |  | -0.315 | 0.375 |  | -0.176 | >0.999 |
| MC3 |  | 33 |  | -0.004 | >0.999 |  | -0.055 | >0.999 |
|  |  | 50 |  | 0.005 | >0.999 |  | -0.160 | >0.999 |
|  |  | 66 |  | -0.003 | >0.999 |  | -0.120 | >0.999 |
| MC4 |  | 33 |  | 0.326 | 0.386 |  | 0.390 | 0.198 |
|  |  | 50 |  | 0.376 | 0.231 |  | 0.366 | 0.258 |
|  |  | 66 |  | 0.293 | 0.523 |  | 0.052 | >0.999 |
| MC5 |  | 33 |  | -0.068 | >0.999 |  | -0.082 | >0.999 |
|  |  | 50 |  | 0.140 | >0.999 |  | 0.157 | >0.999 |
|  |  | 66 |  | 0.204 | >0.999 |  | 0.280 | 0.622 |

10. Assessment of within and between group variation in whole metacarpal shape

In each graph below the thick coloured lines detail the within group variation and the vertical lines showcase the 95% limits, while the filled in sections denote between-group variation. Across all five metacarpals we see that both within- and between group variation is largely similar, and there are no notable peculiarities in terms of within groups variation patterns.

Across metacarpals 1, 2, 4, and 5 the Pre-industrial group displays the greatest amount of within-group variation while the Mary Rose and Post-industrial groups possess less within-group variation. The MC3 is the only metacarpal where the Mary Rose group possesses the greatest amount of within-group variation.

We also see that generally, the greatest amounts of between group variation is between the Mary Rose and Pre-industrial groups (orange shaded section) which is consistent with the Pairwise permutational ANOVAs of Procrustes shape coordinates - showing that the greatest separation in terms of shape is between the Mary Rose and Post-industrial groups. Additionally we see the distances between the Mary Rose and Post-industrial group are generally the least across all metacarpals, and how at the MC3 the distribution of shape difference between the Pre and Post groups is of an elevated nature, explaining the significant difference in shape highlighted by the Pairwise permutational ANOVA.

We suggest that the similarly lesser amounts of within-group variation in both the Post-industrial and Mary Rose groups may be due to the shared or similar loading regimes of individuals within those groups, resulting in metacarpals with similar external shapes and more specifically similar levels of external robusticity; which is the largest component of variation in external shape in this human MC sample. Additionally, the potential for greater genetic similarities between individuals within these Medieval English and German Post-industrial groups may also decrease the potential variation in their metacarpal shape. Those from the Pre-industrial sample, comprised of six groups from across the globe, may be carrying out a diverse range of activities with varying frequencies and intensities, and as such variation in shape may be greater in this sample. It is also worth noting that there may be a genetic component to the greater amounts of shape variation in this Pre-industrial group as the sampled individuals hail from Australia, Canada, the Chatham Islands, Egypt, Greenland, and Tierra
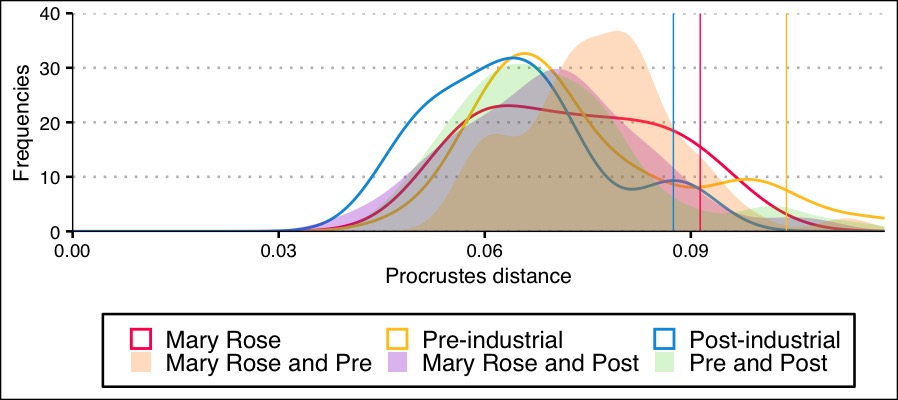
del Fuego.

Supplementary Figure 10: Within and between group variation in first metacarpal shape. Coloured lines represent within group variation, and the vertical lines show the 95% limits. Shaded areas denote
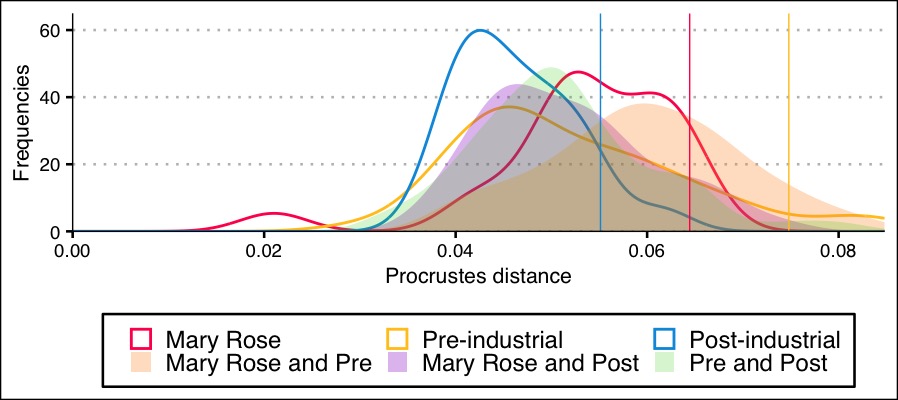
group variation between two groups - see key.

Supplementary Figure 11: Within and between group variation in second metacarpal shape. Coloured lines represent within group variation, and the vertical lines show the 95% limits. Shaded areas denote group variation between two groups - see key.

Supplementary Figure 12: Within and between group variation in third metacarpal shape. Coloured lines represent within group variation, and the vertical lines show the 95% limits.
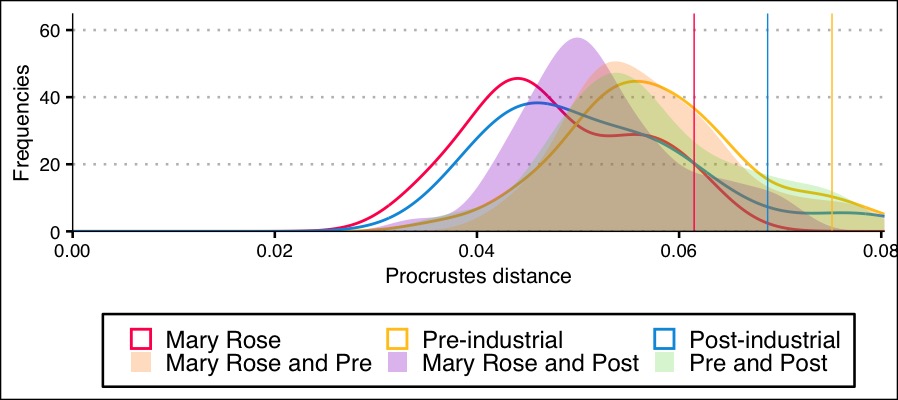
Shaded areas denote group variation between two groups - see key.

Su
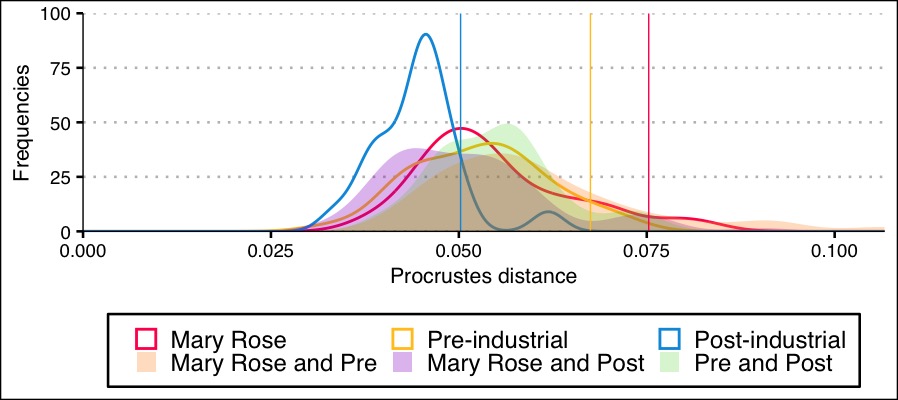
pplementary Figure 13: Within and between group variation in fourth metacarpal shape. Coloured lines represent within group variation, and the vertical lines show the 95% limits. Shaded areas denote group variation between two groups - see key.

Supplementary Figure 14: Within and between group variation in fifth metacarpal shape. Coloured lines represent within group variation, and the vertical lines show the 95% limits.
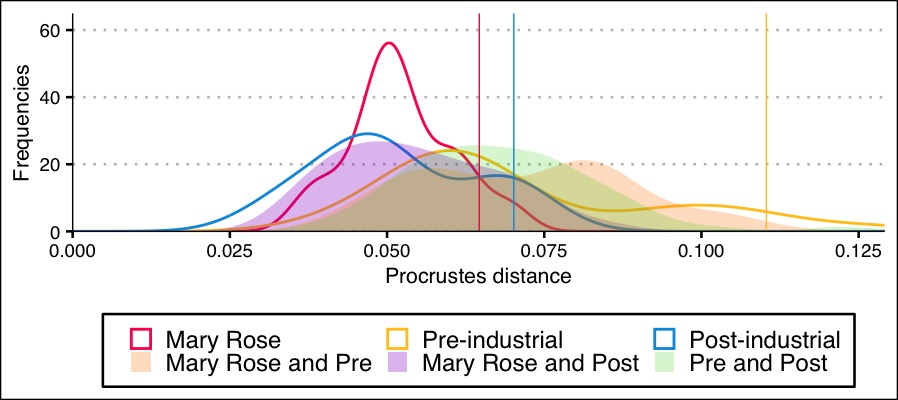
 Shaded areas denote group variation between two groups - see key.

11. Atlas of all landmarked specimens

The next five sections of the supplementary (9.1-9.5) showcases an atlas of all the landmarked metacarpals used for this study. All landmarks are yellow, with main landmarks being observably larger than the curve landmarks. Relevant aspects of proximal and distal articular surfaces are imaged. These Atlases serve as references of the landmarking conducted for this study, to ensure that readers and/or reviewers are satisfied with all the landmarking carried out by ST. Additionally, these images allow for visualisation of the variation of each specimen at the proximal and distal ends of the metacarpals.

It was decided that the facets for the MC4 on the MC3 and the facet for the MC3 on the MC4 would not be landmarked due to the large amount of variation seen with these facets– which would make the landmarking of homologous anatomical fixed landmarks unreliable.

11.1 Atlas of all landmarked first metacarpals (n=20)


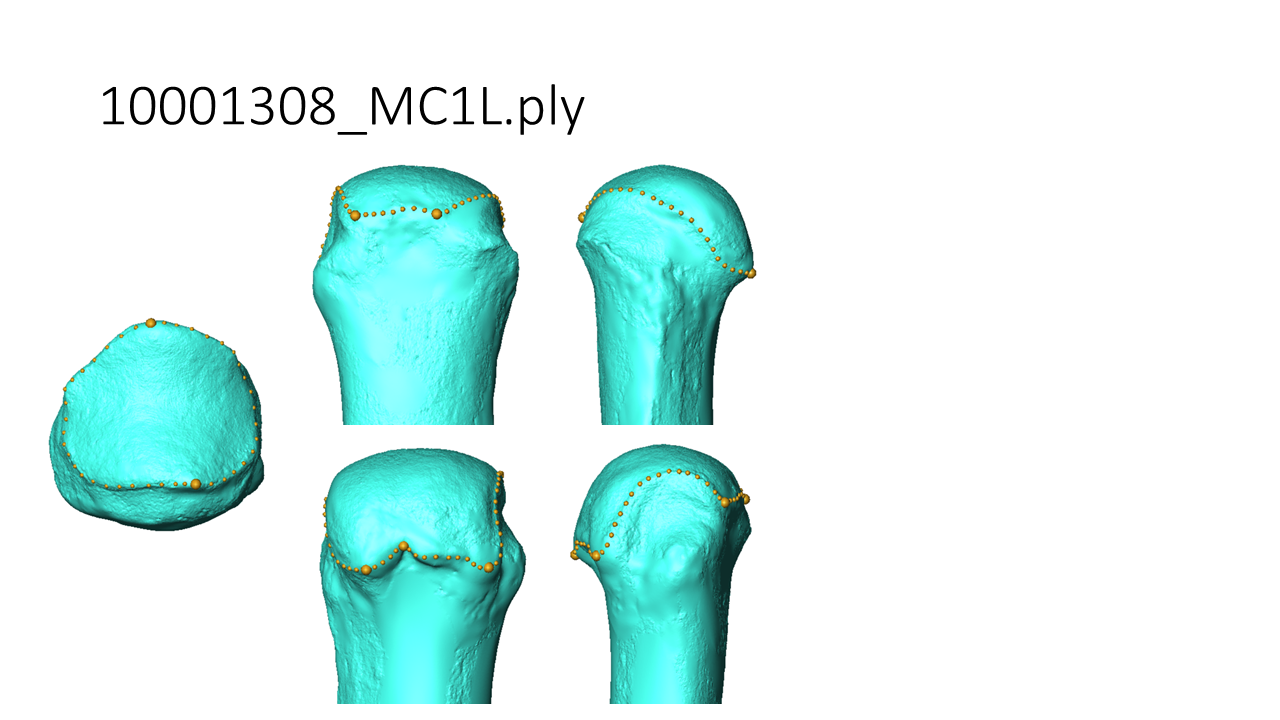

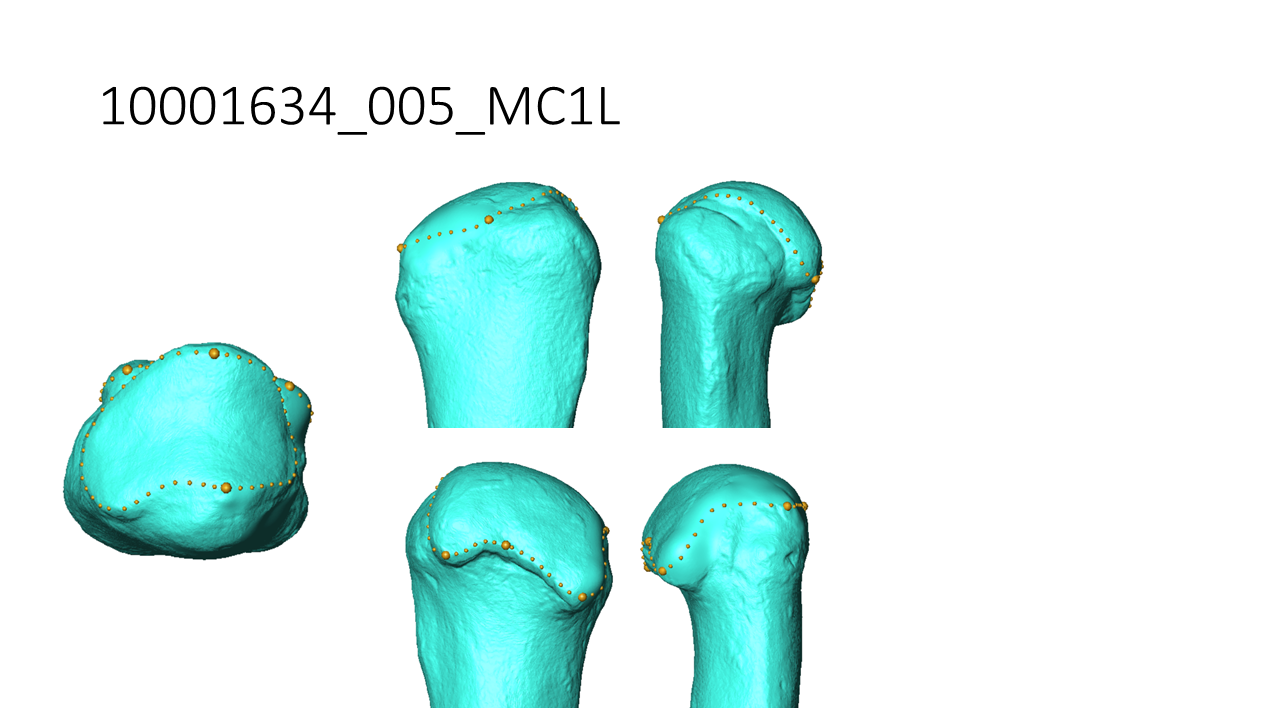

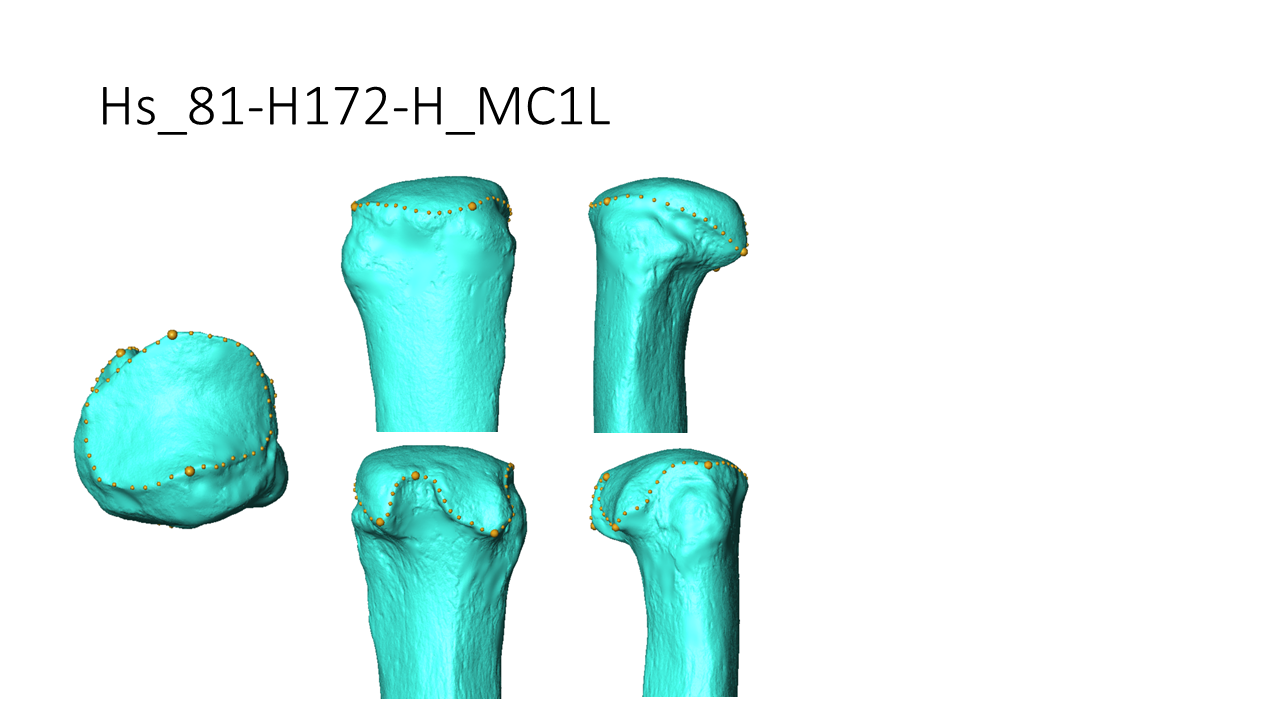

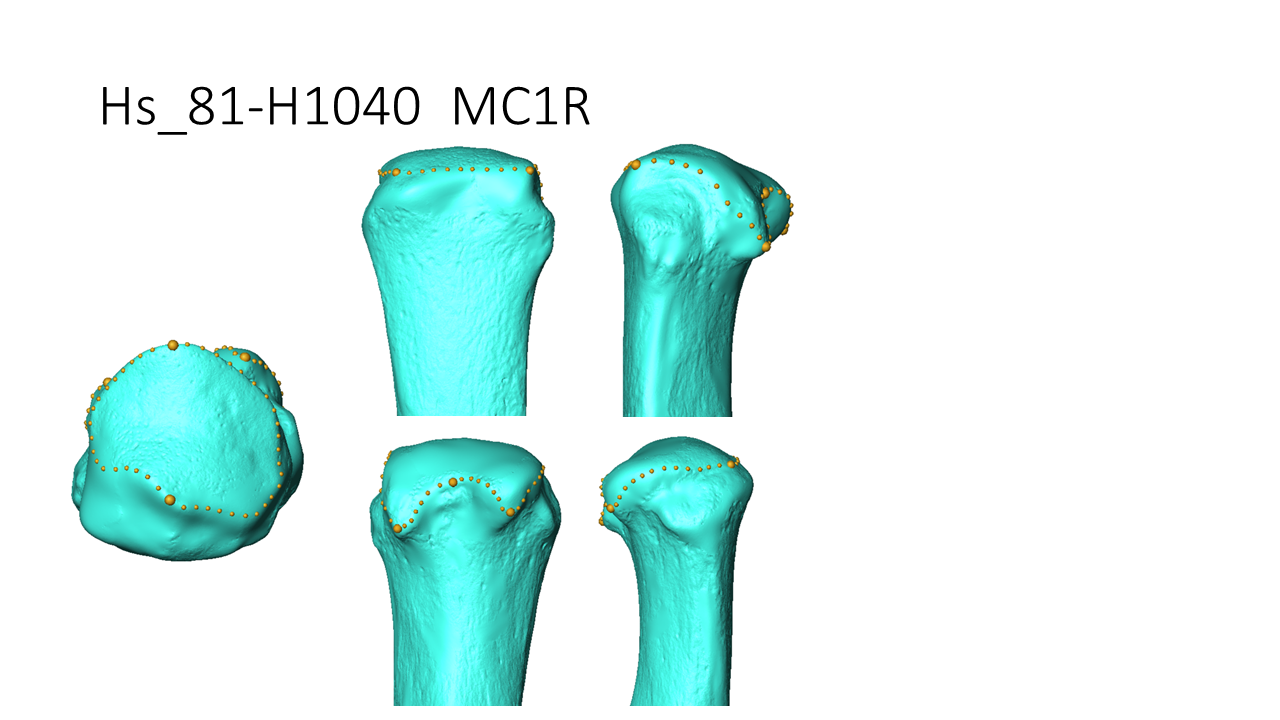

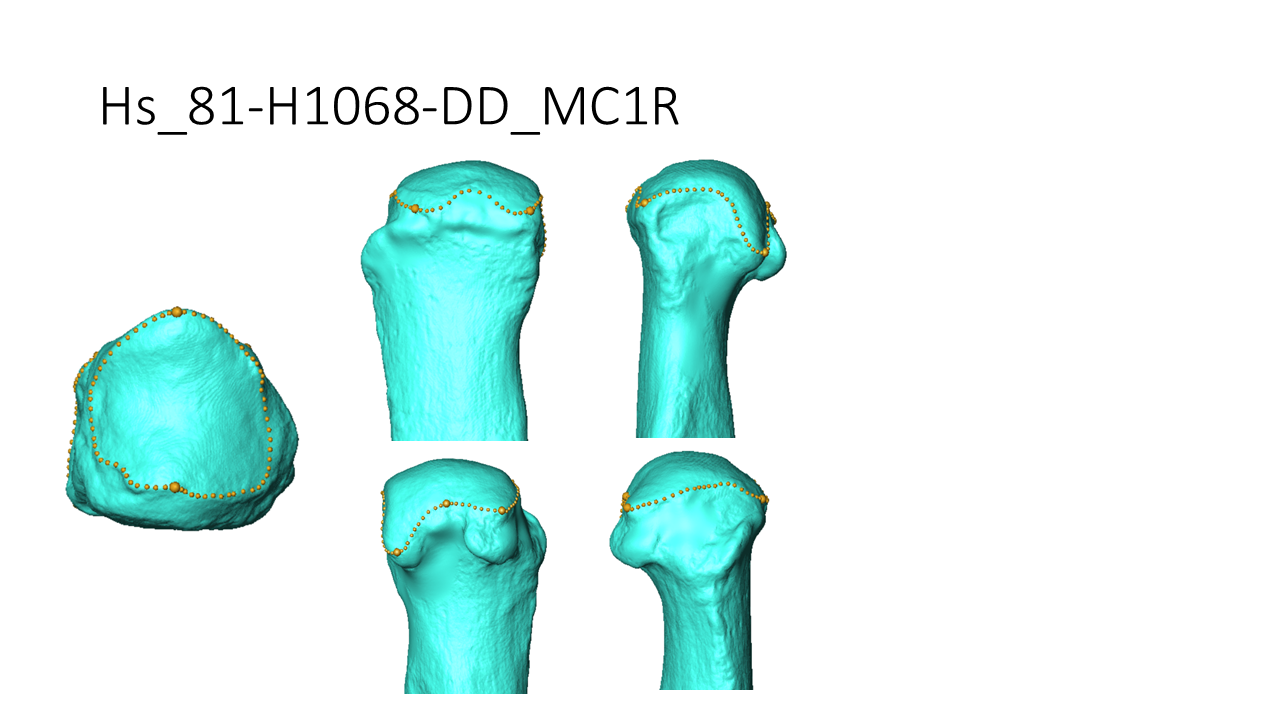

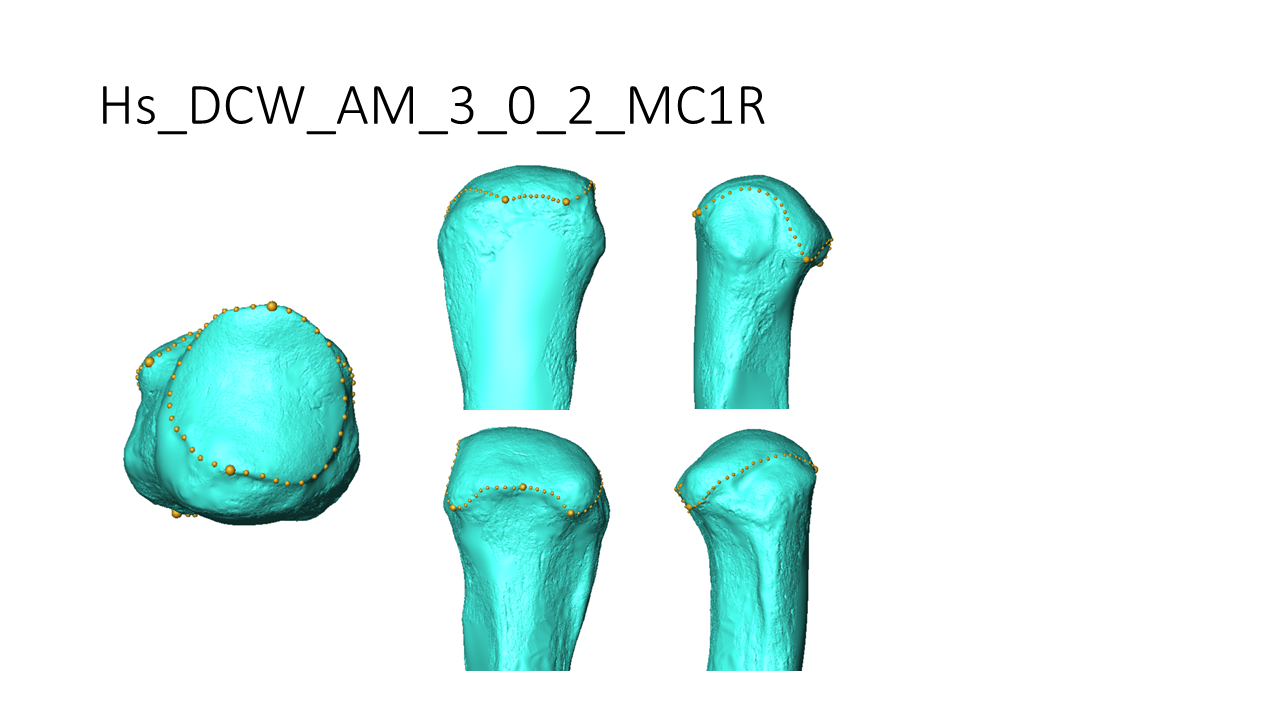

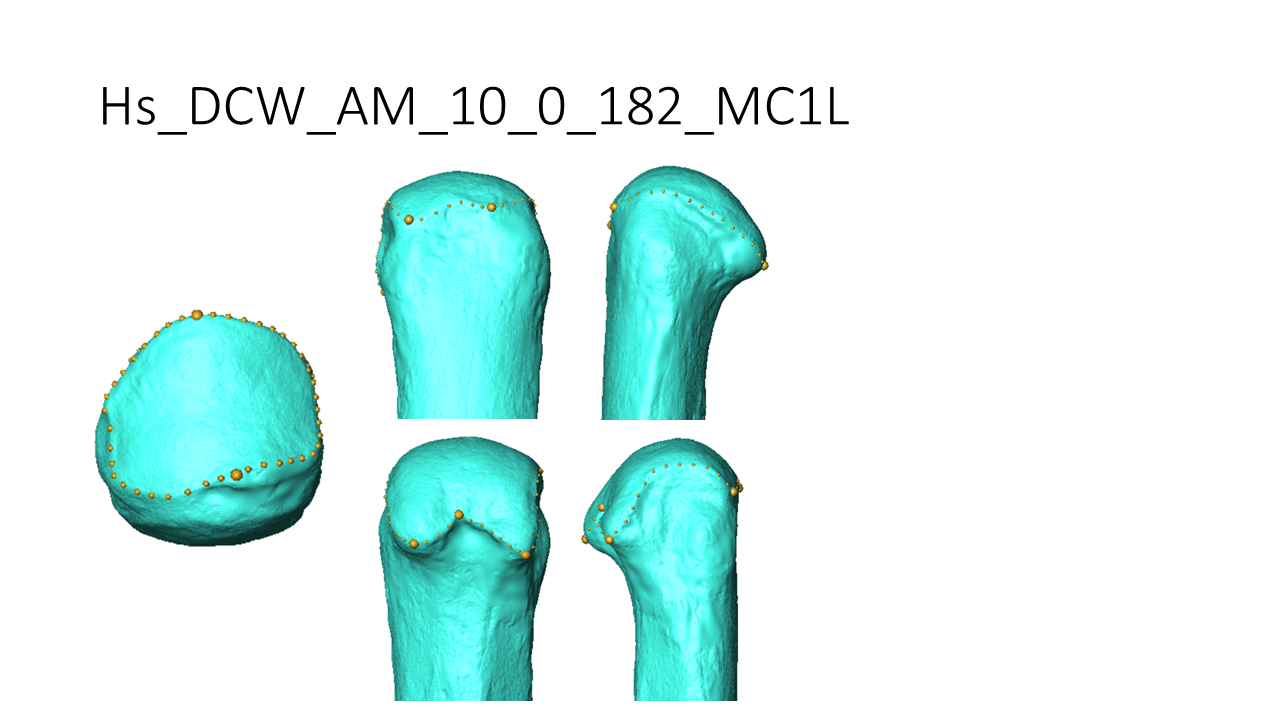

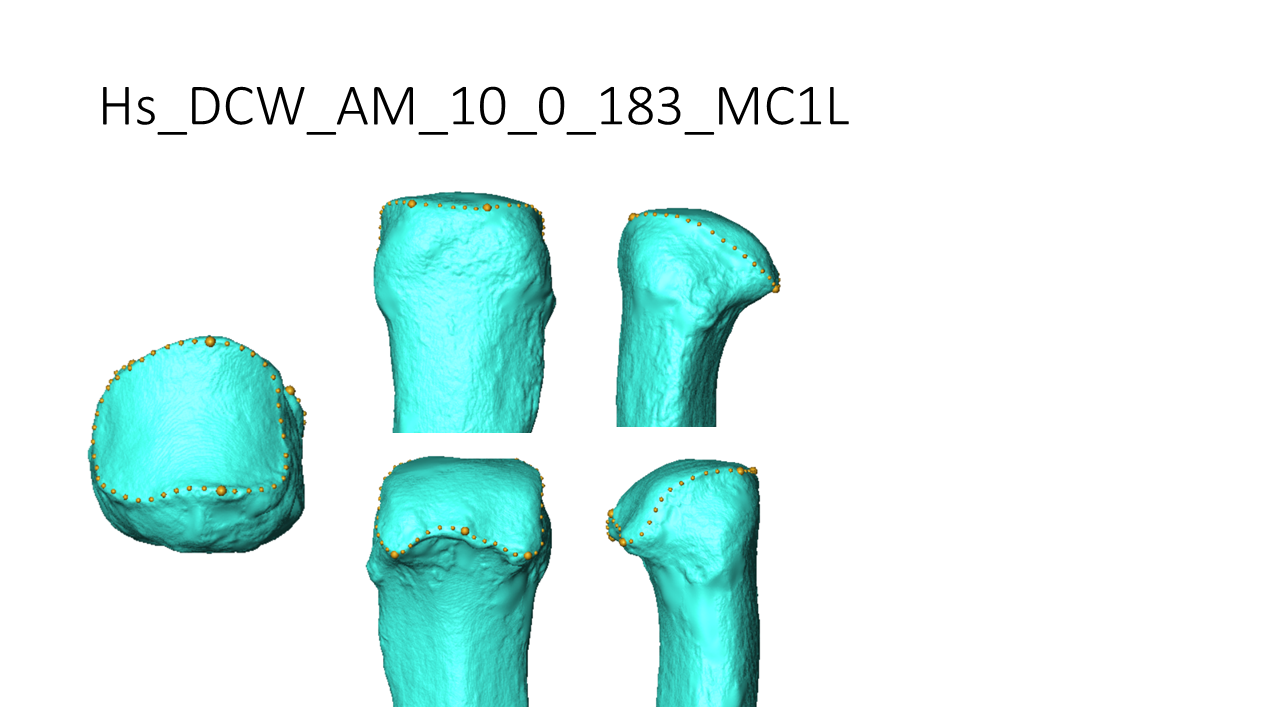

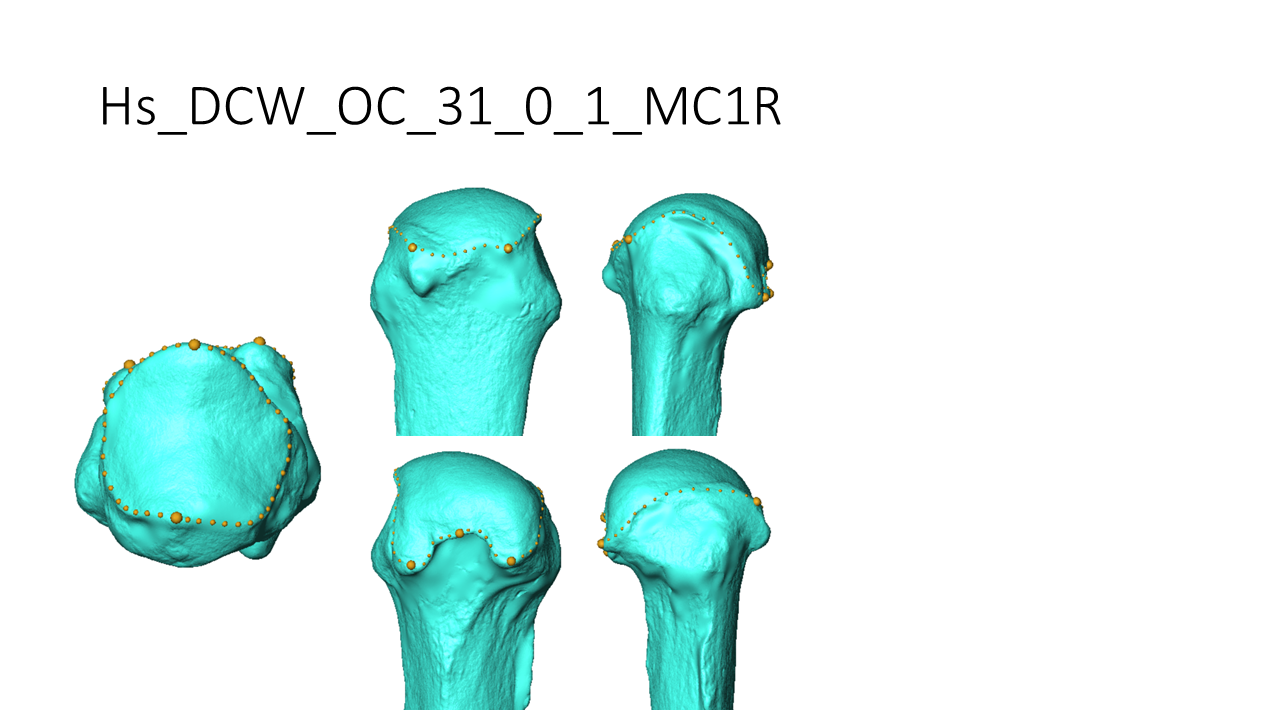

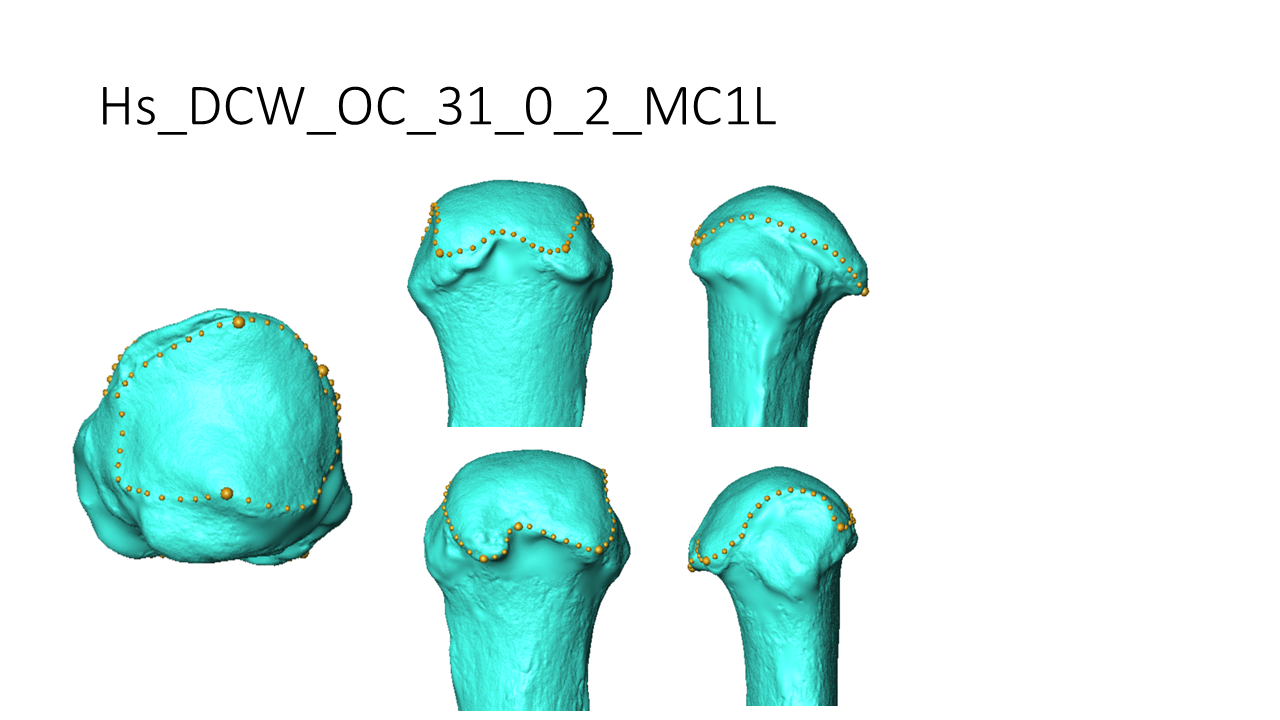

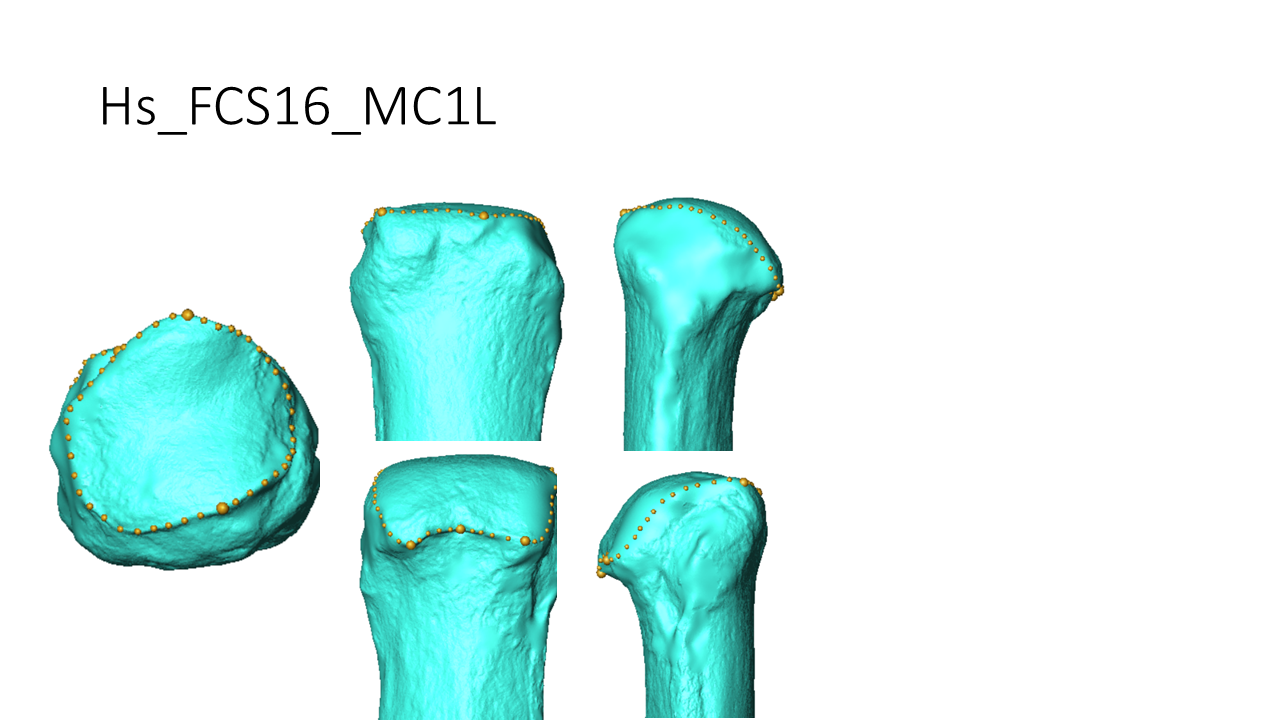

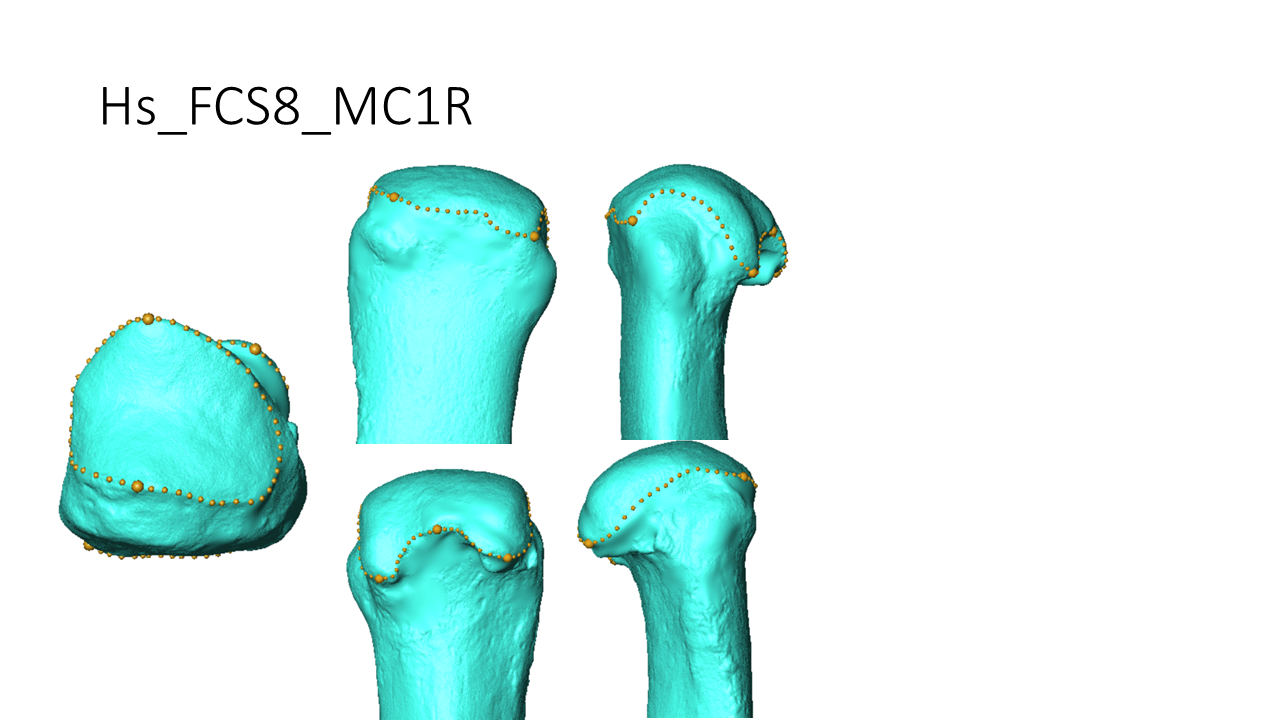

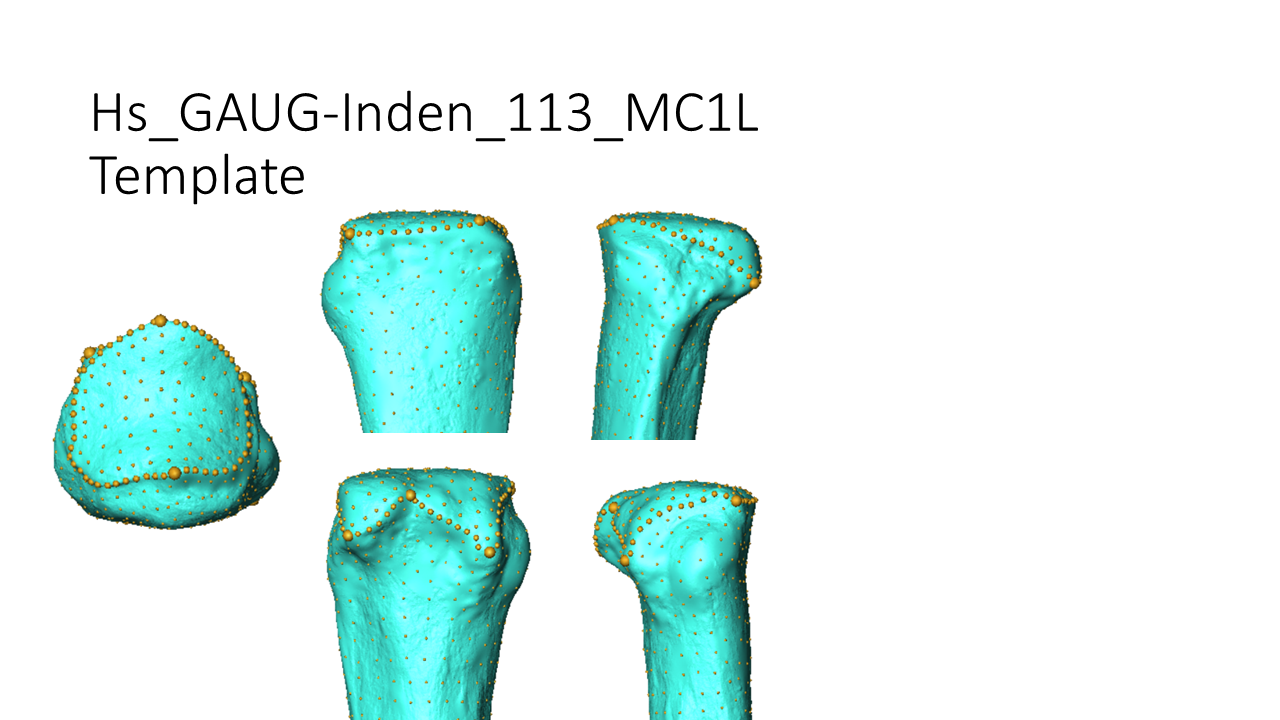

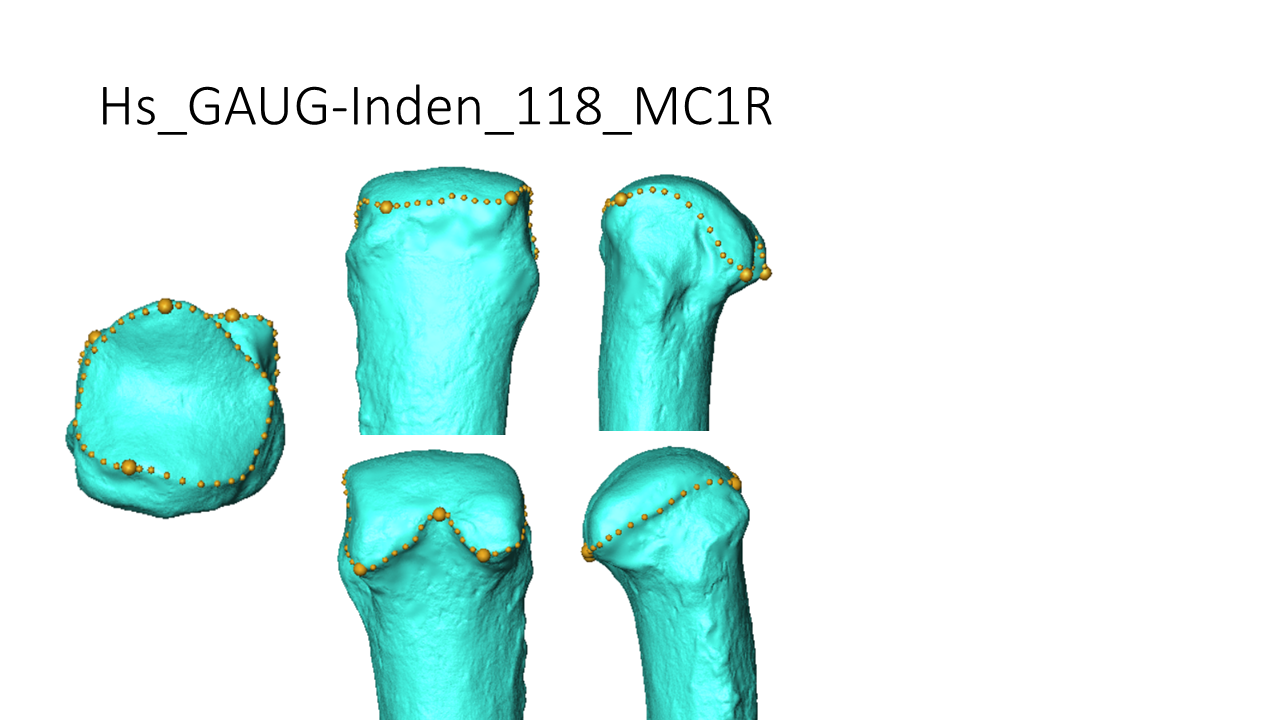


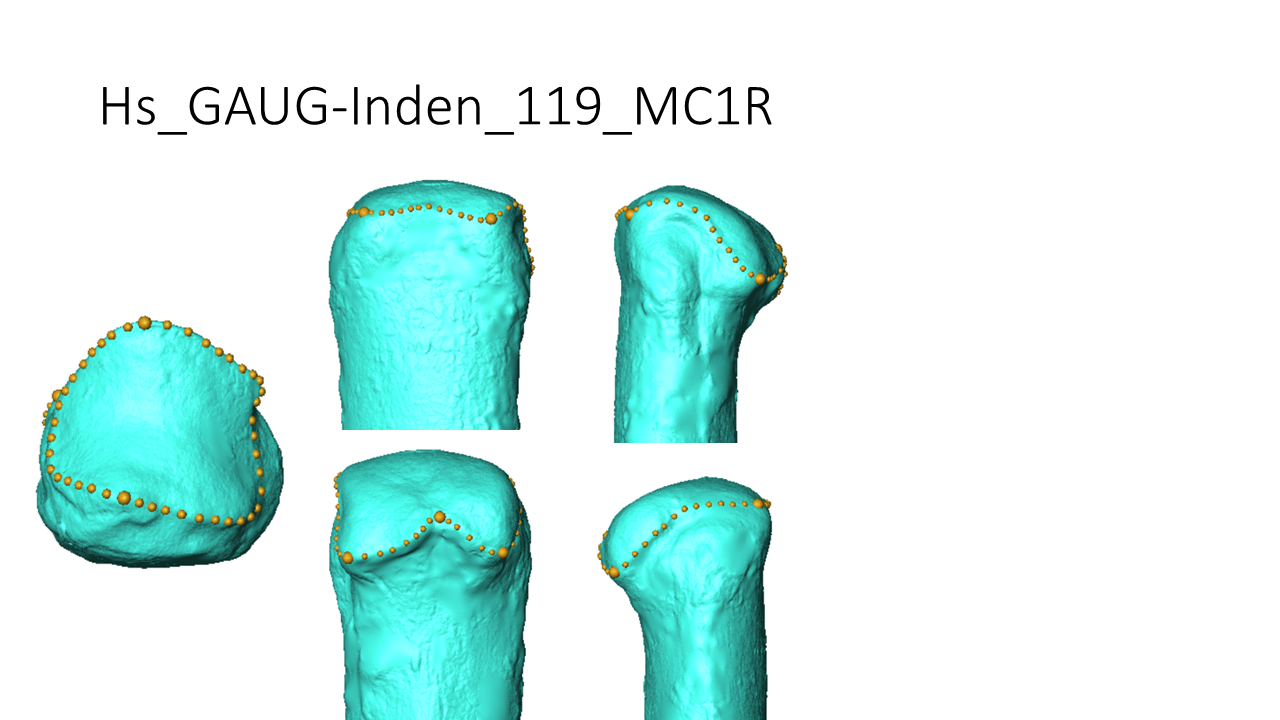


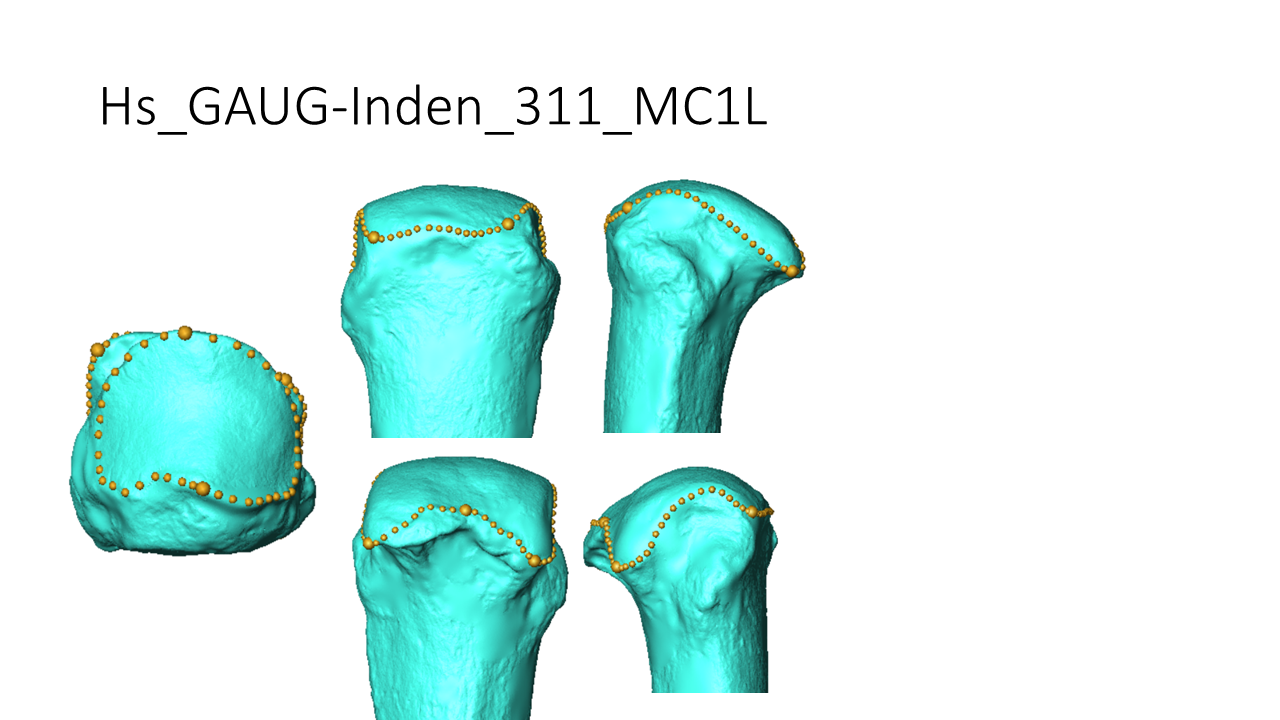


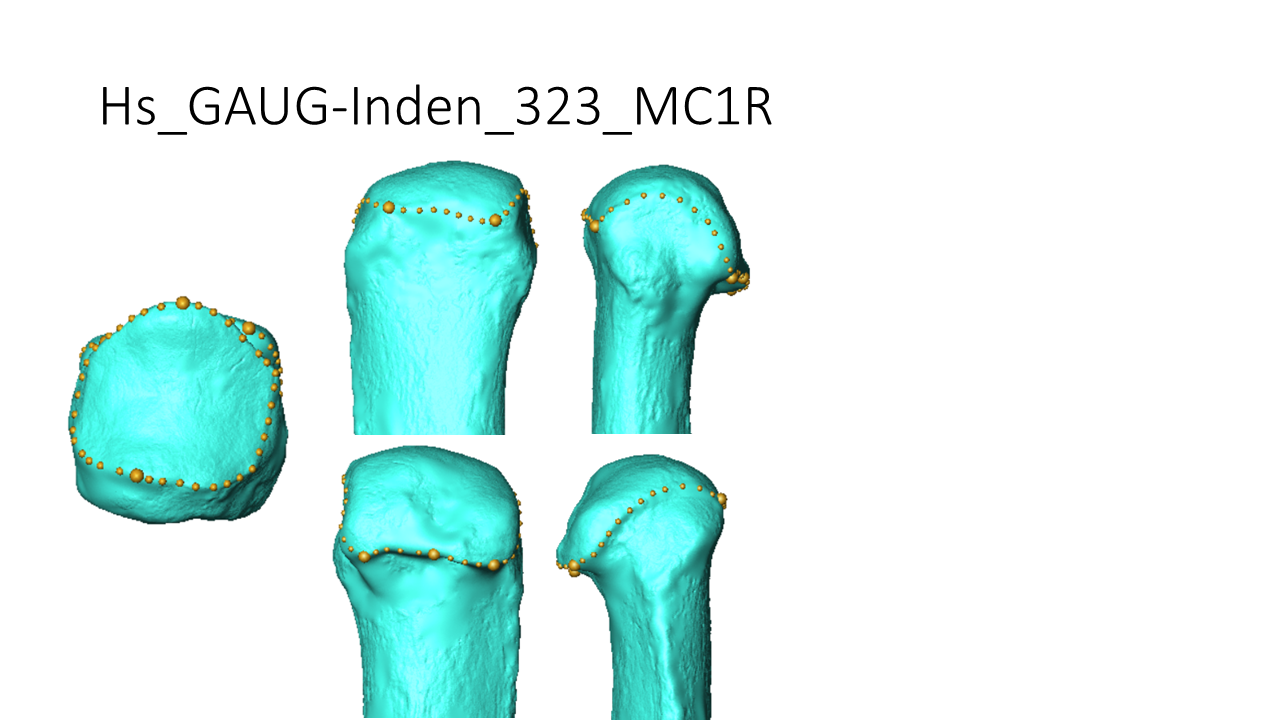


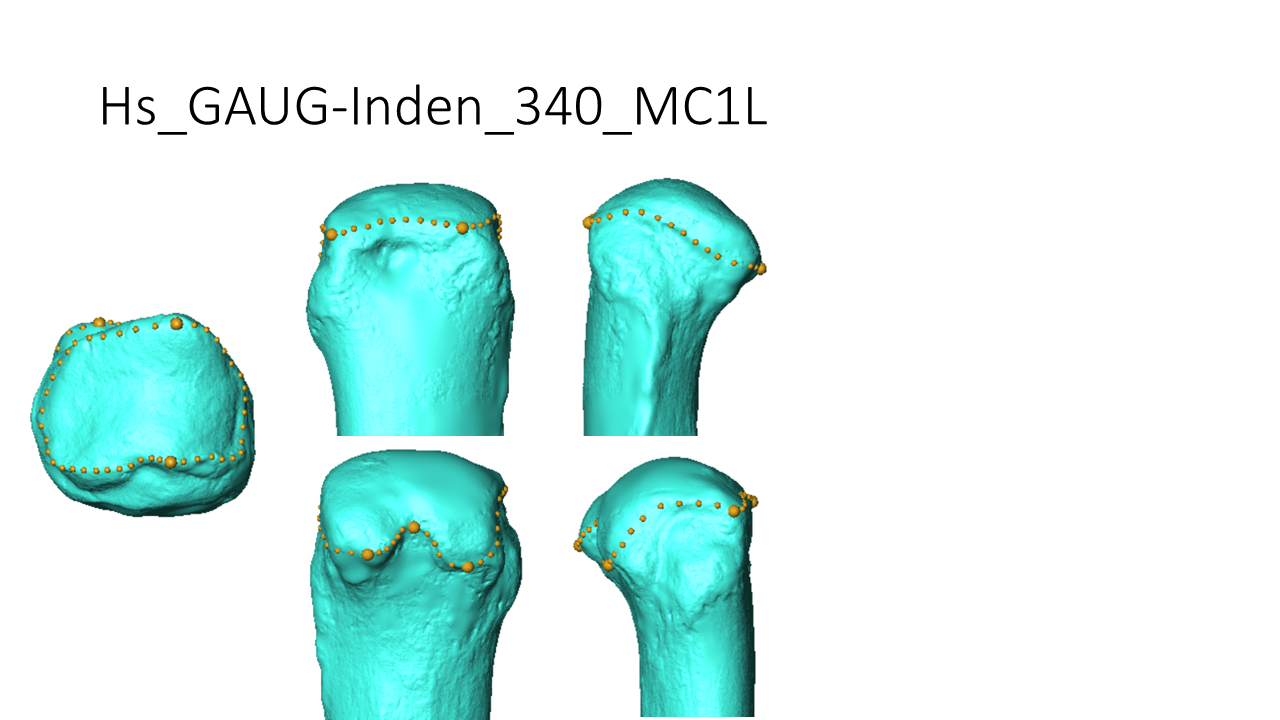


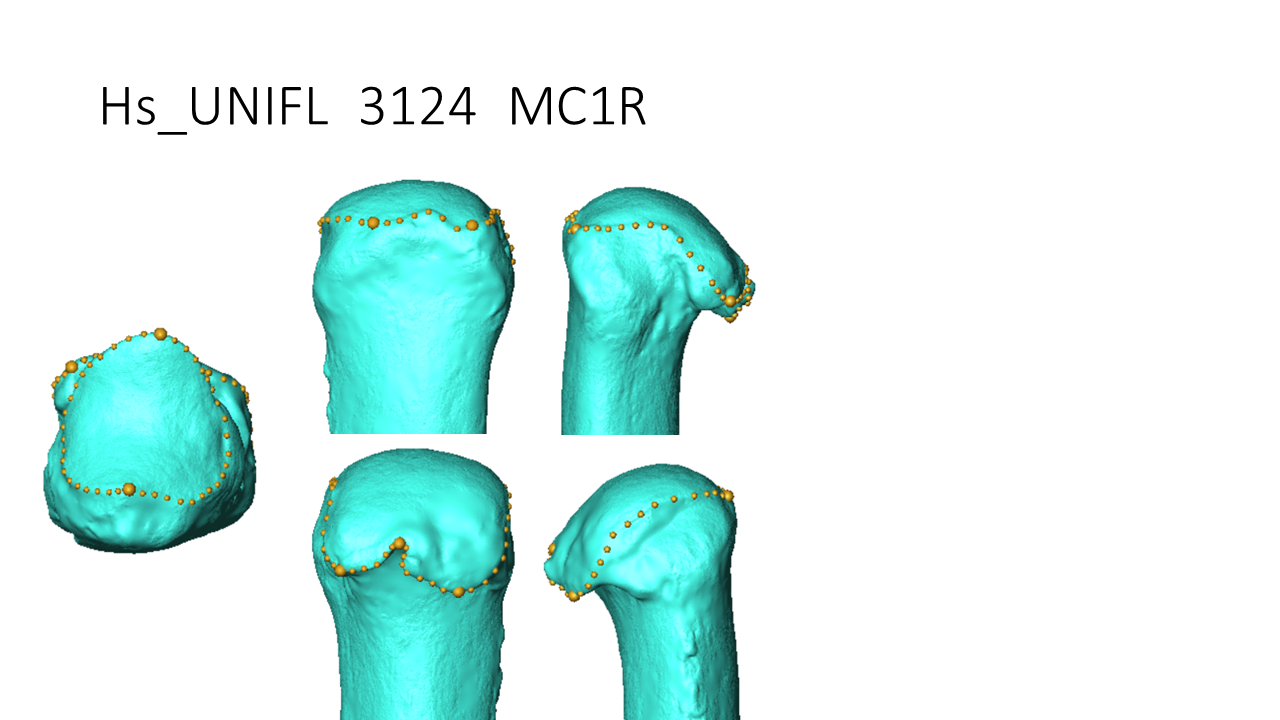


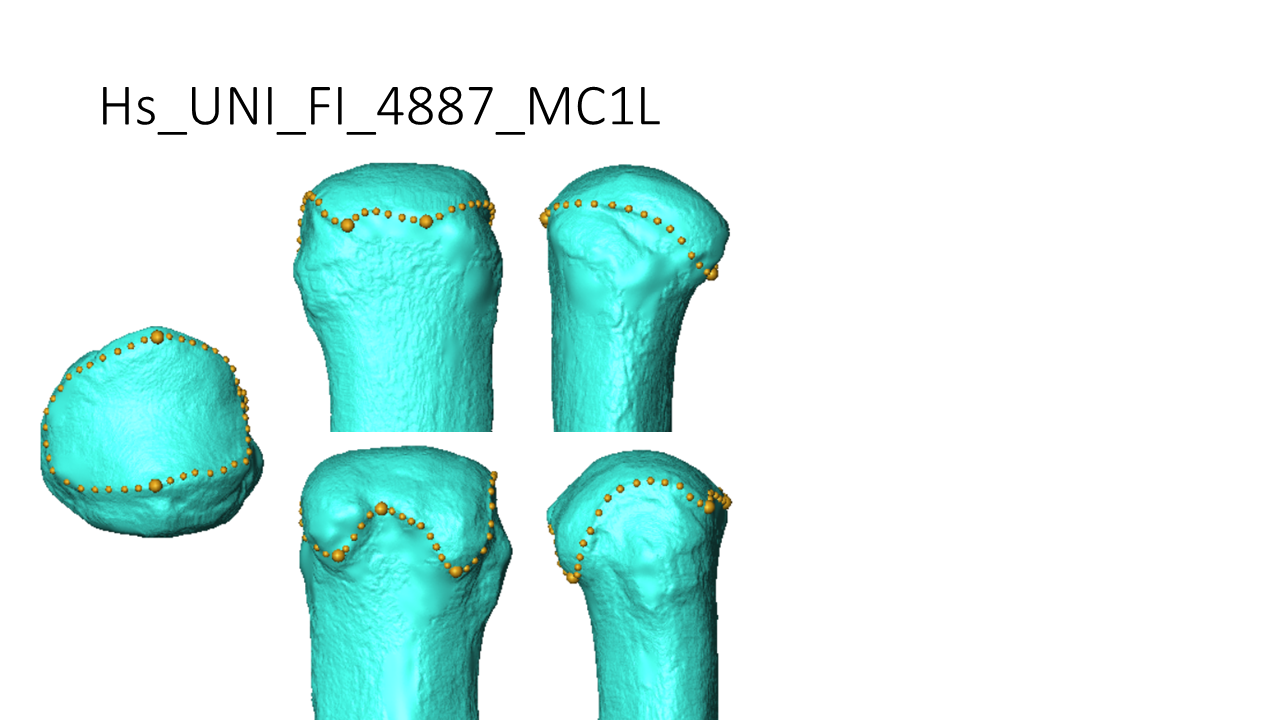


11.2. Atlas of all landmarked second metacarpals (n=25)


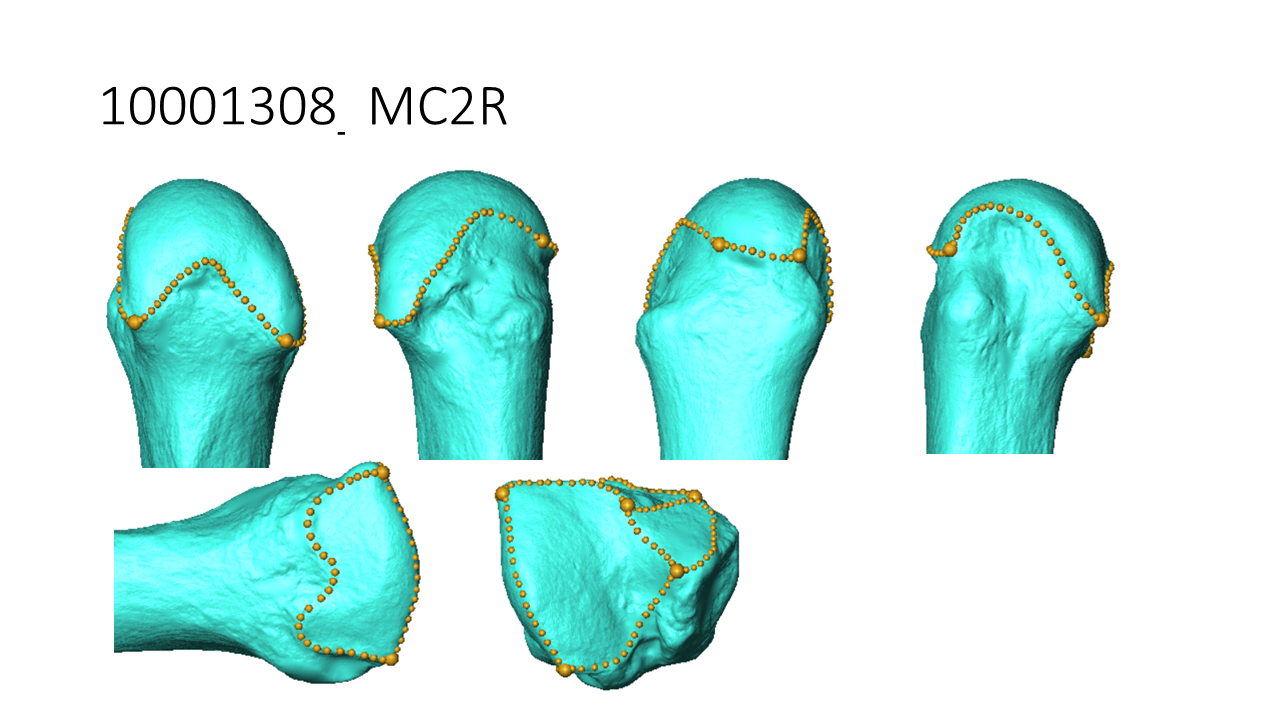

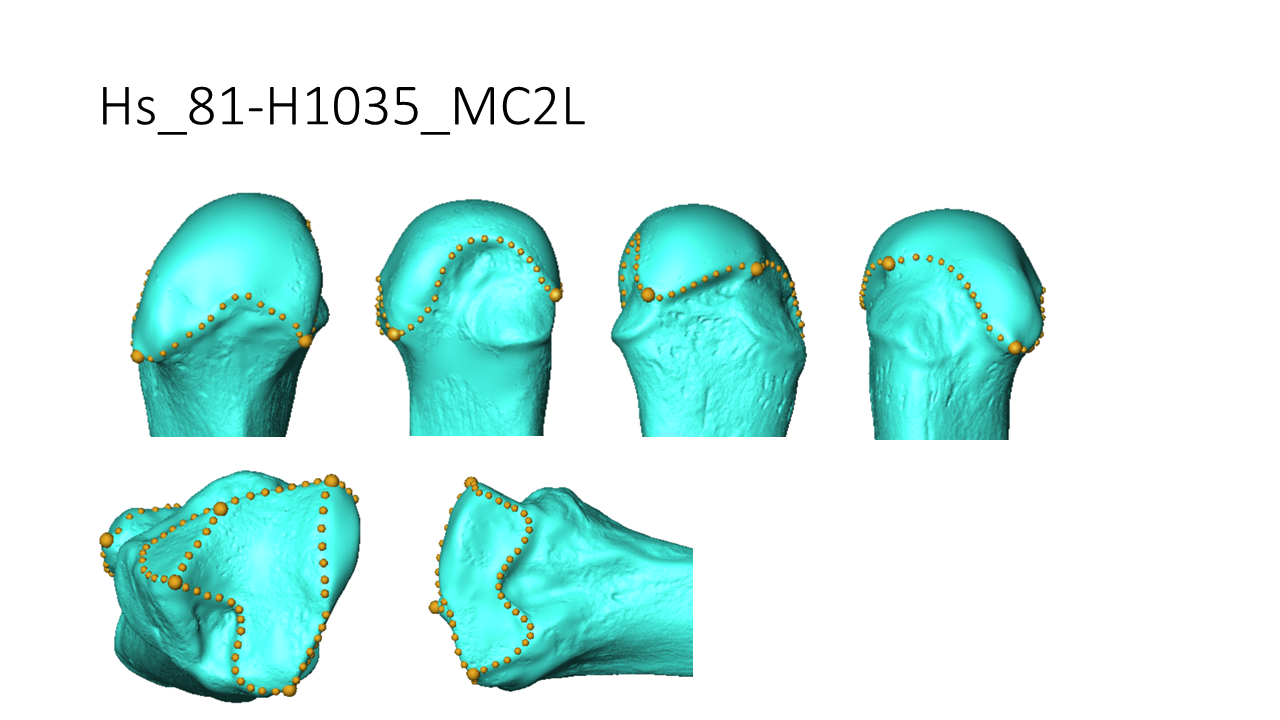


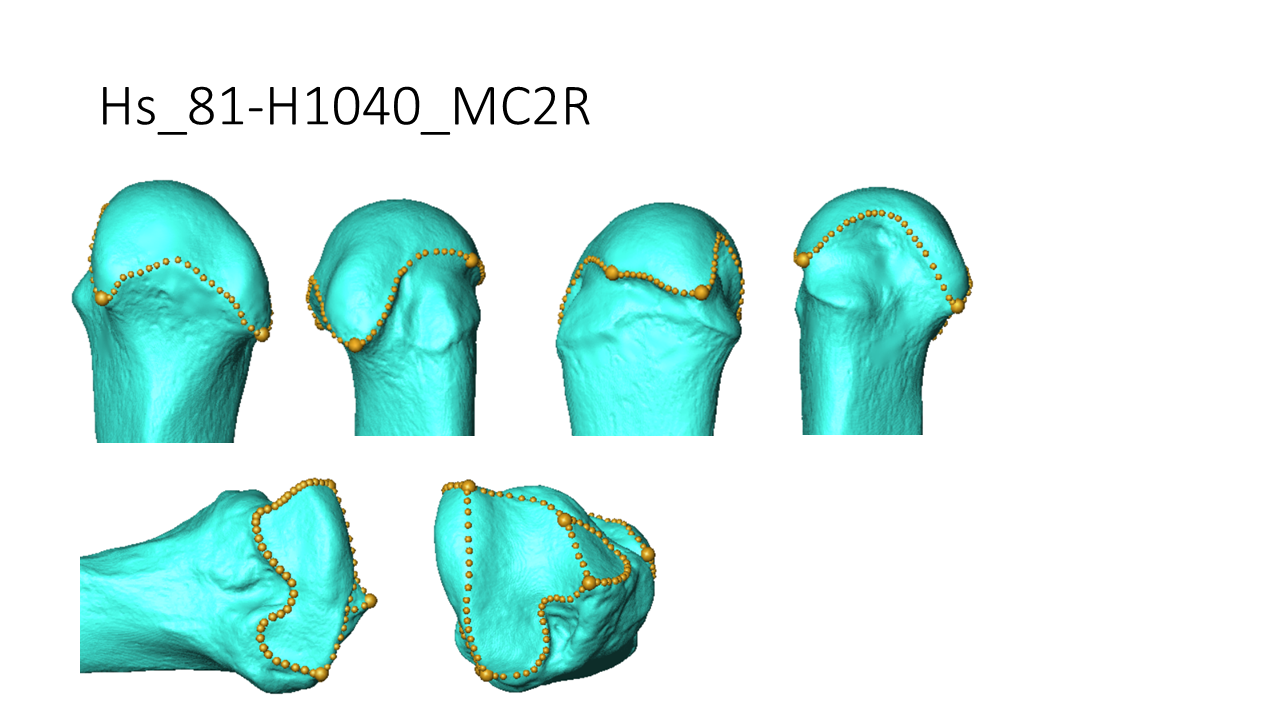


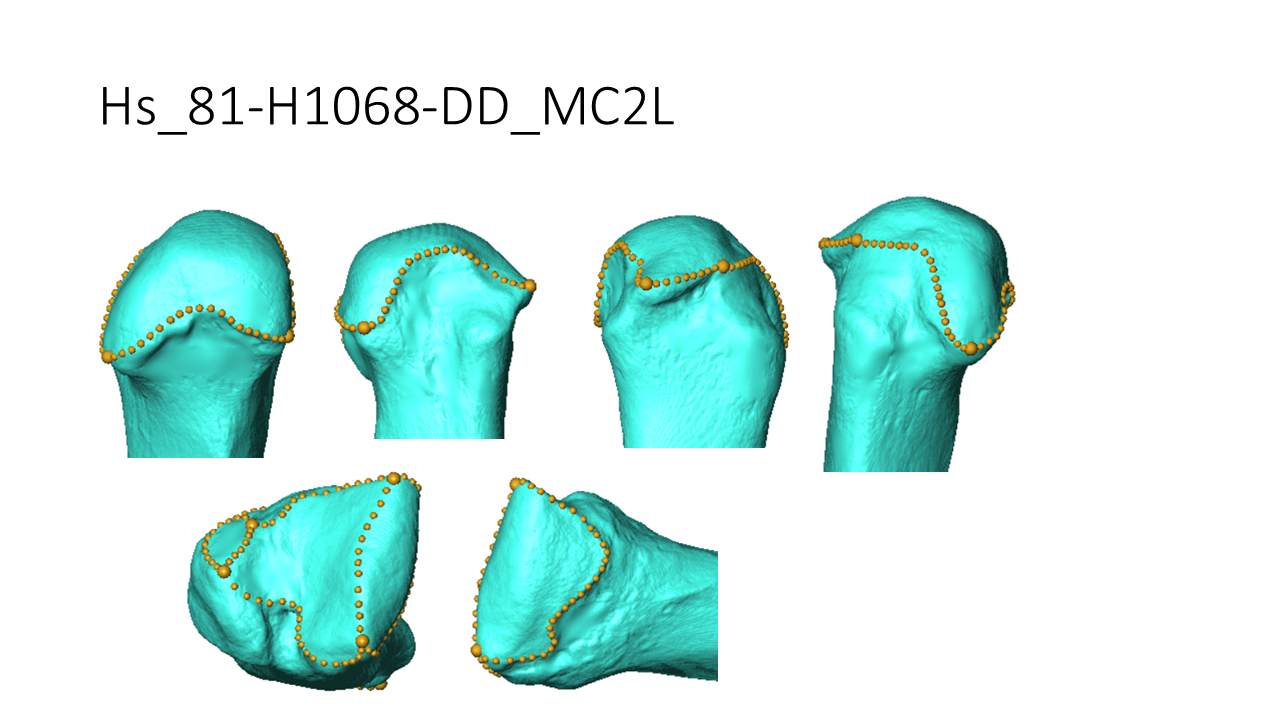


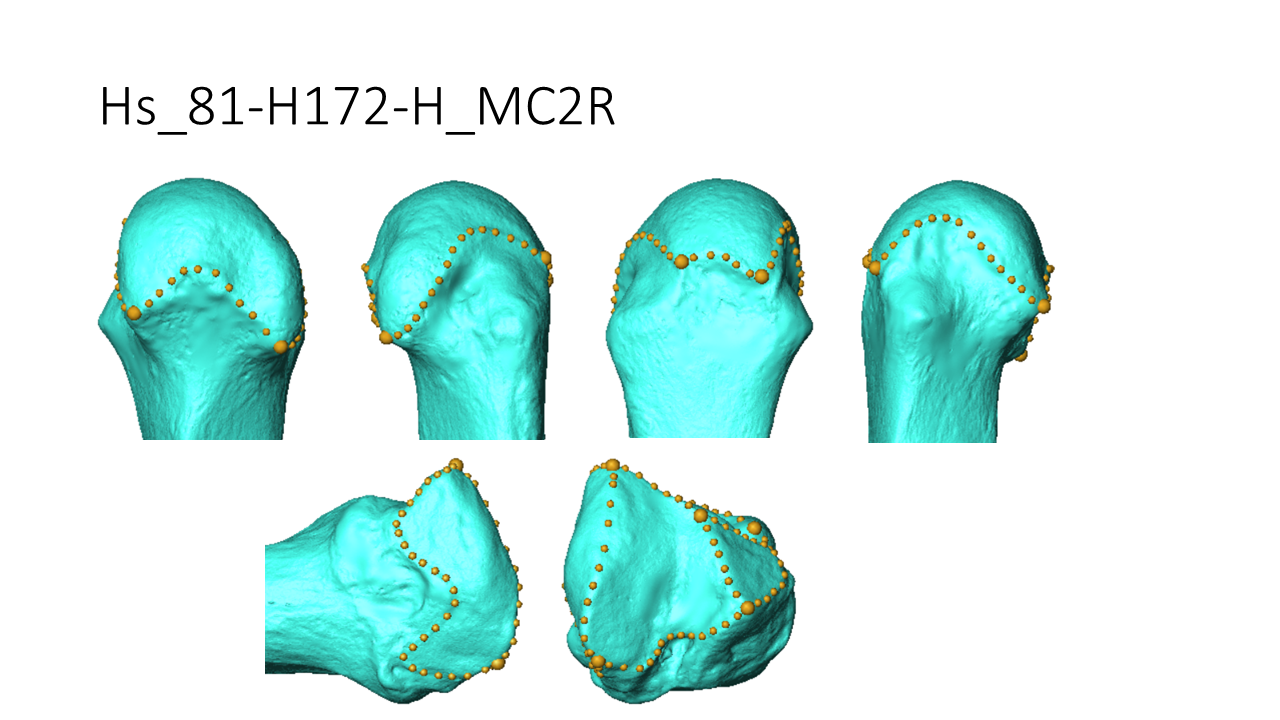


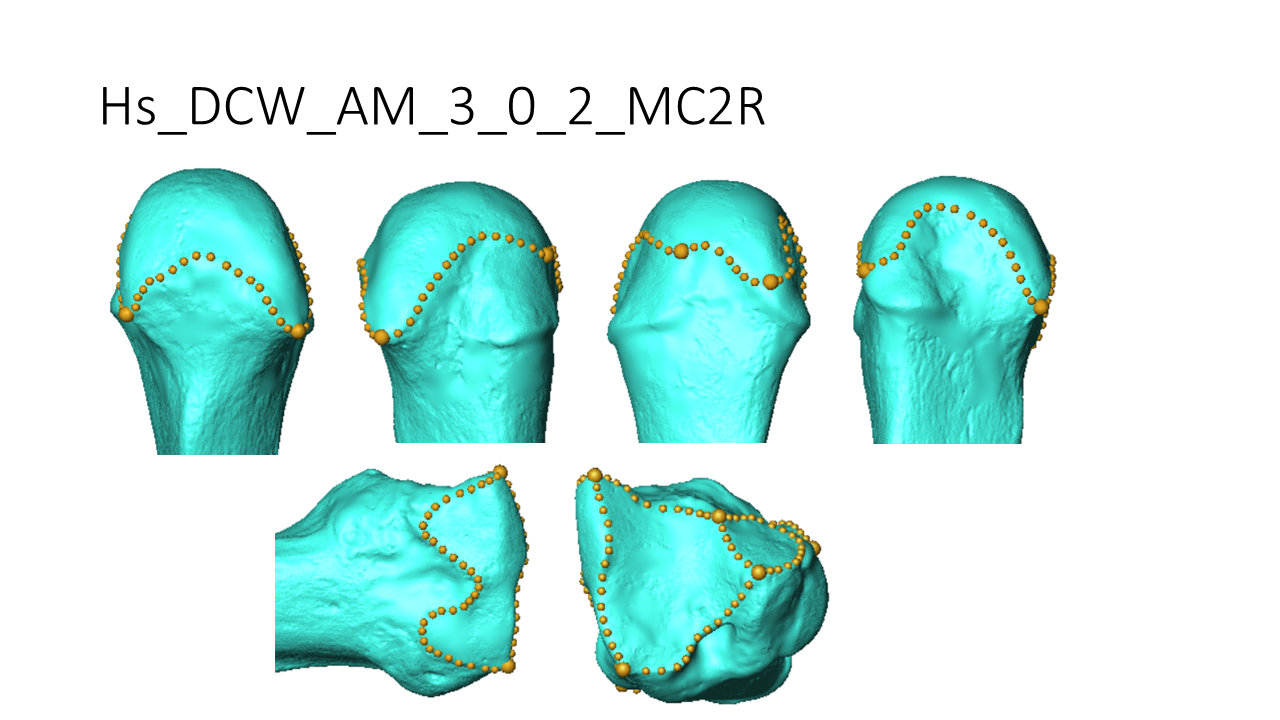


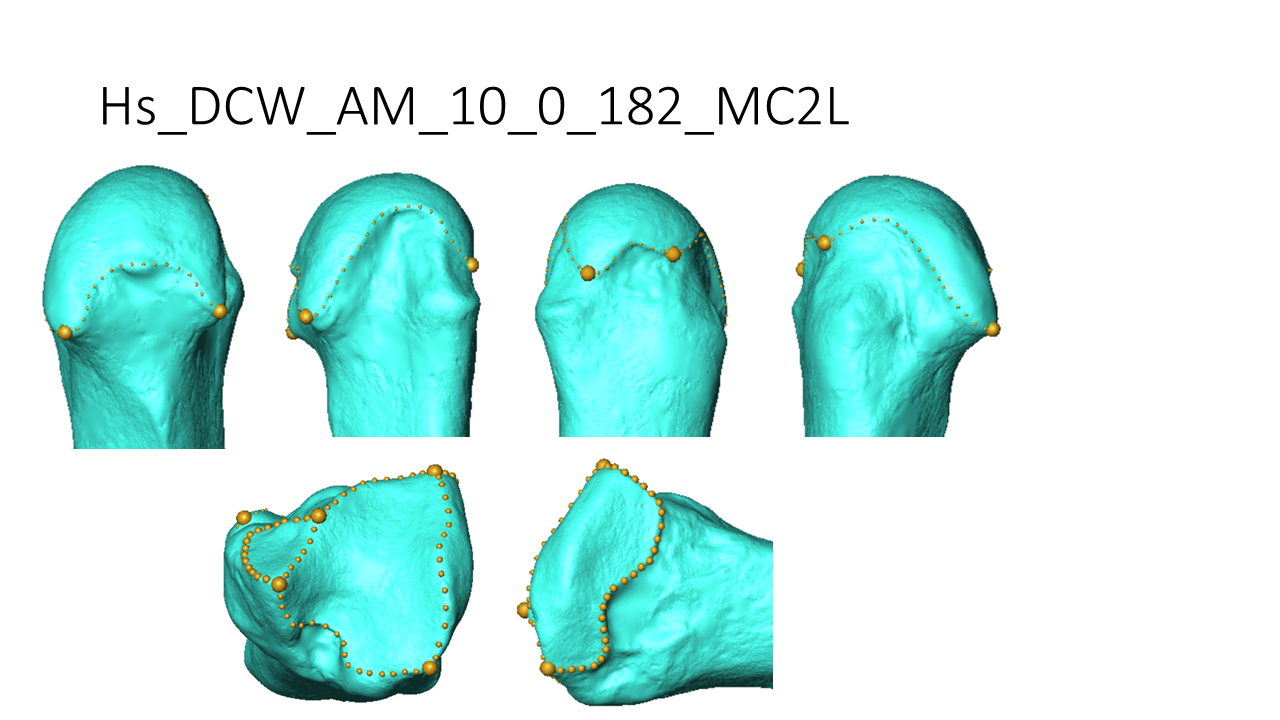


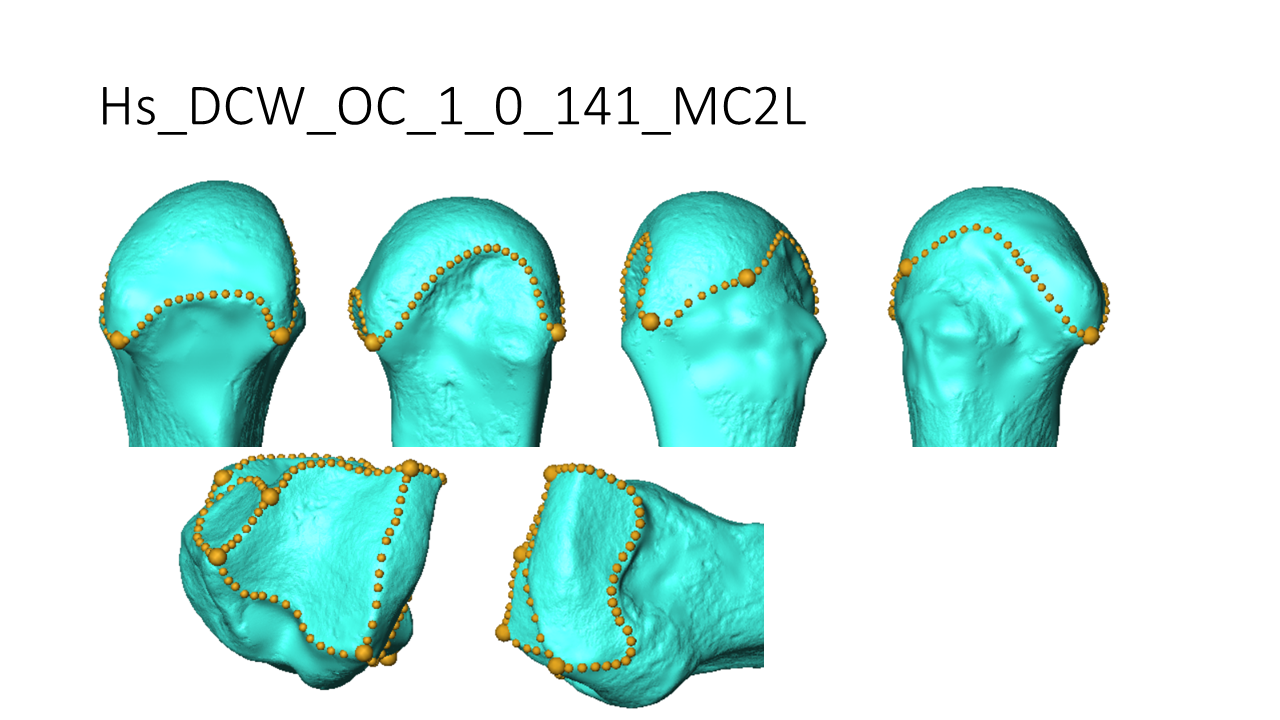


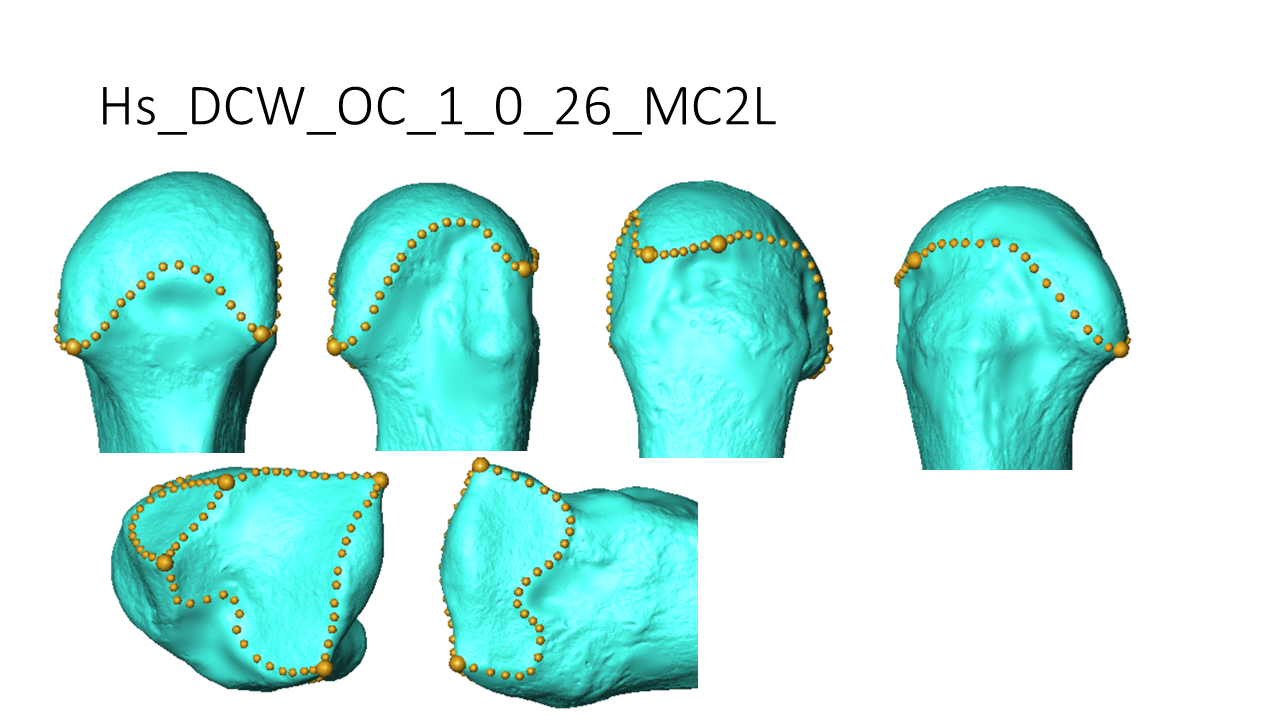


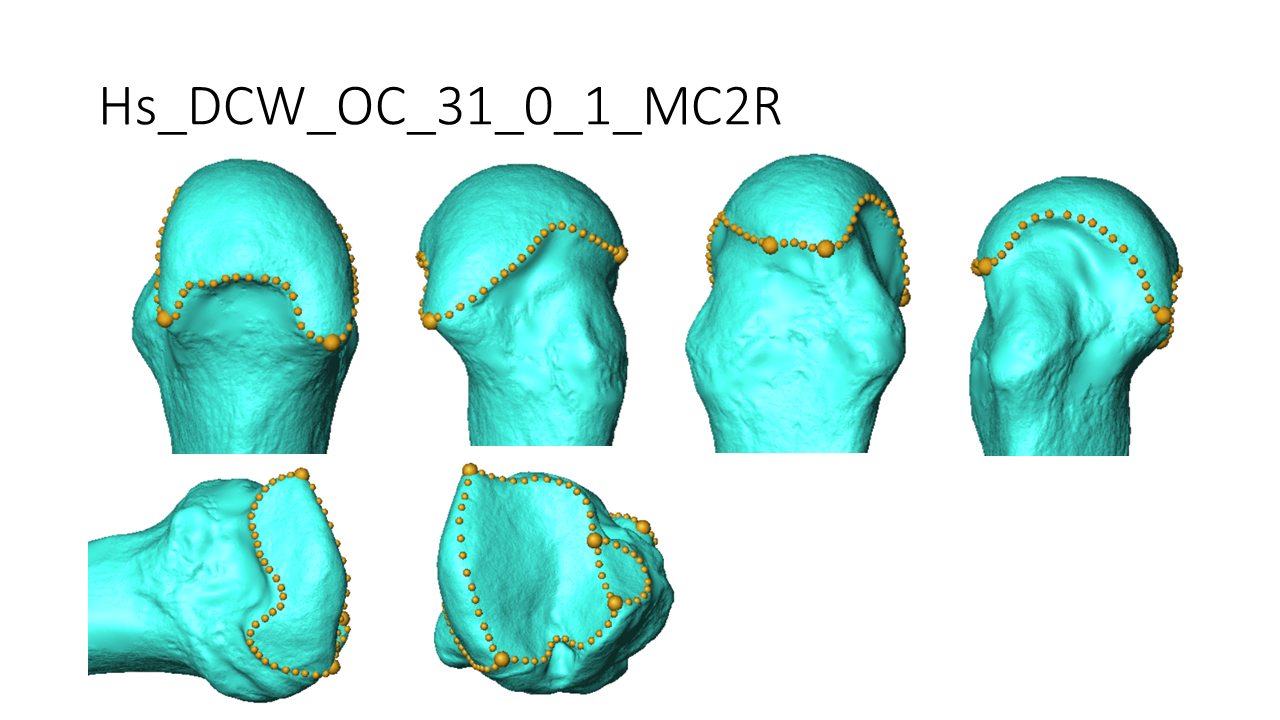


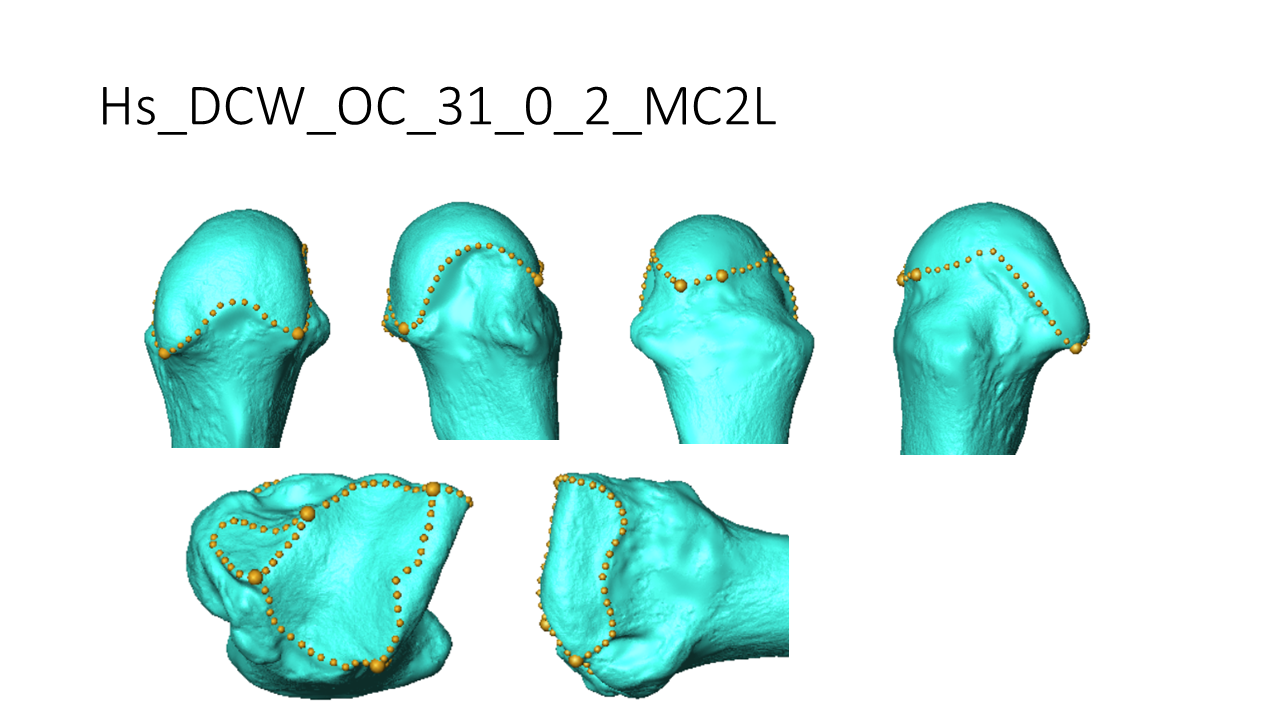


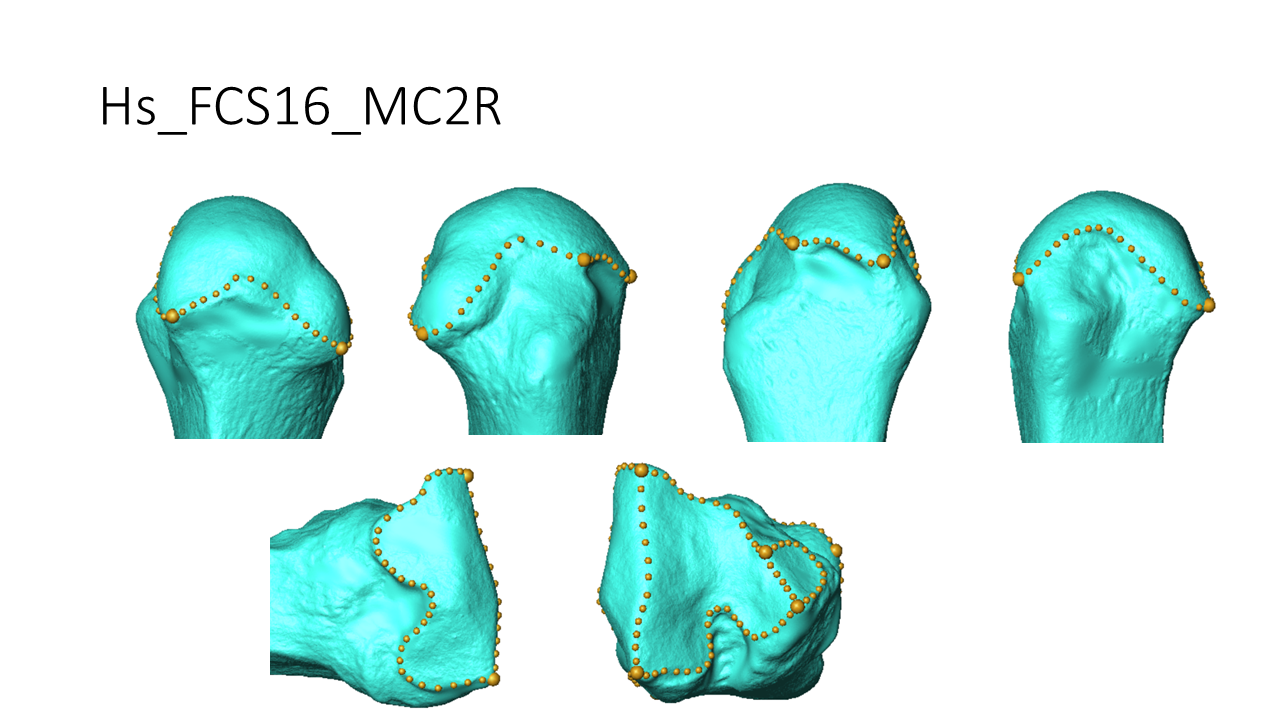


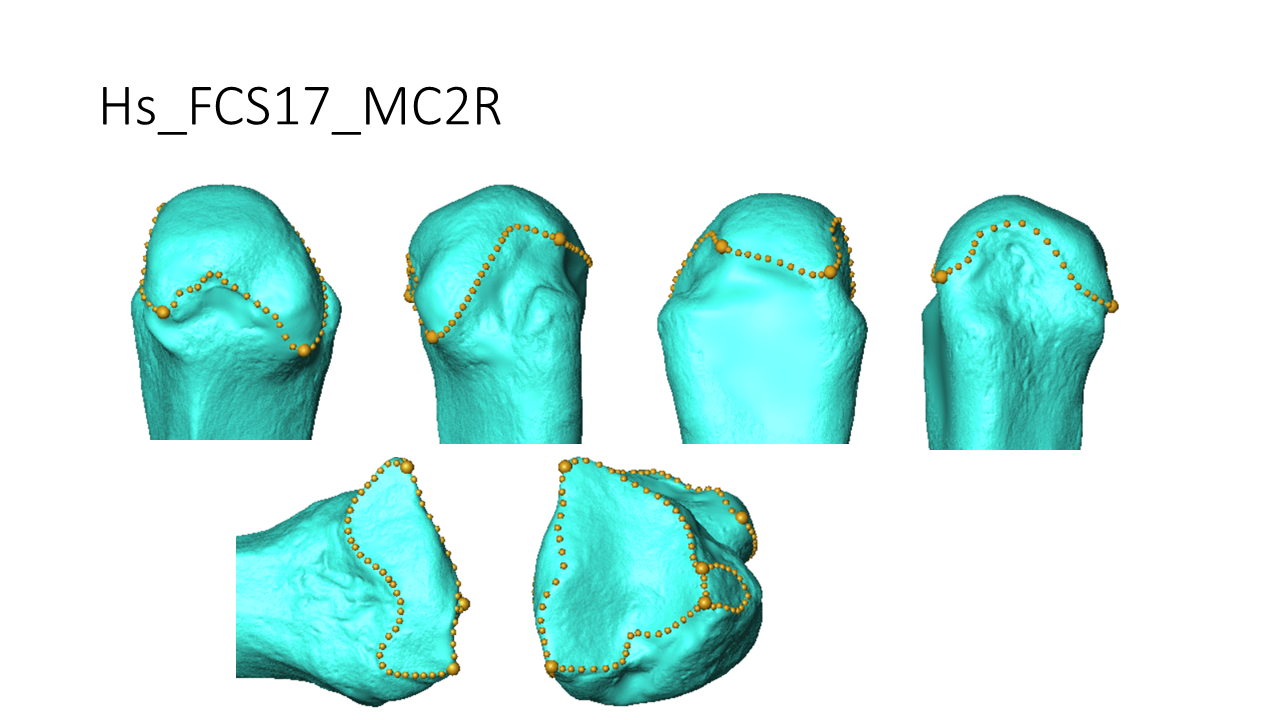


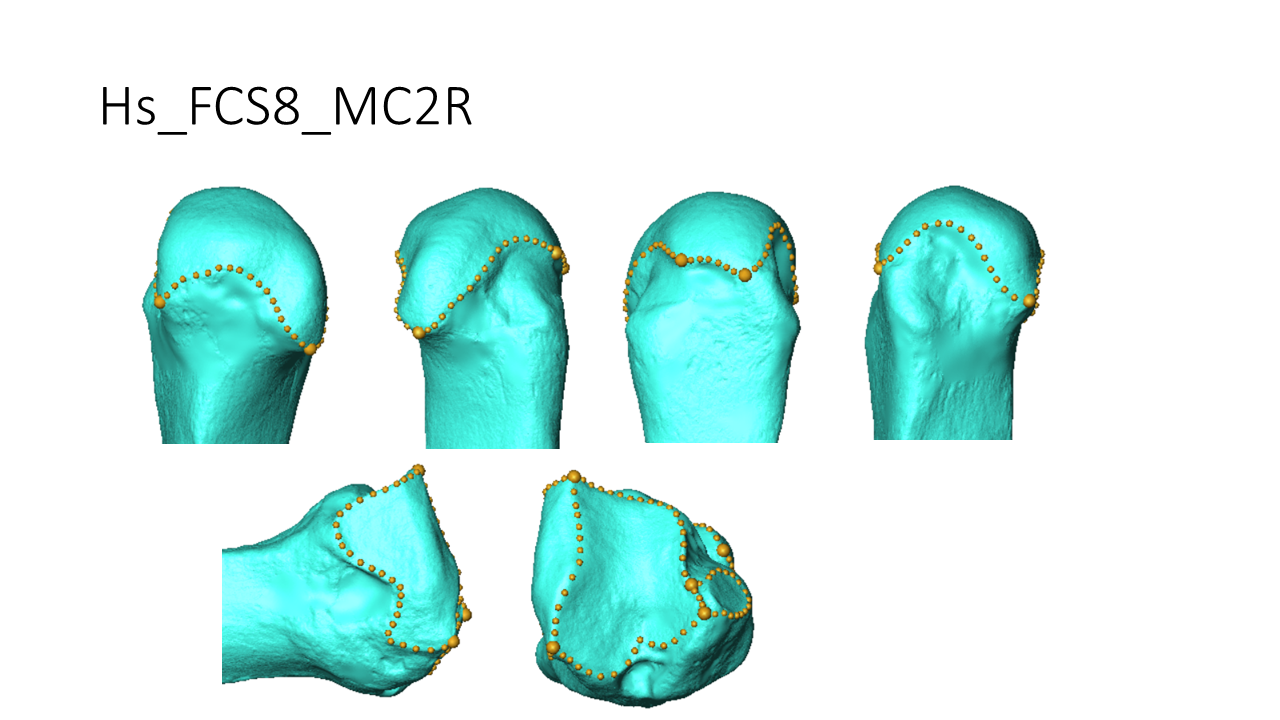


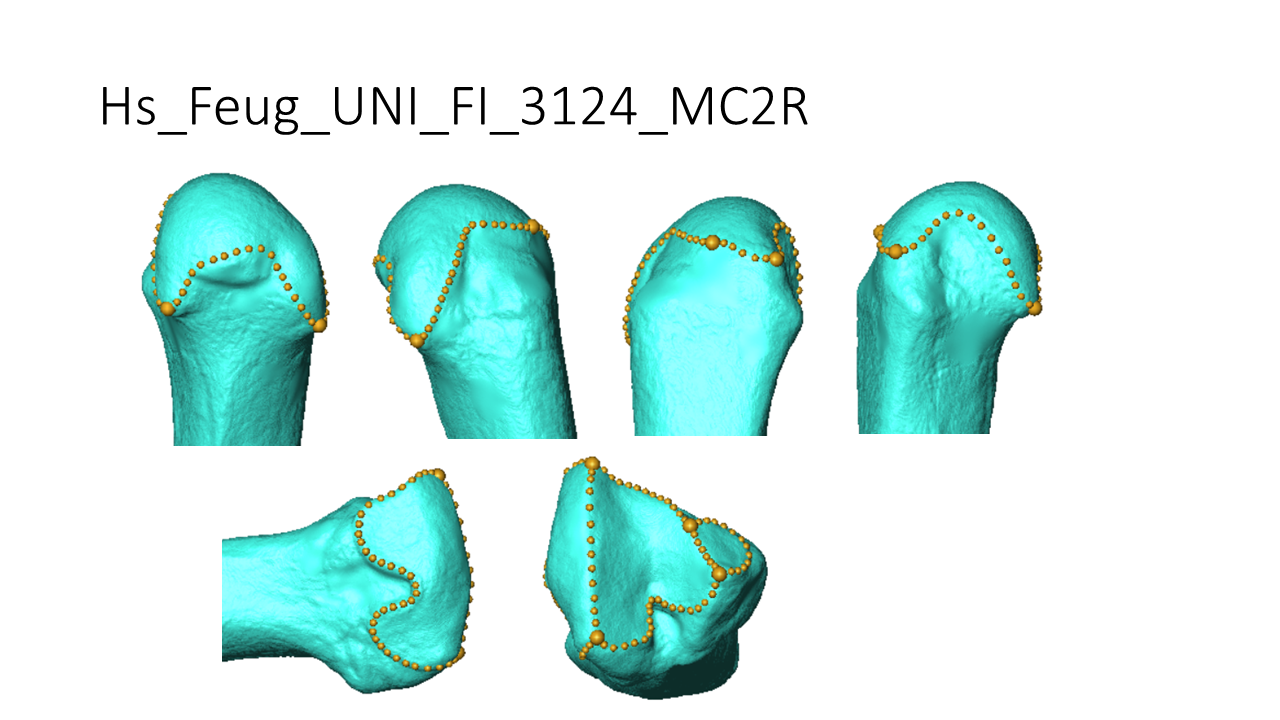

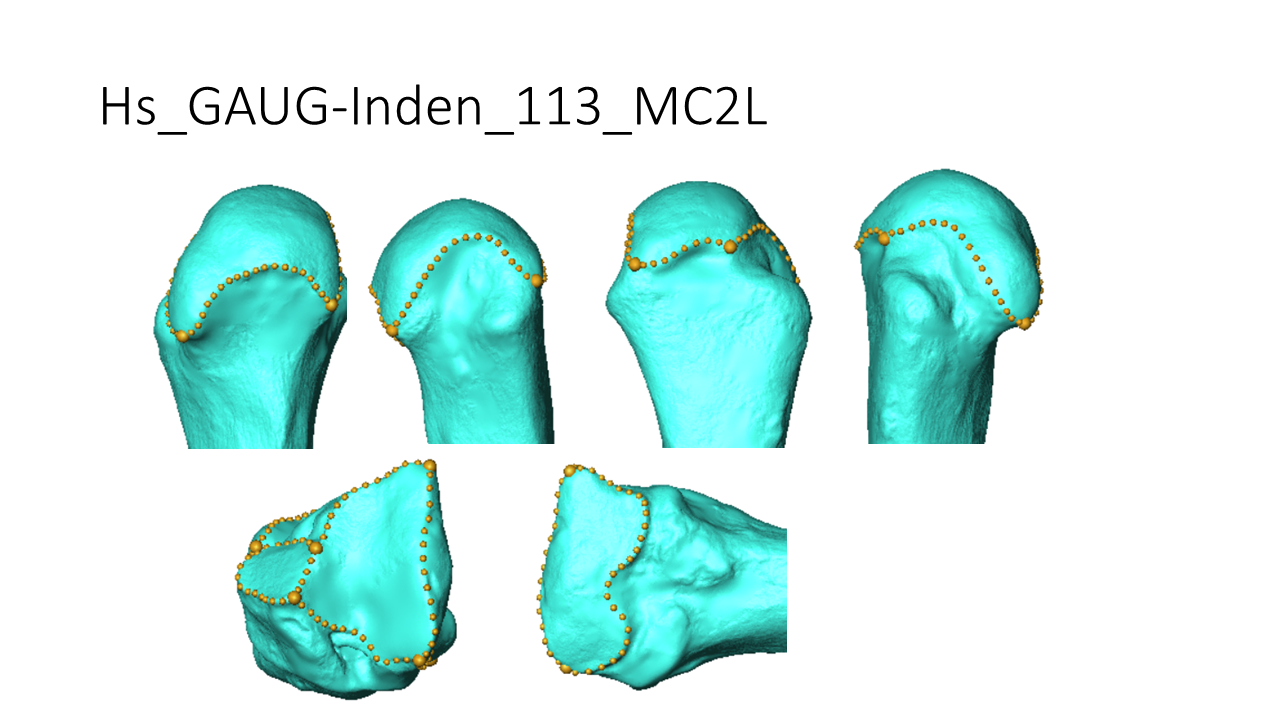


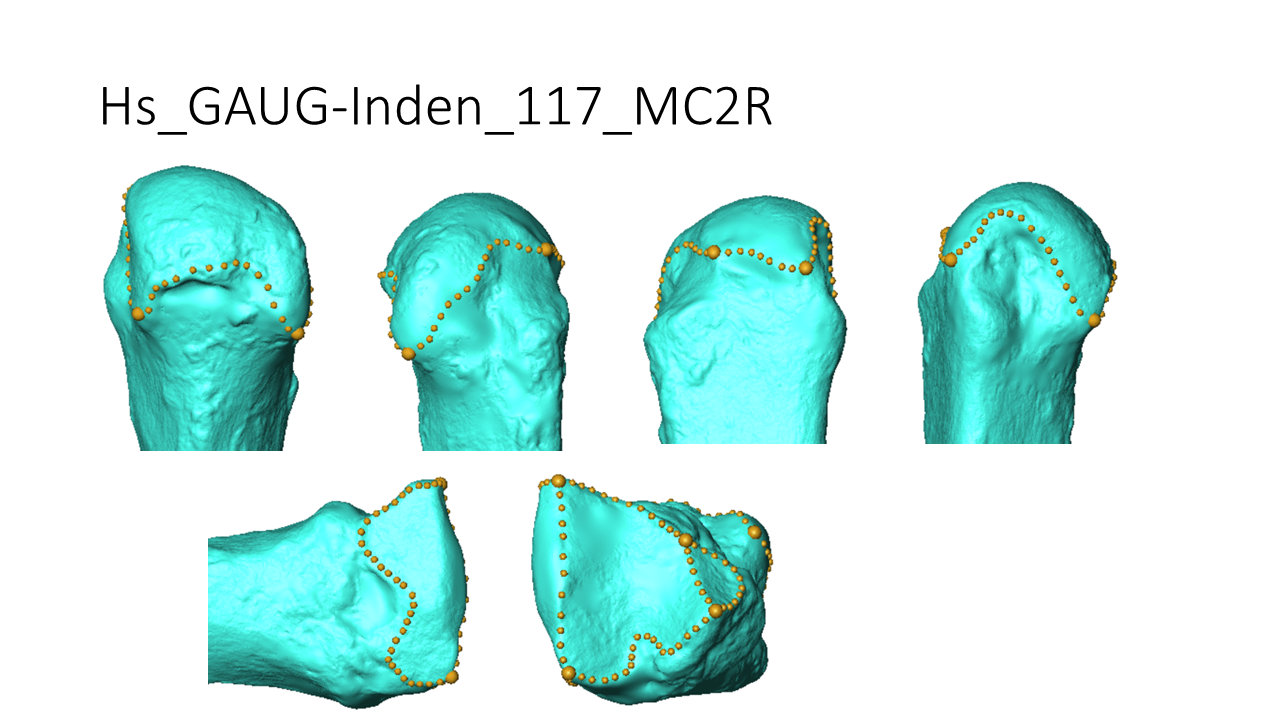

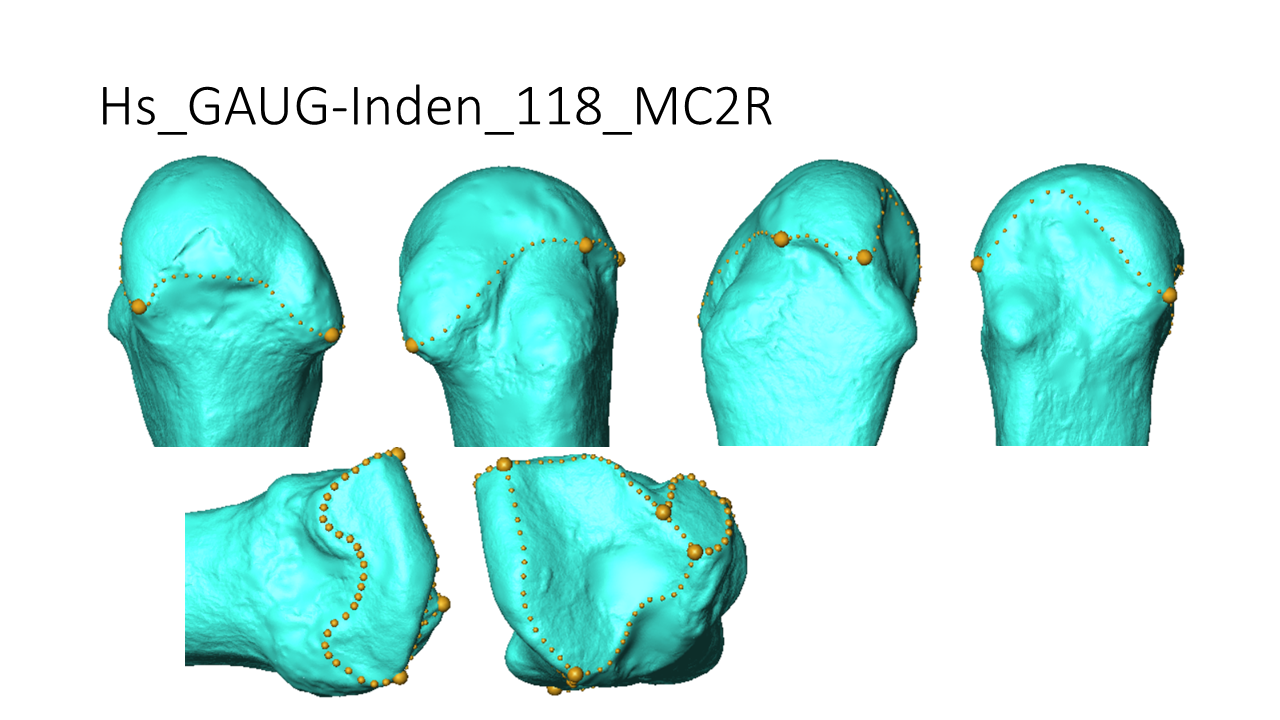

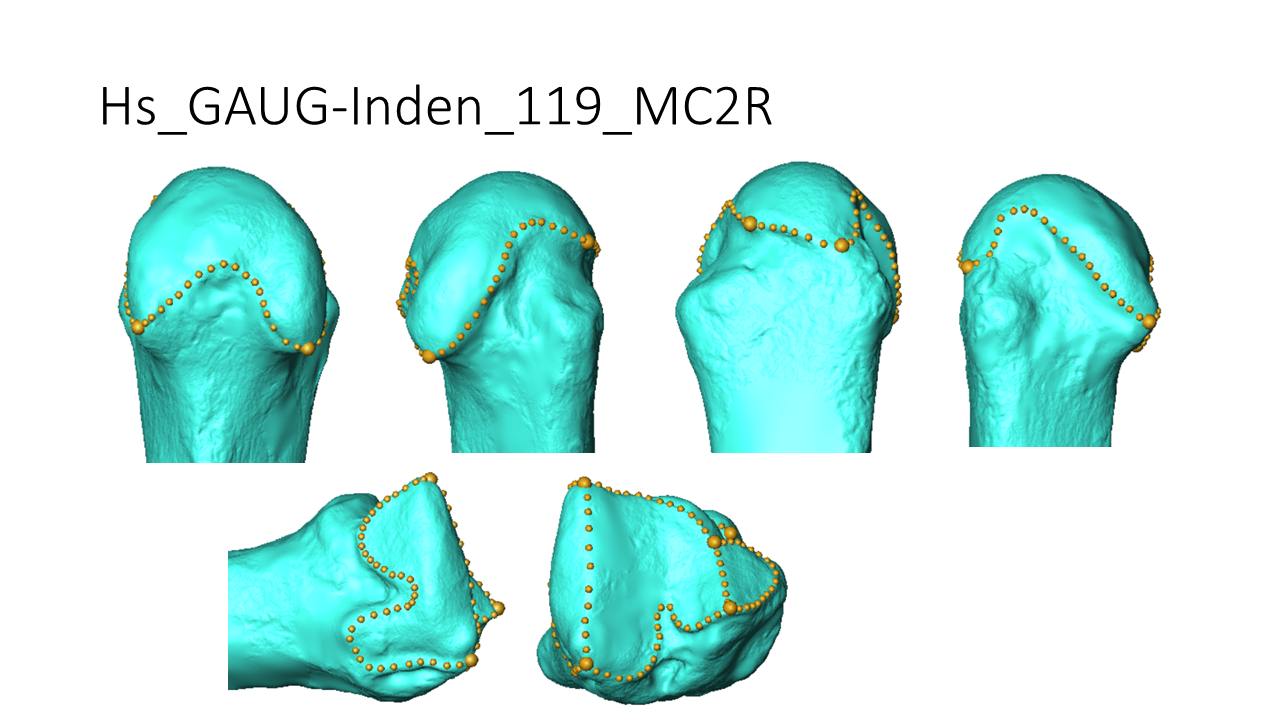


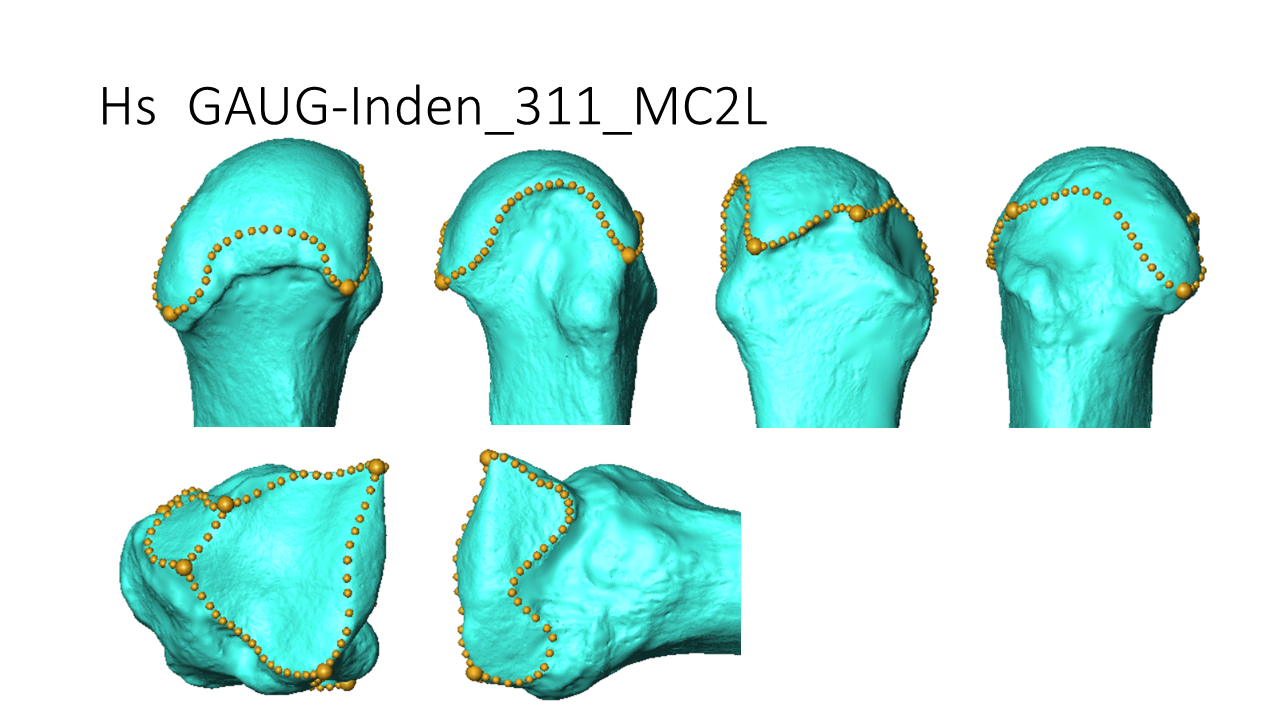

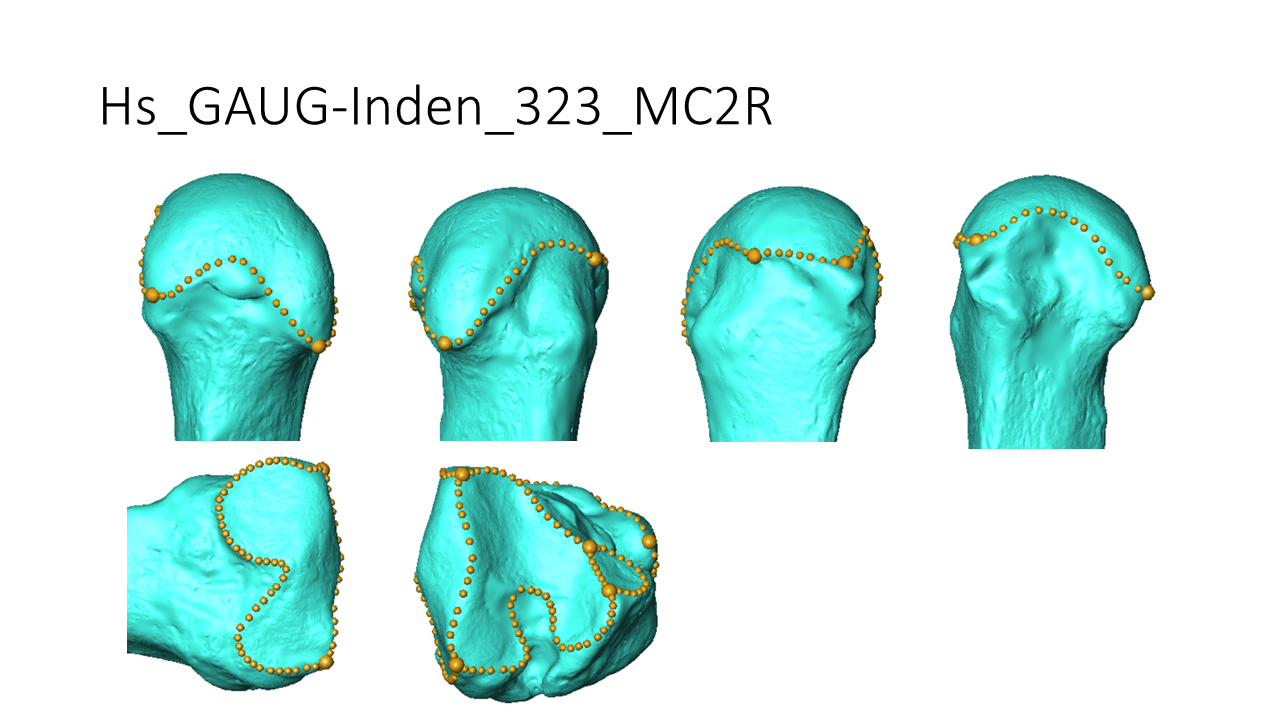

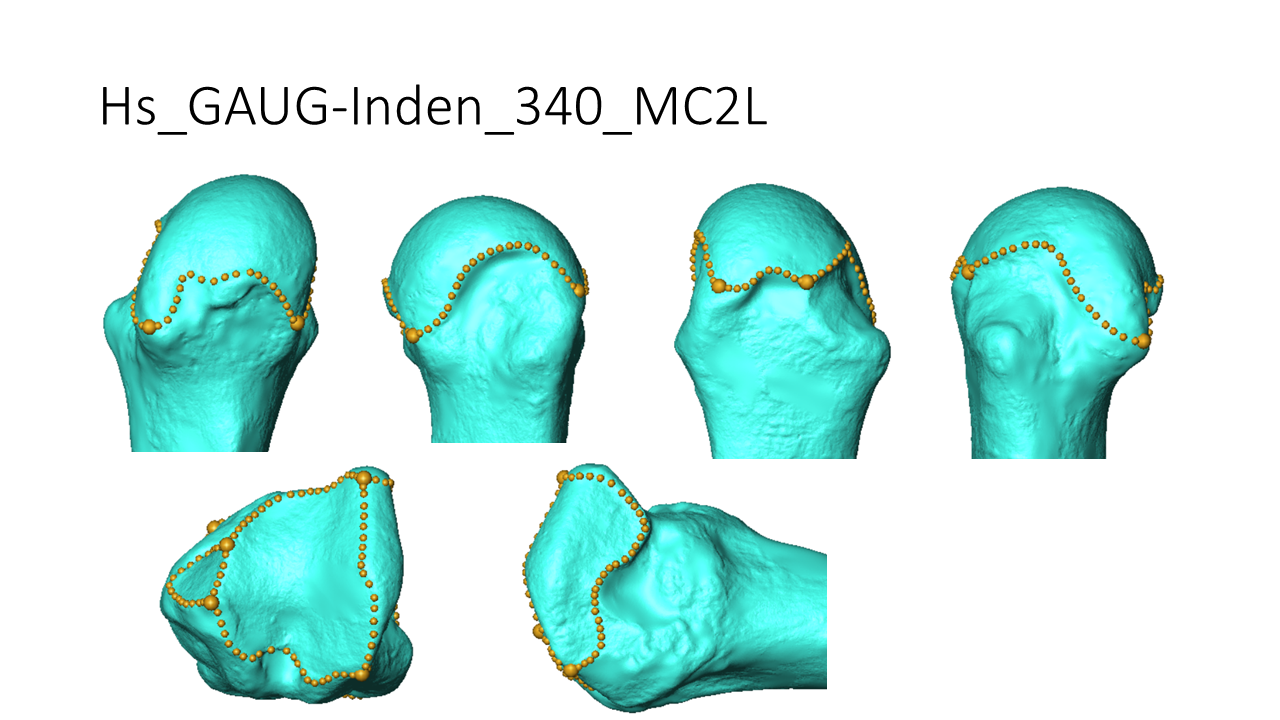


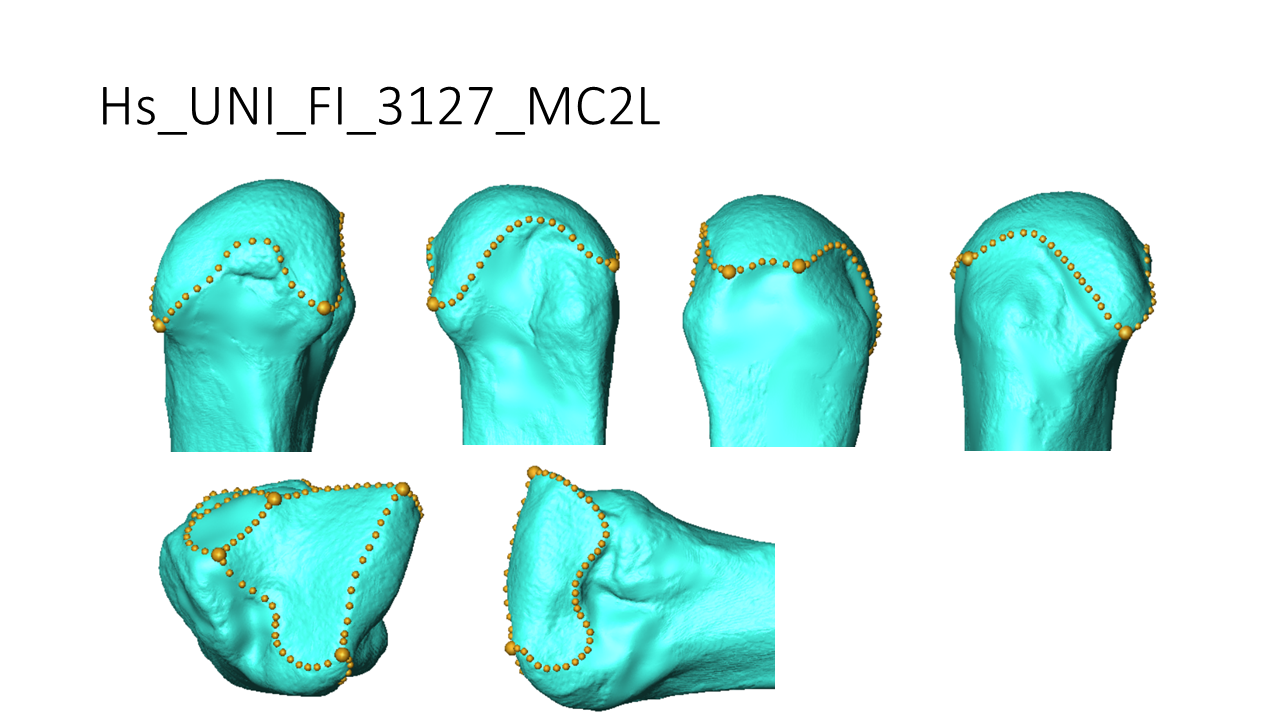

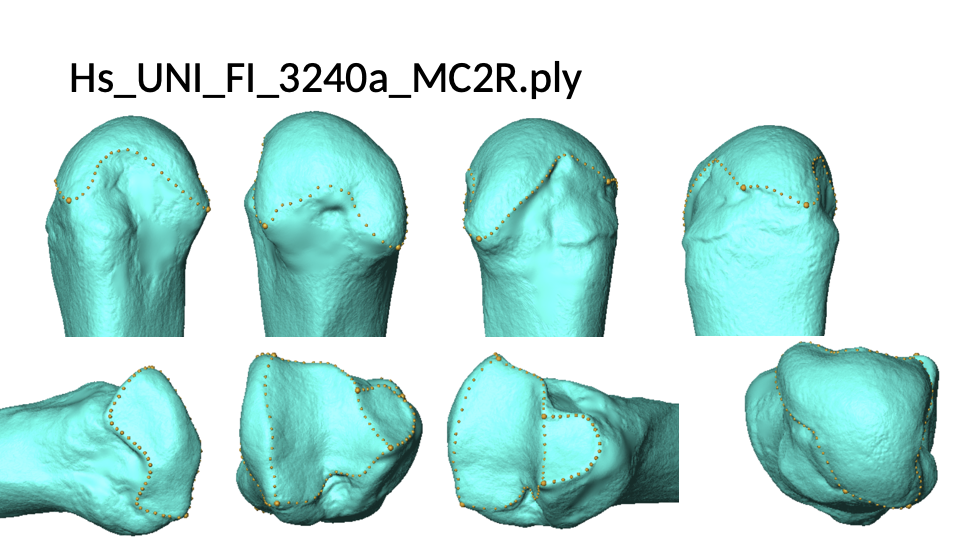

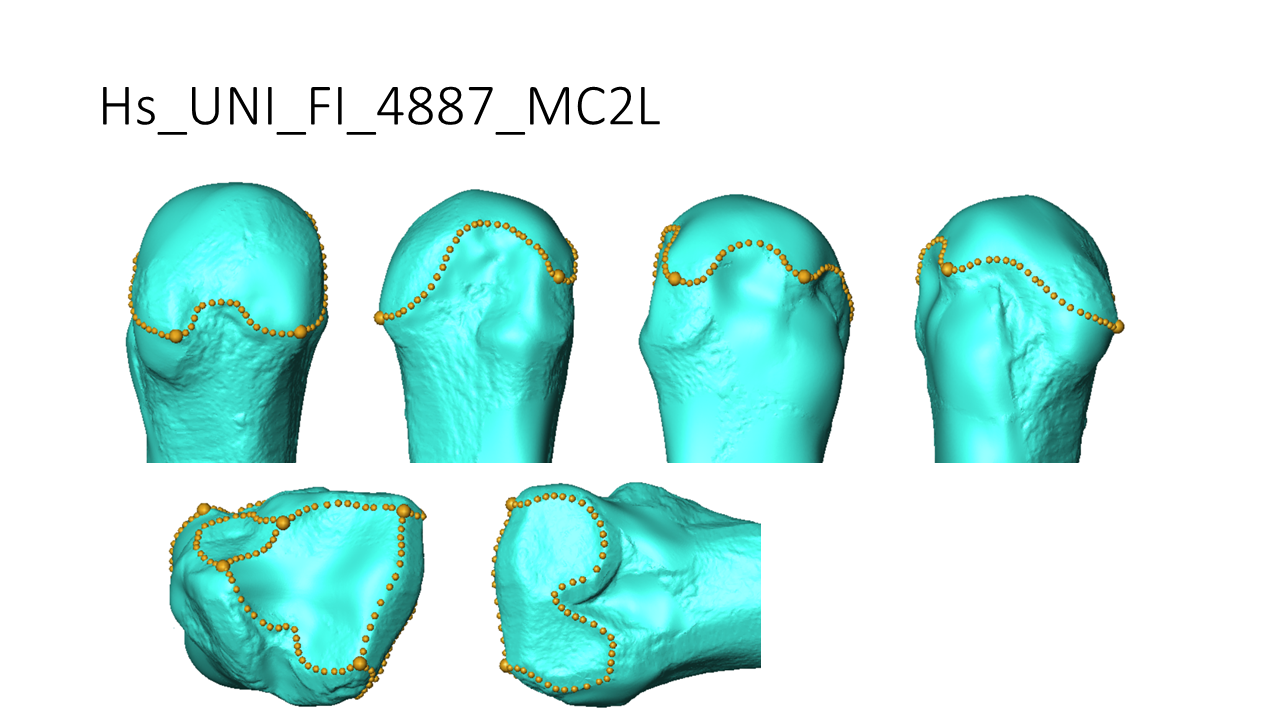


11.3. Atlas of all landmarked third metacarpals (n=26)


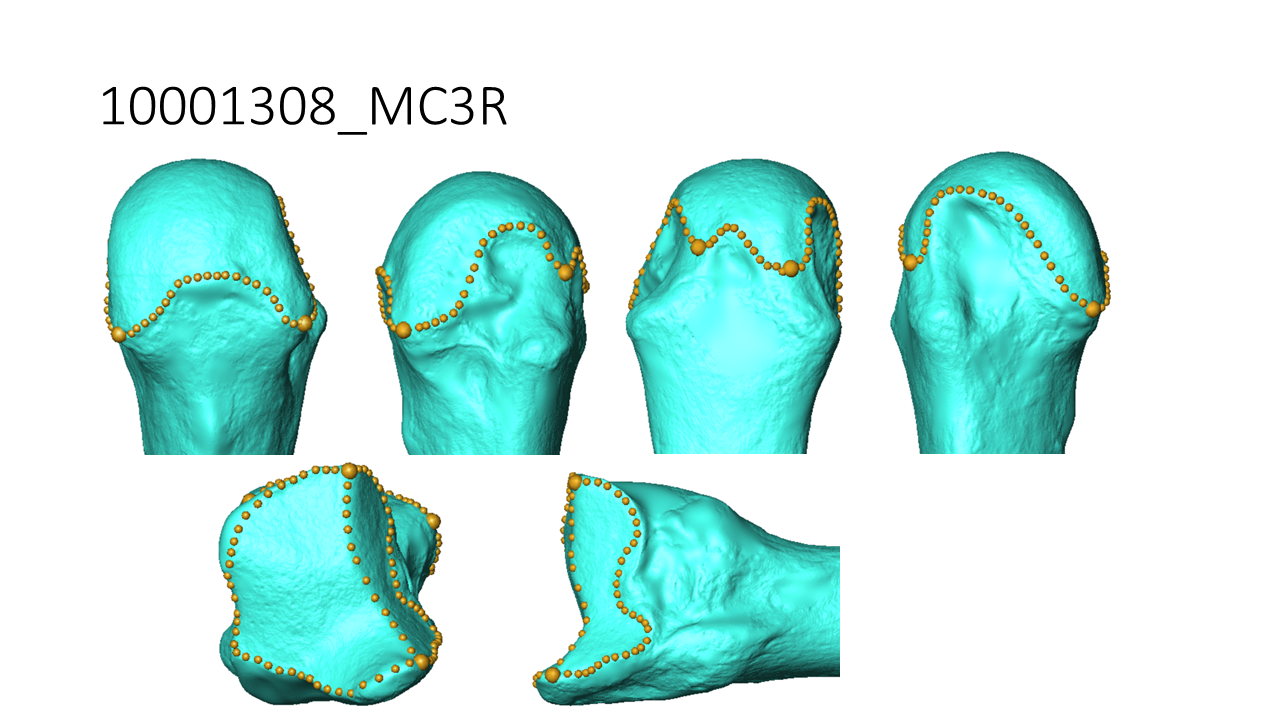

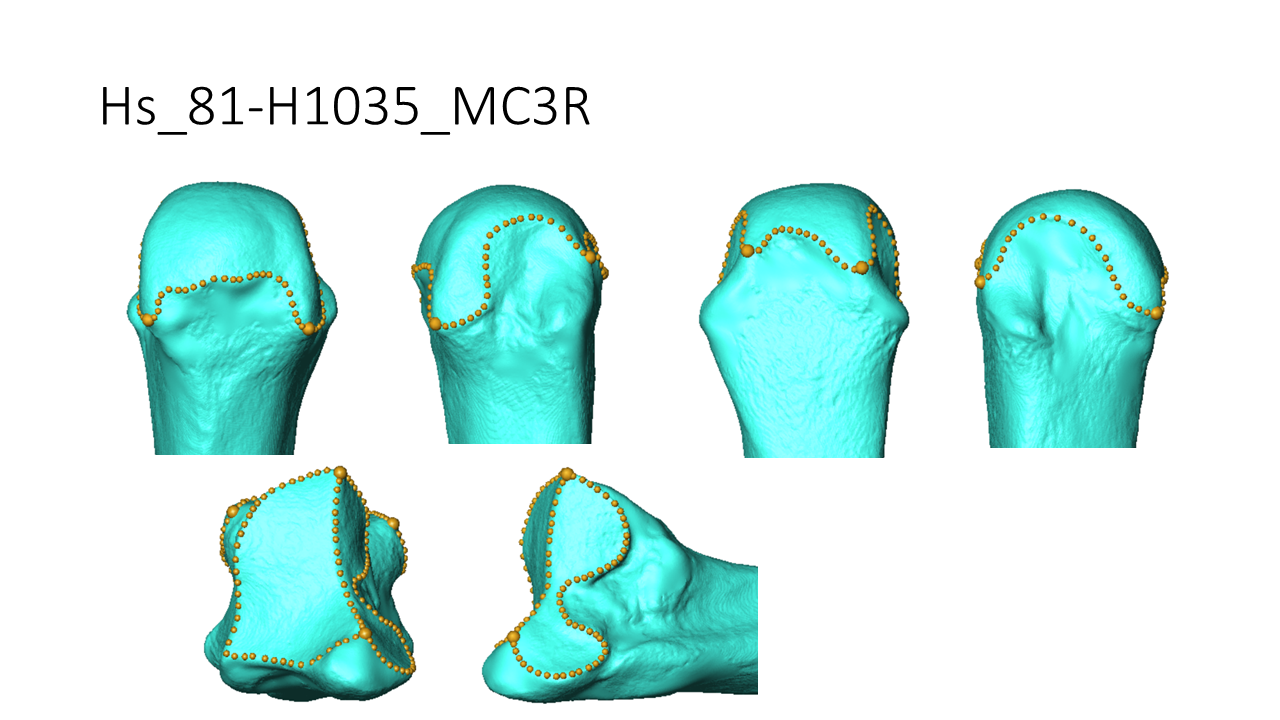

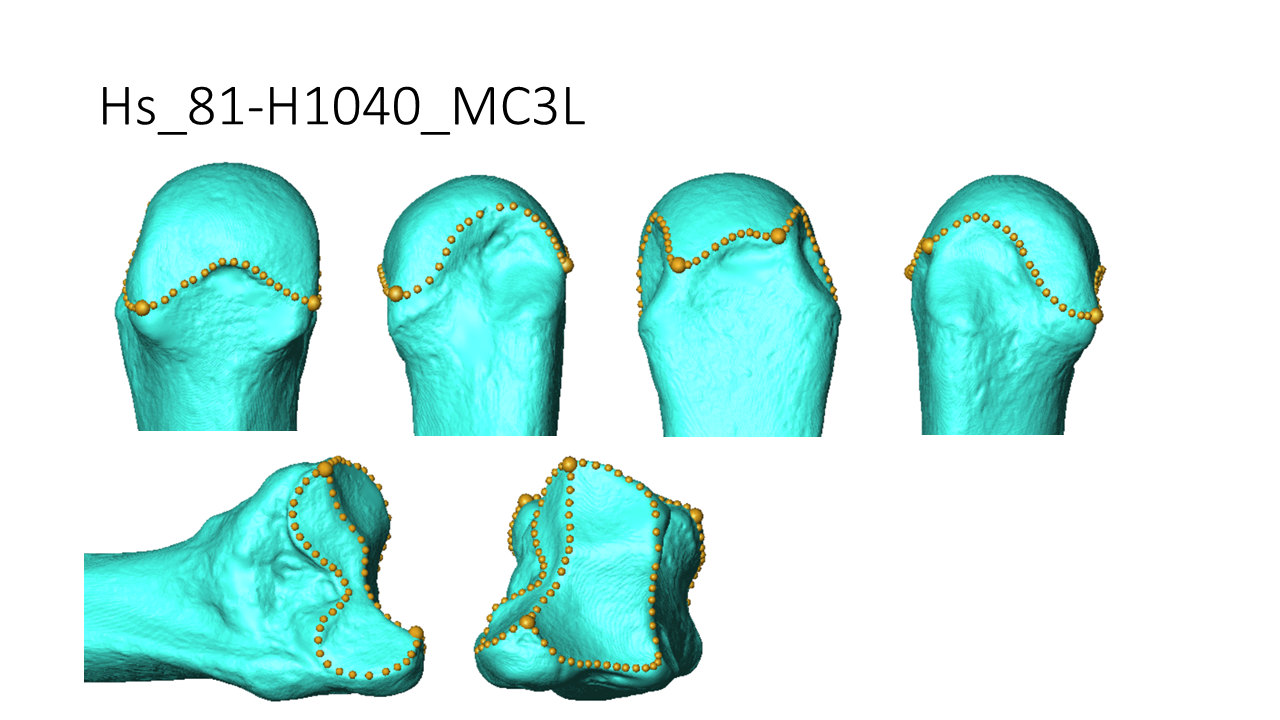

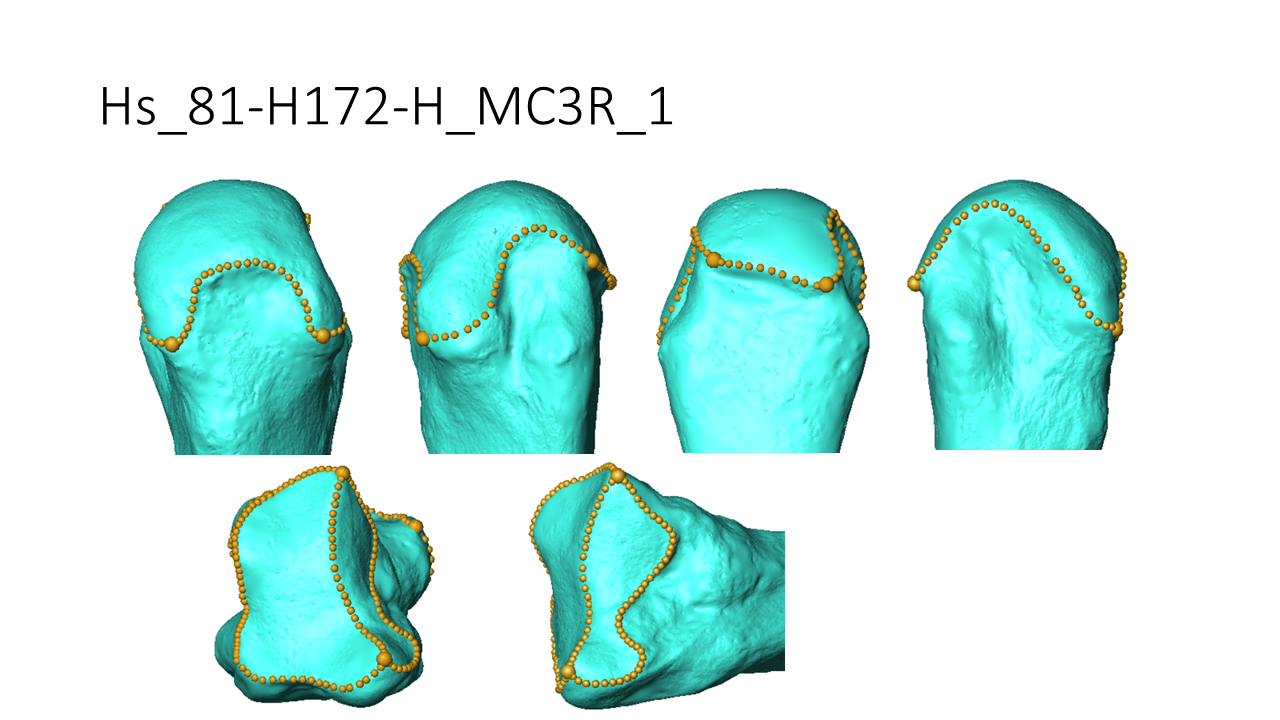


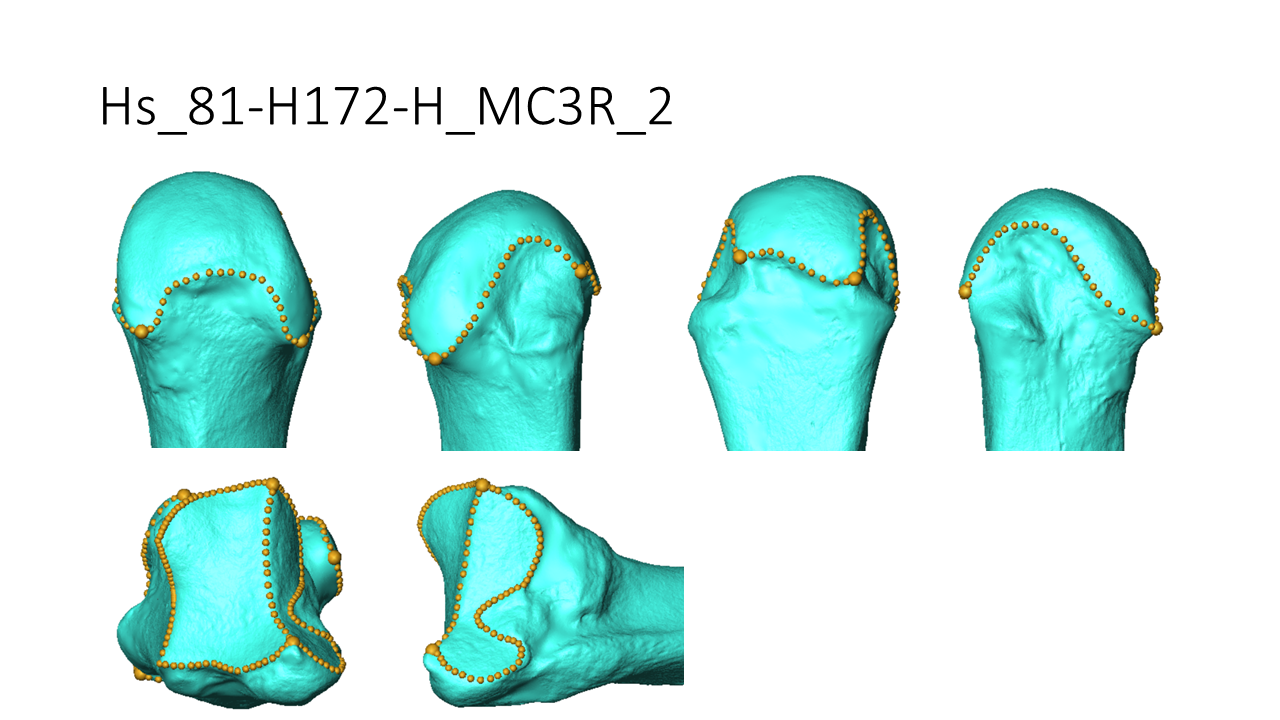

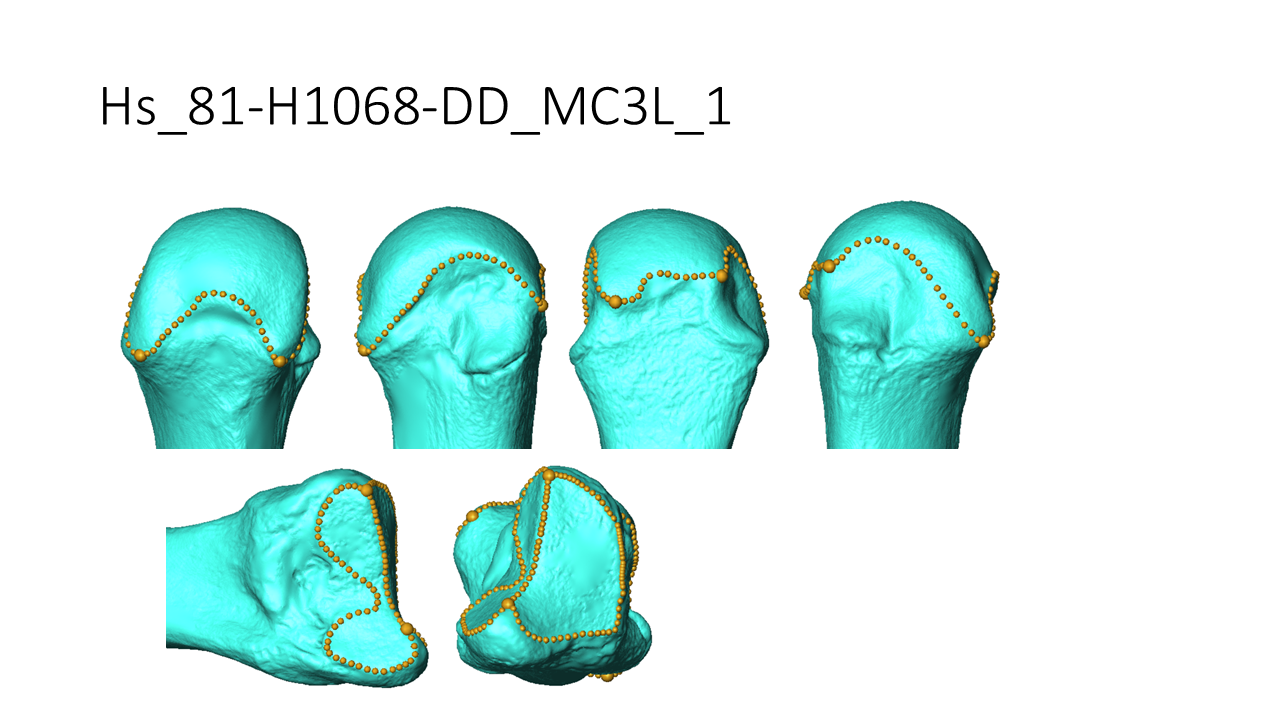

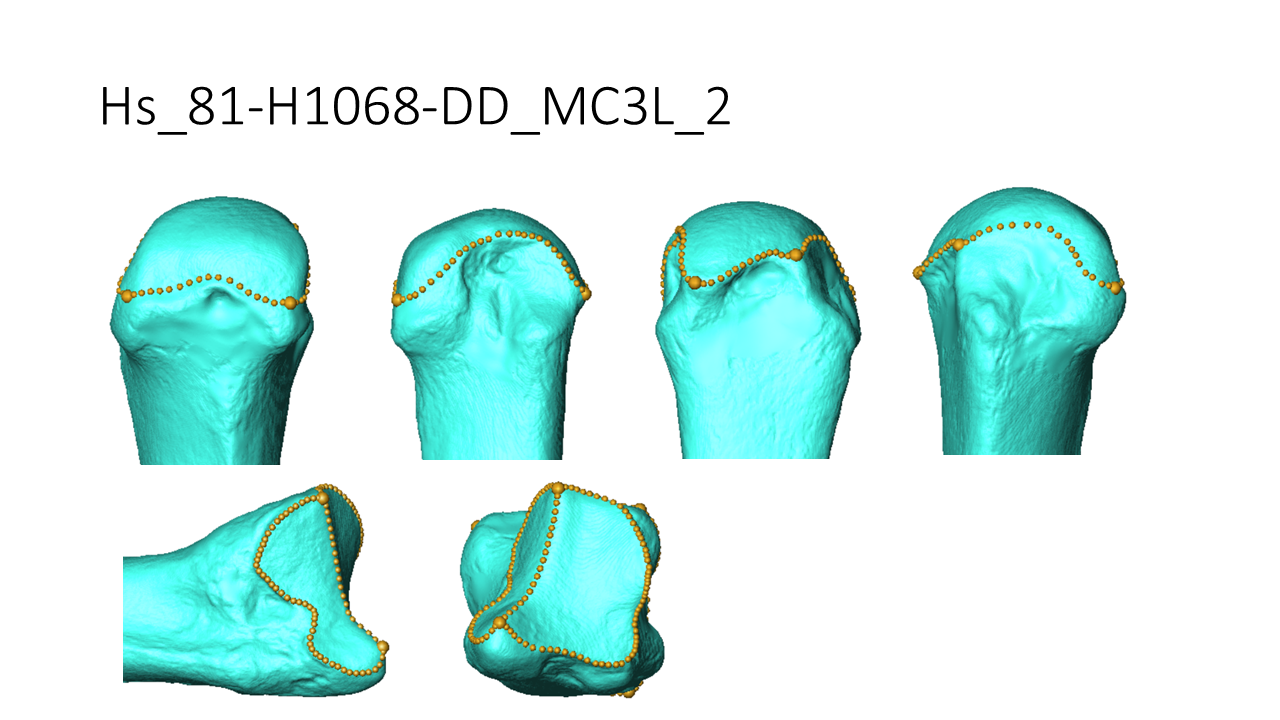

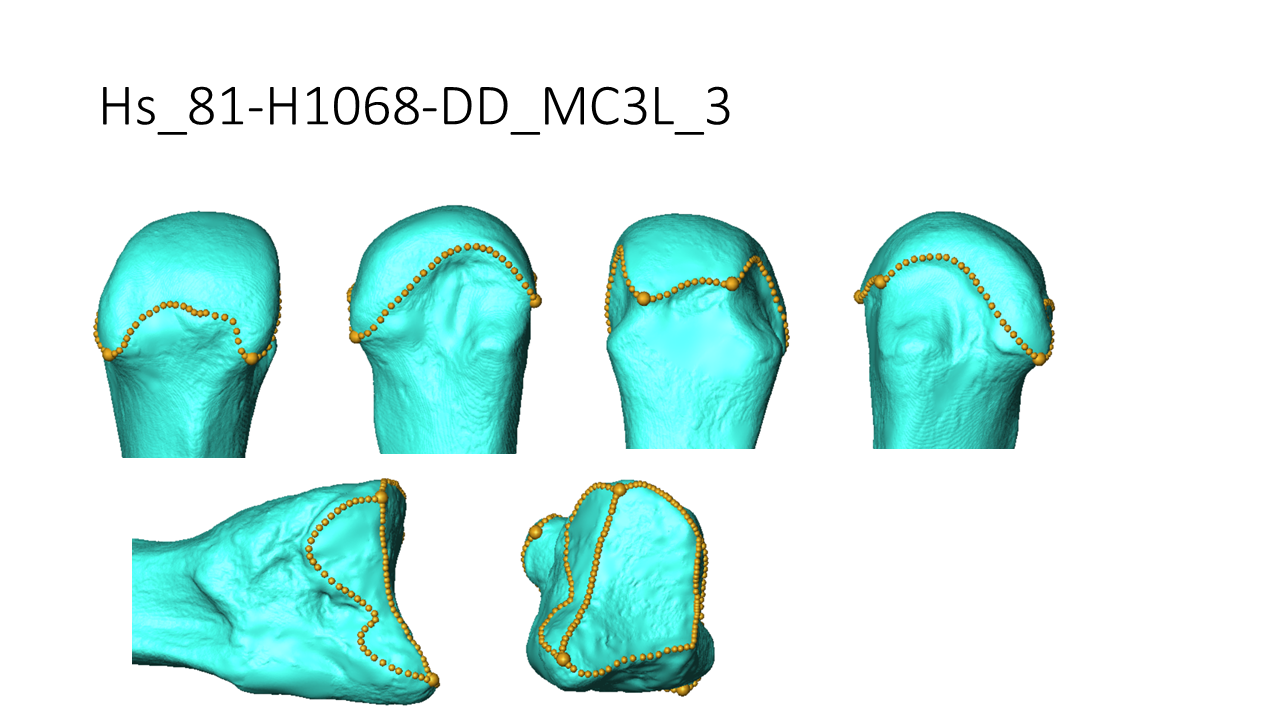


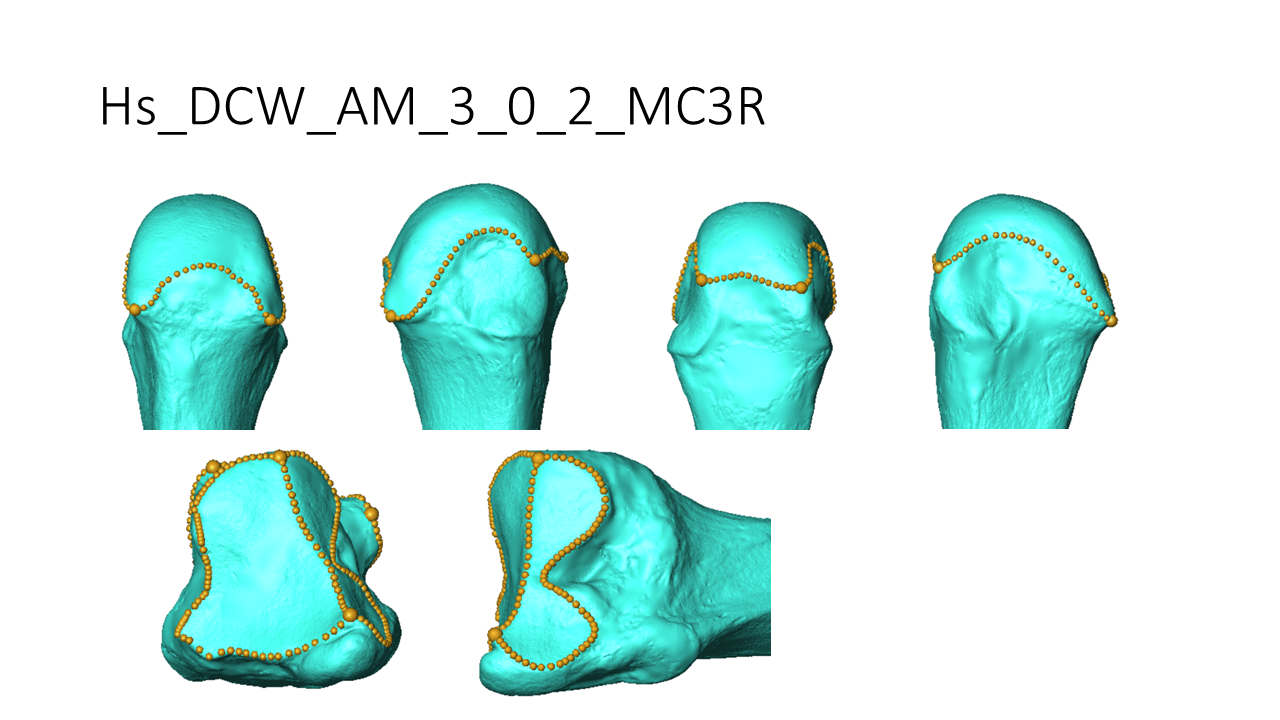

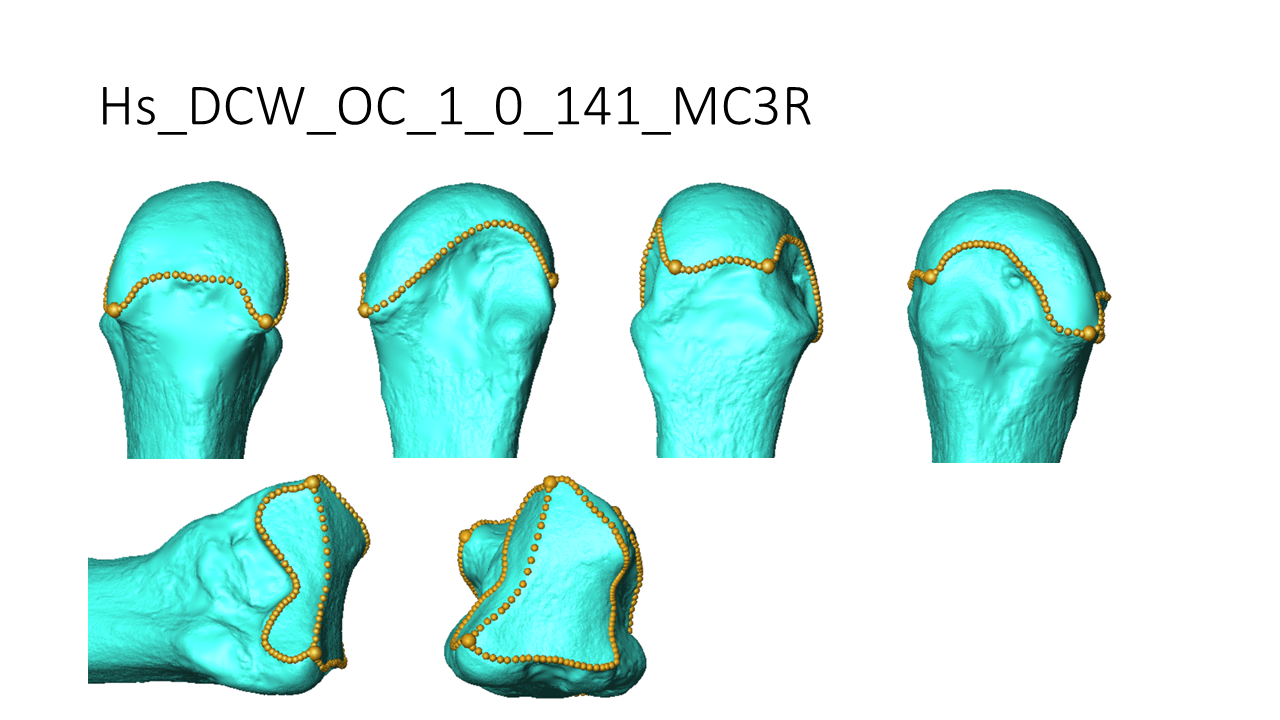


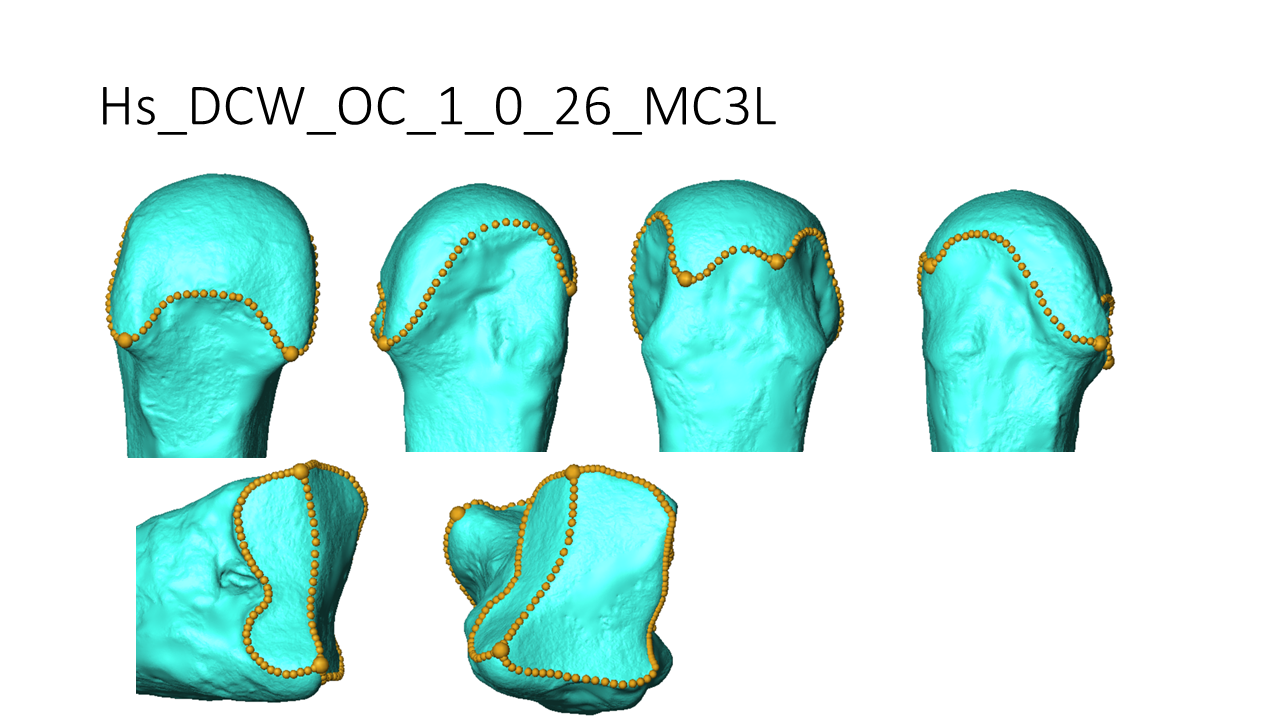


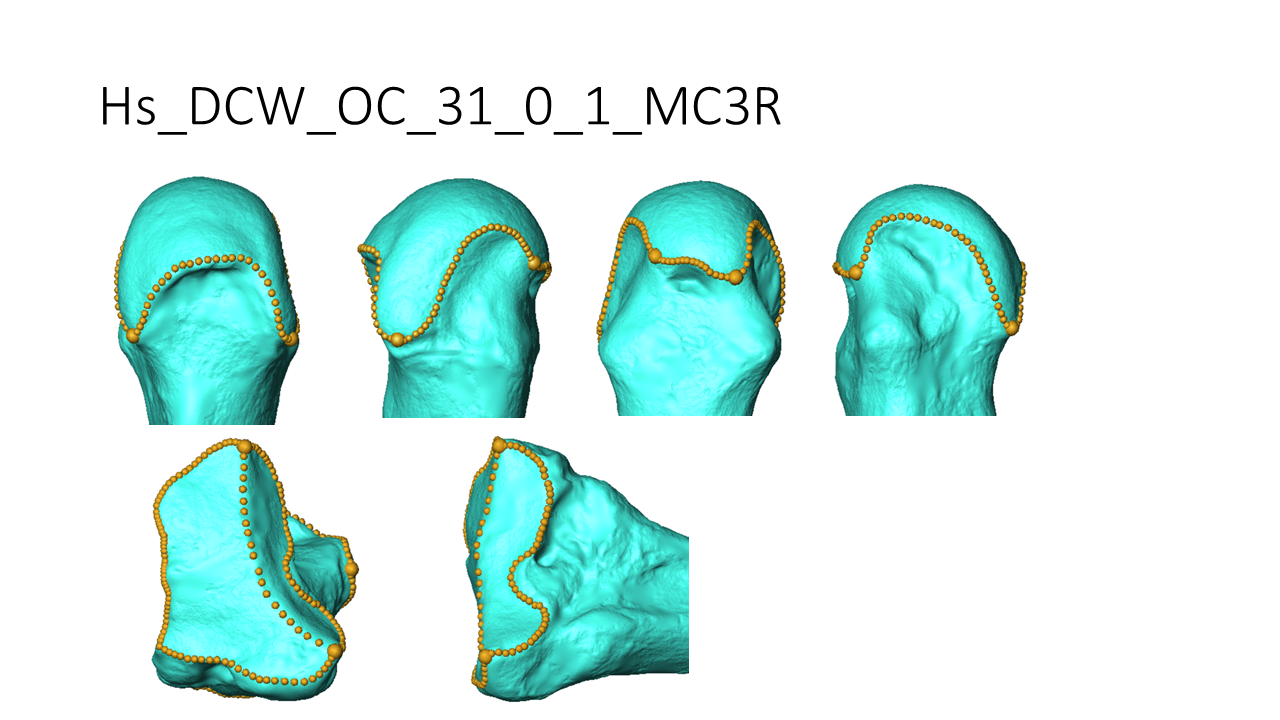


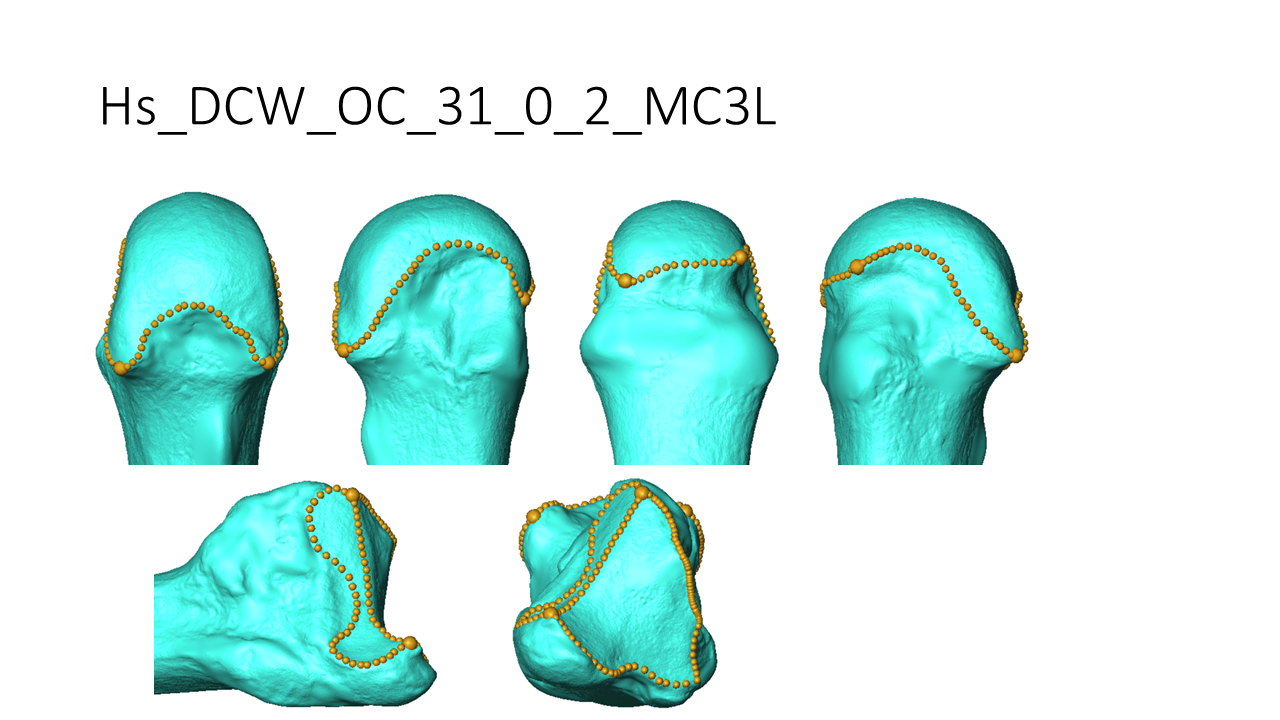


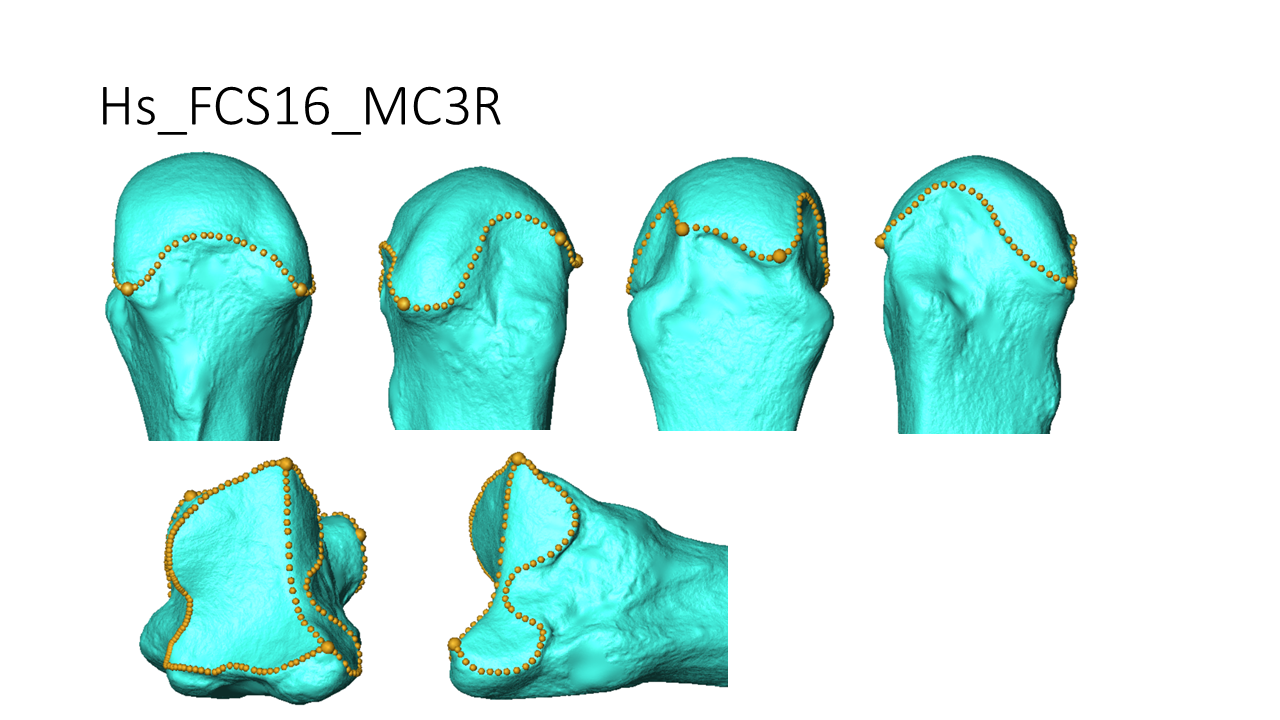

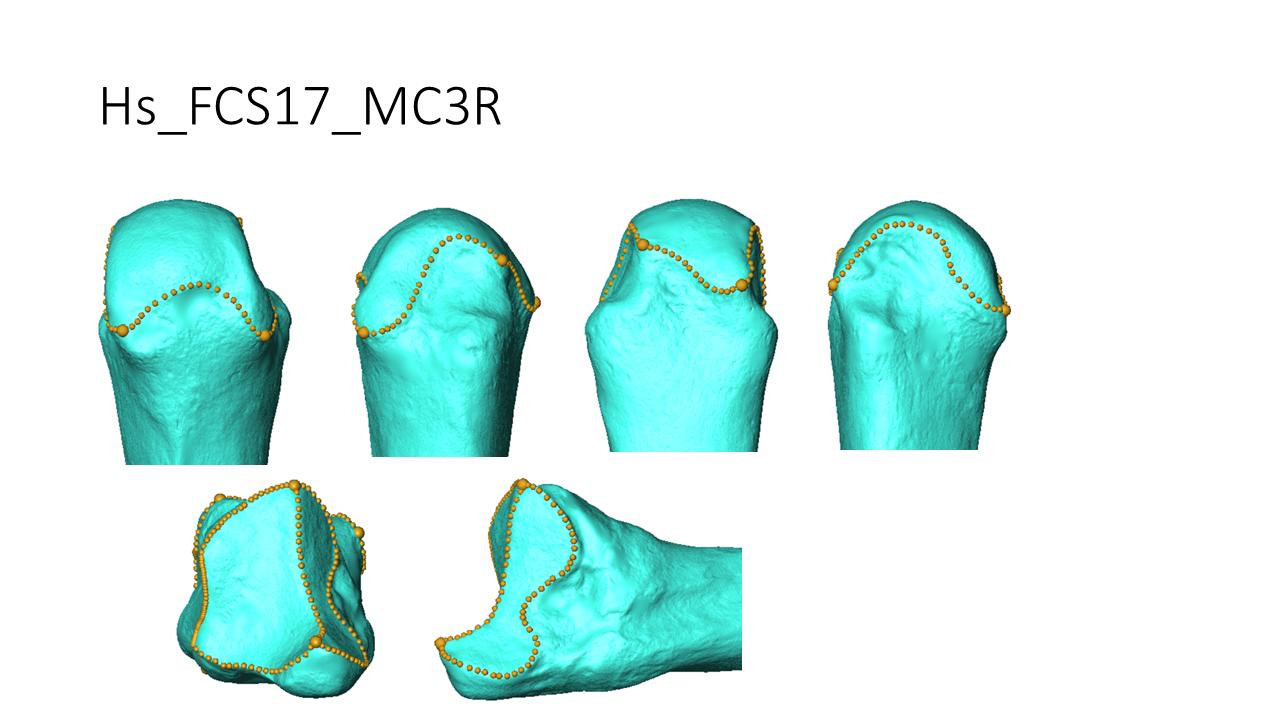

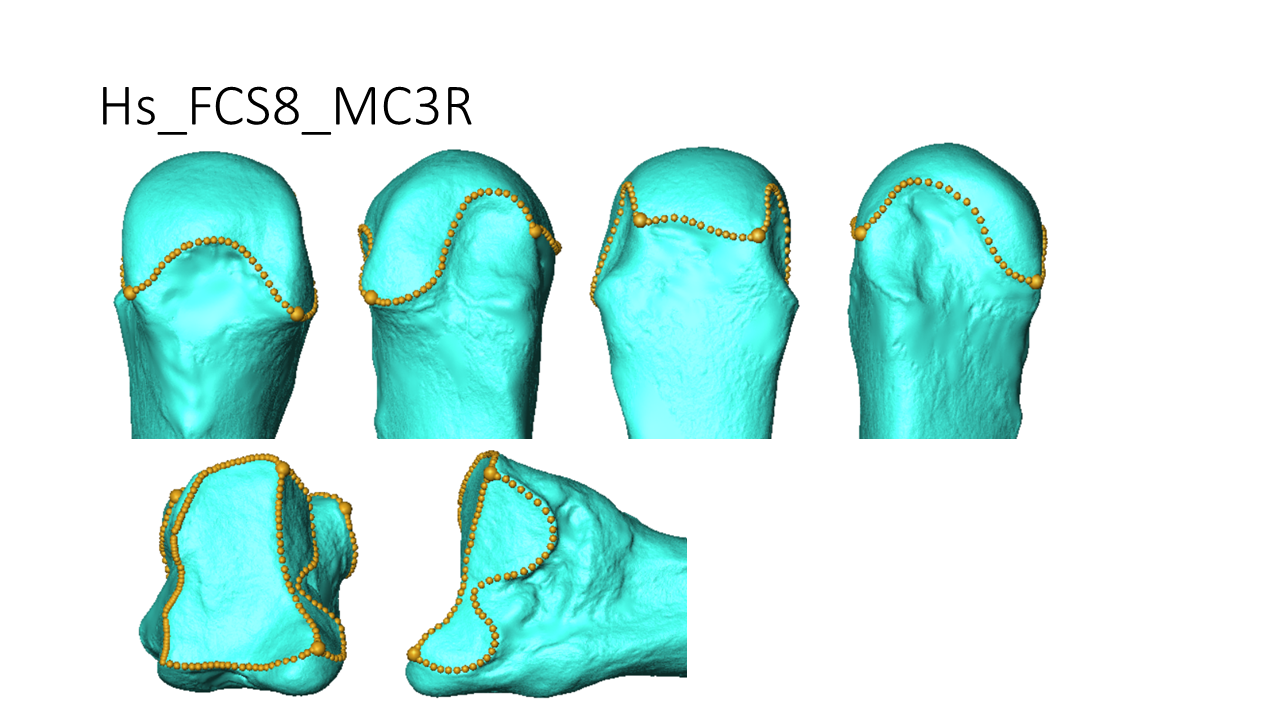


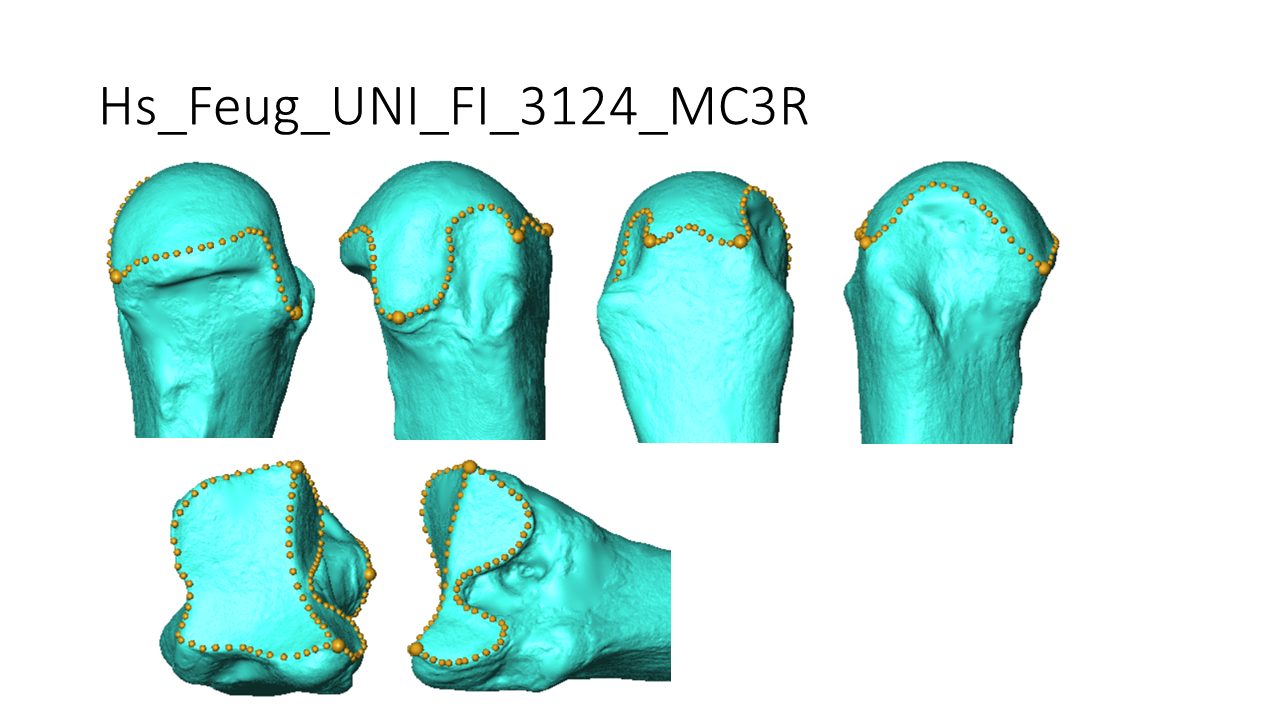

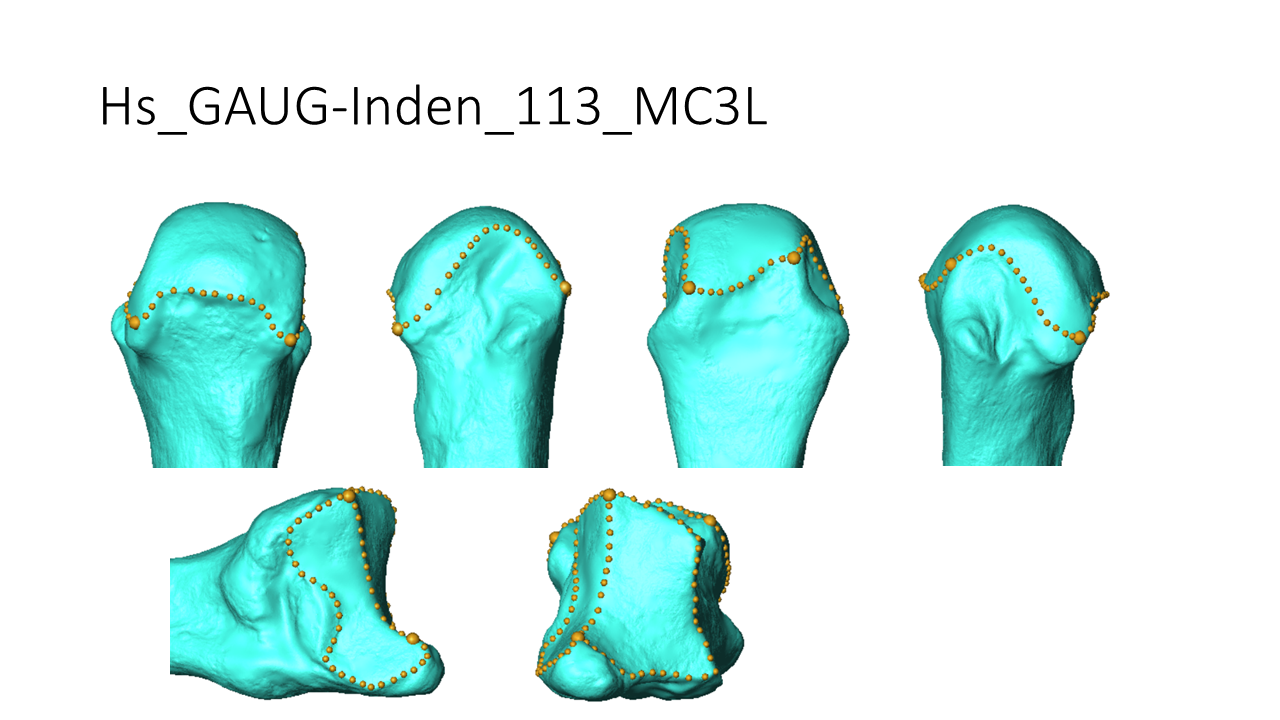

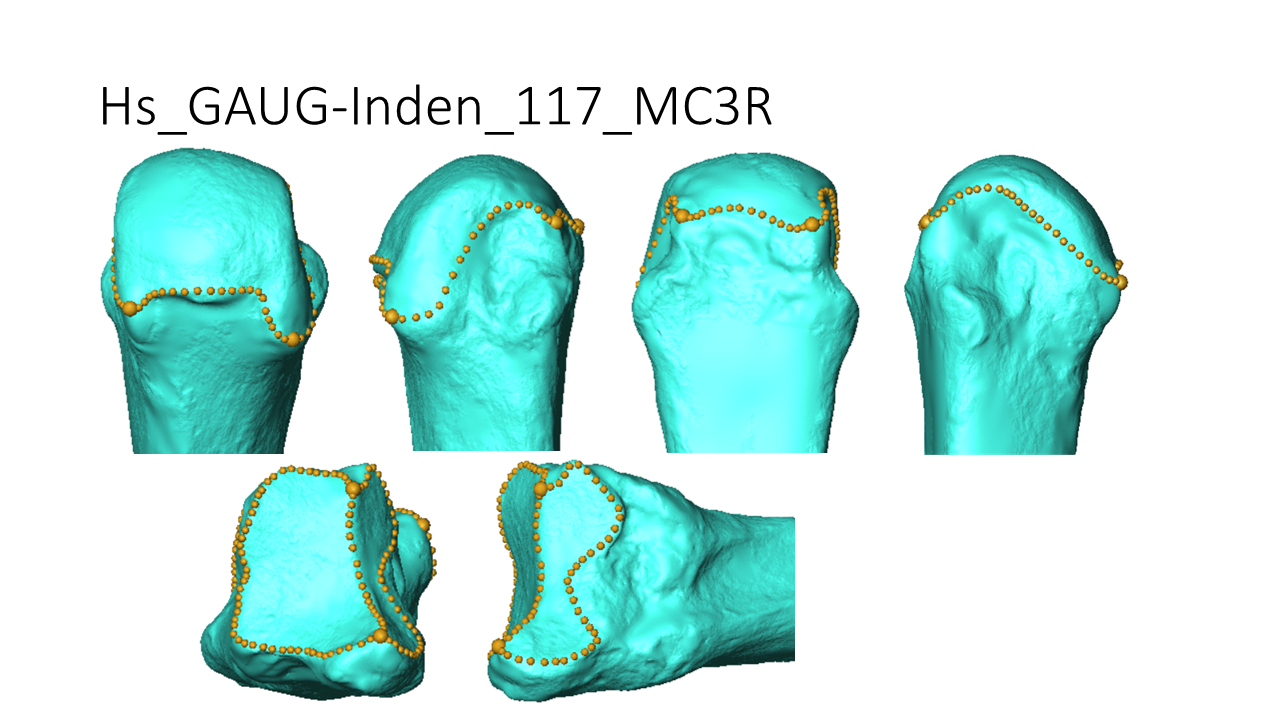

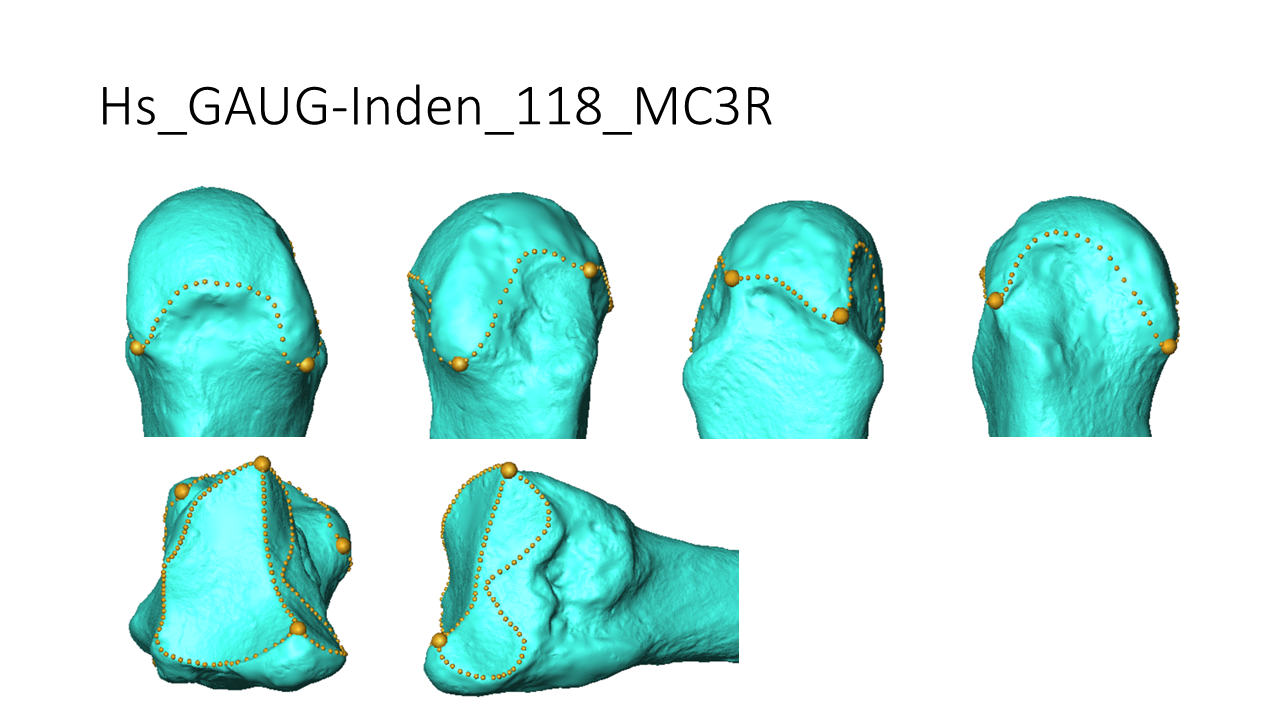


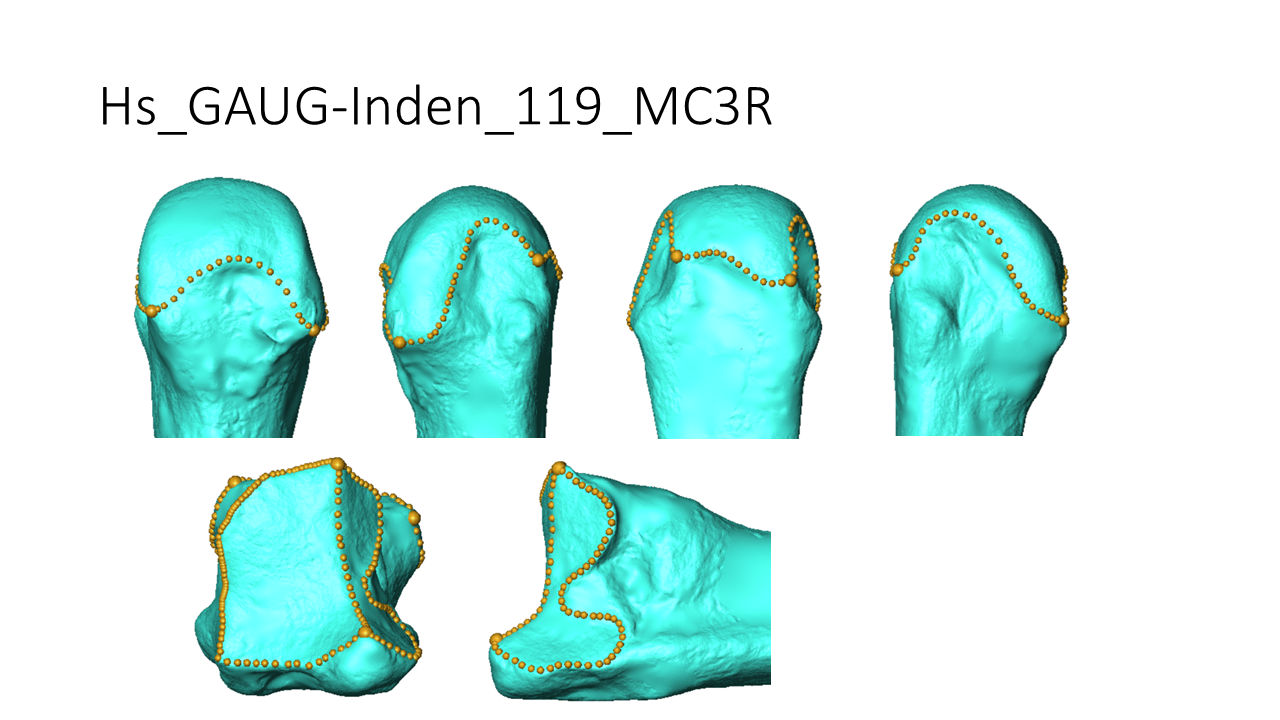

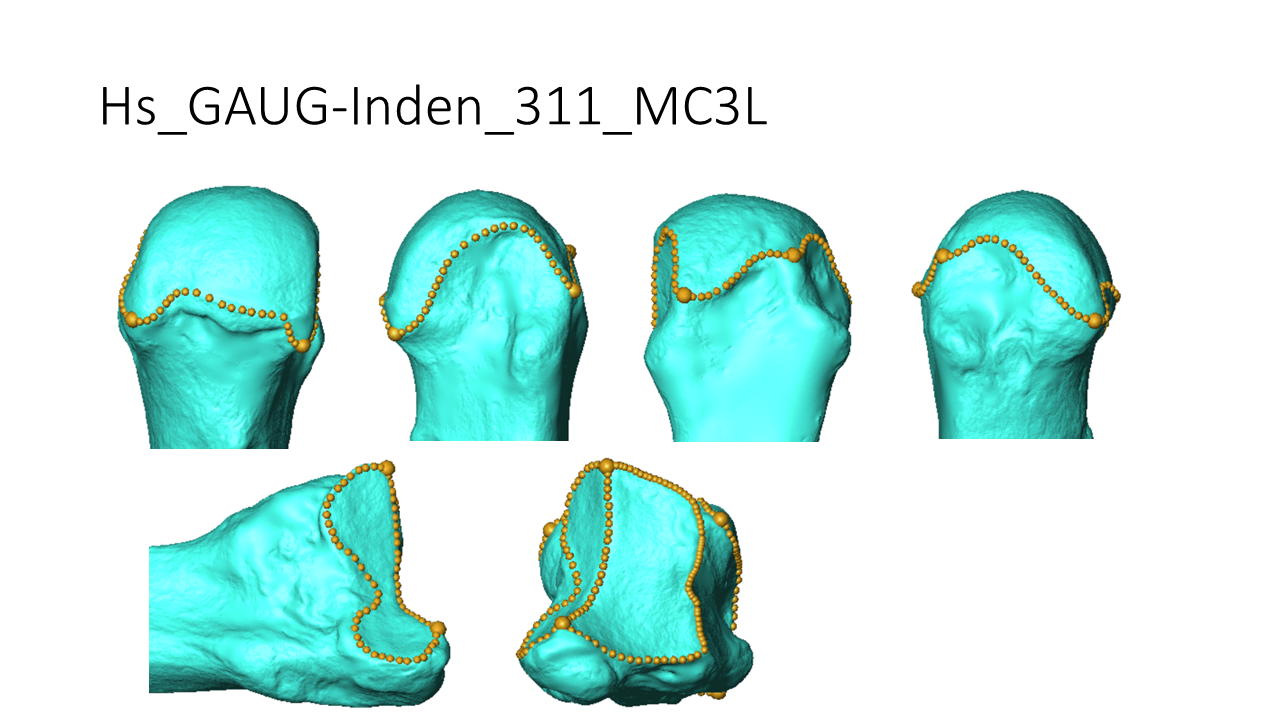

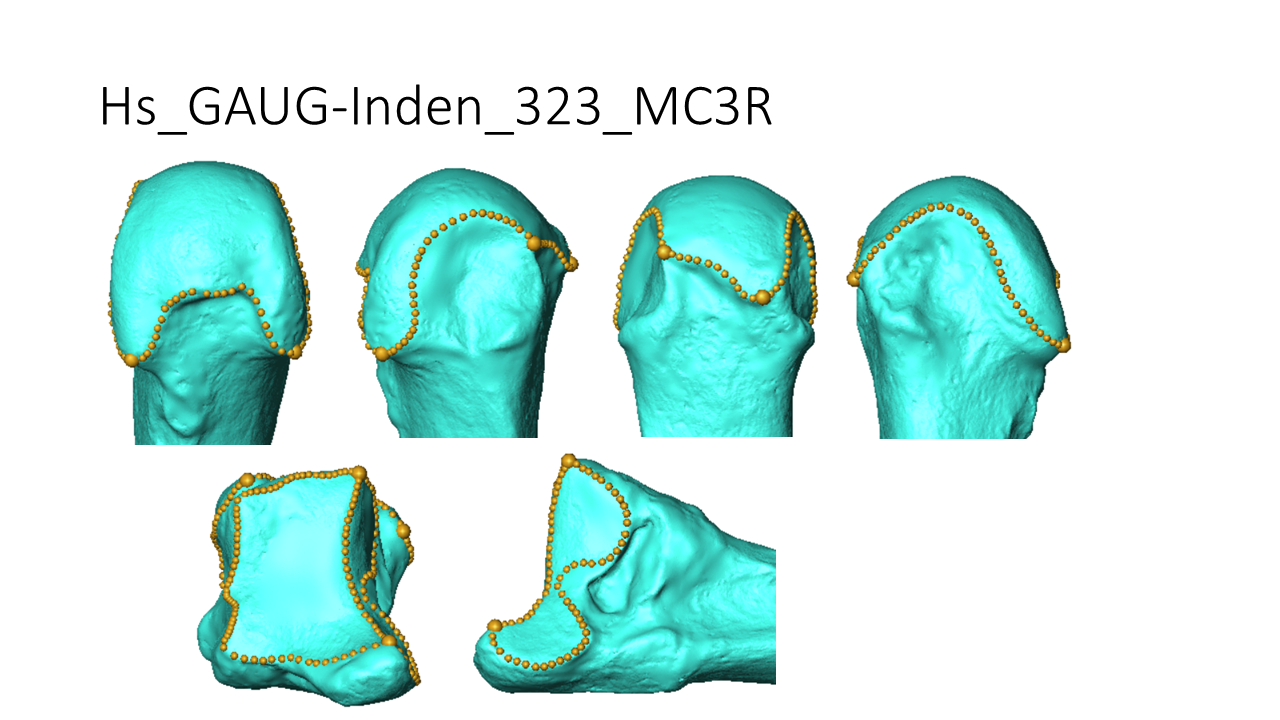

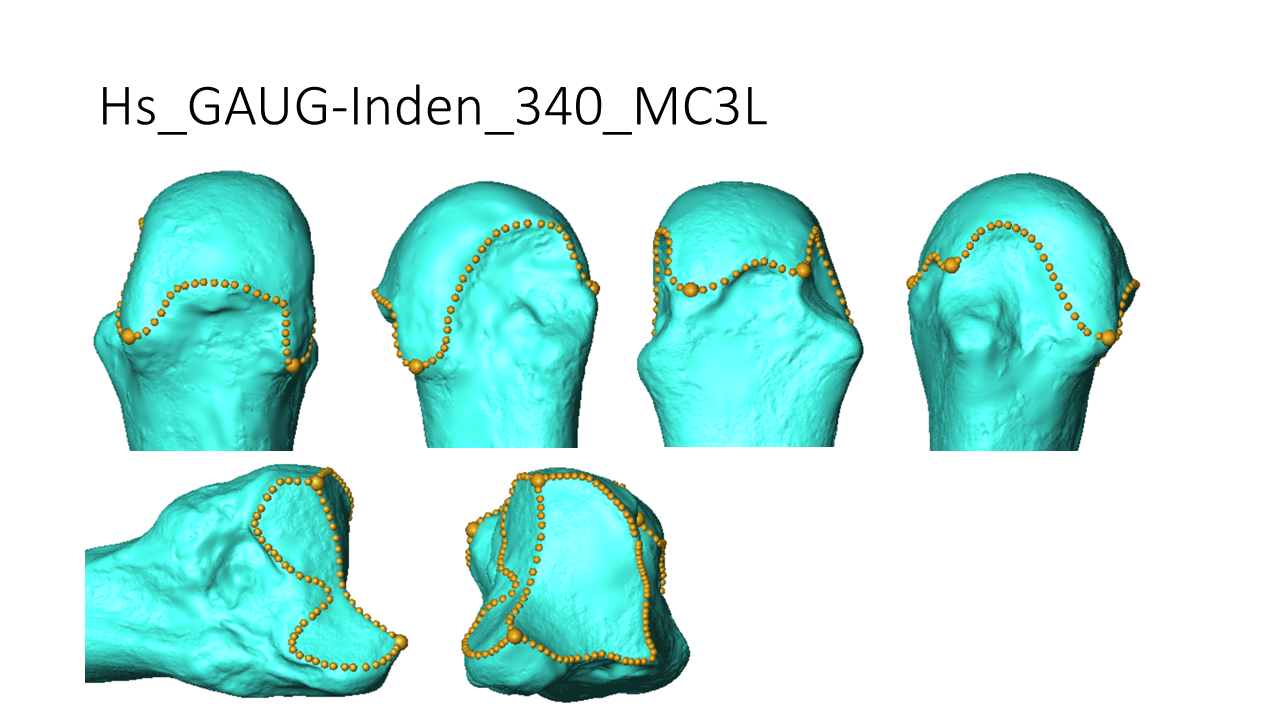

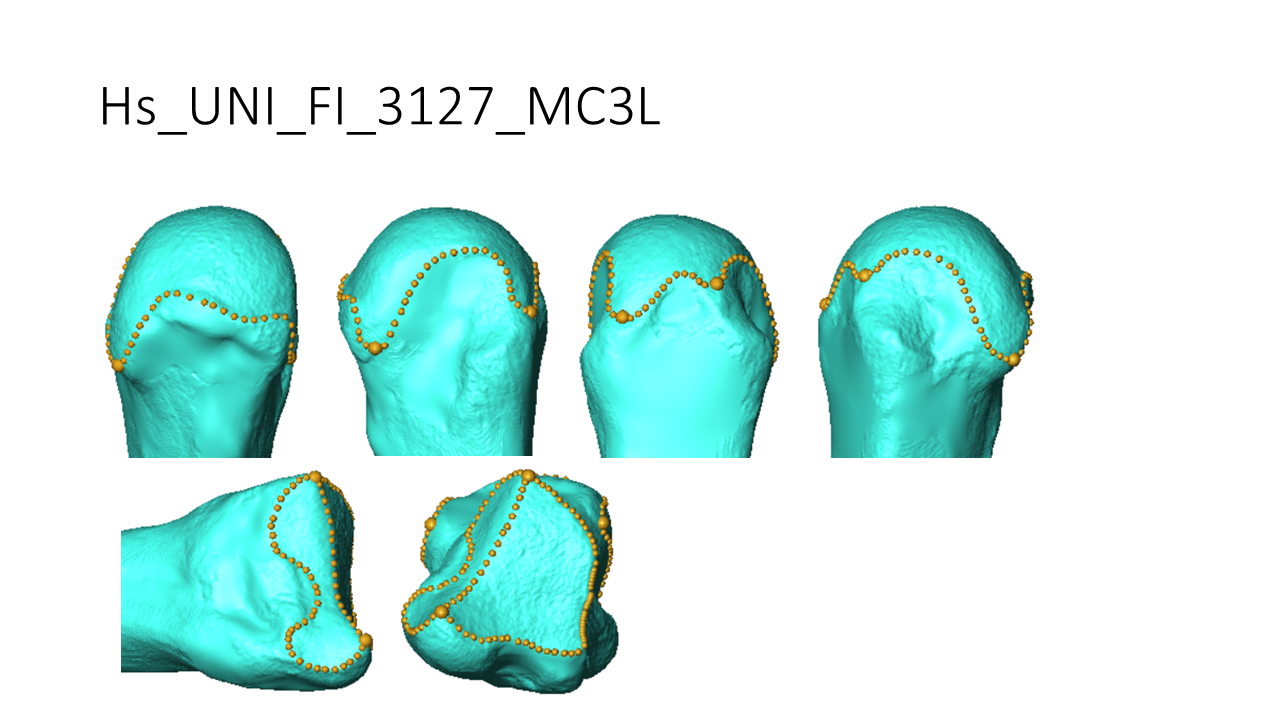


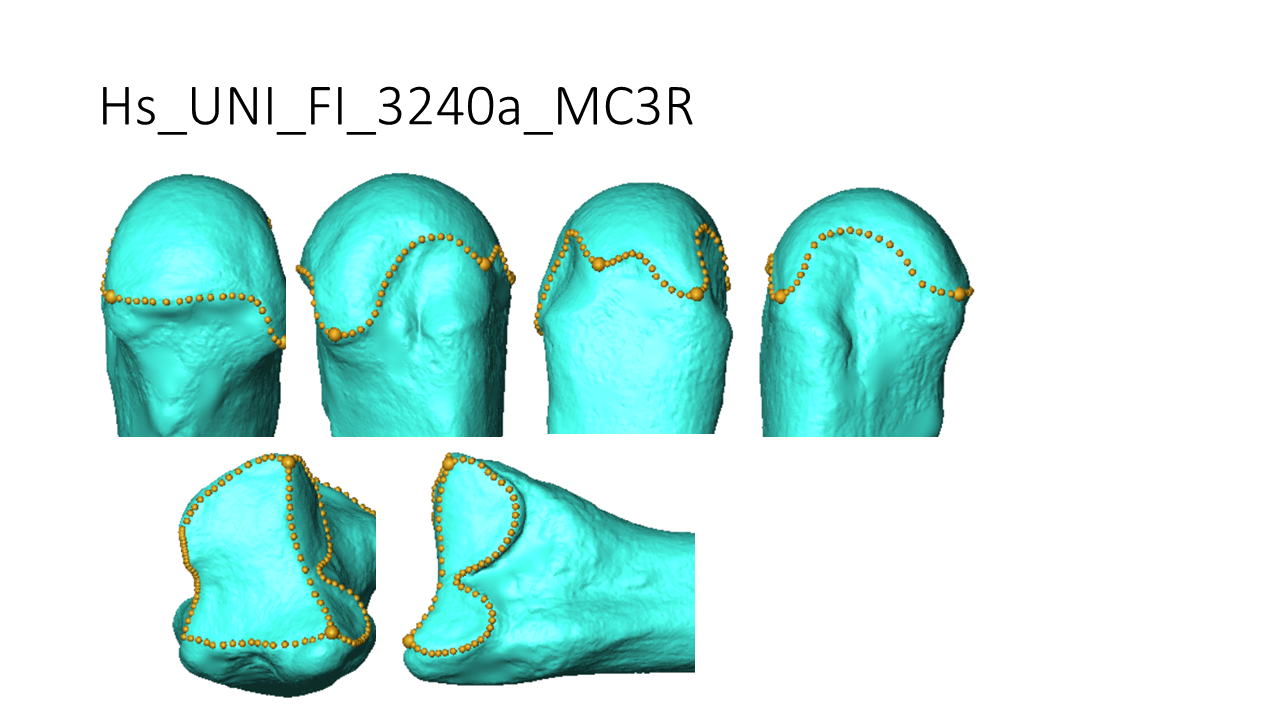


11.4. Atlas of all landmarked fourth metacarpals (n=23)
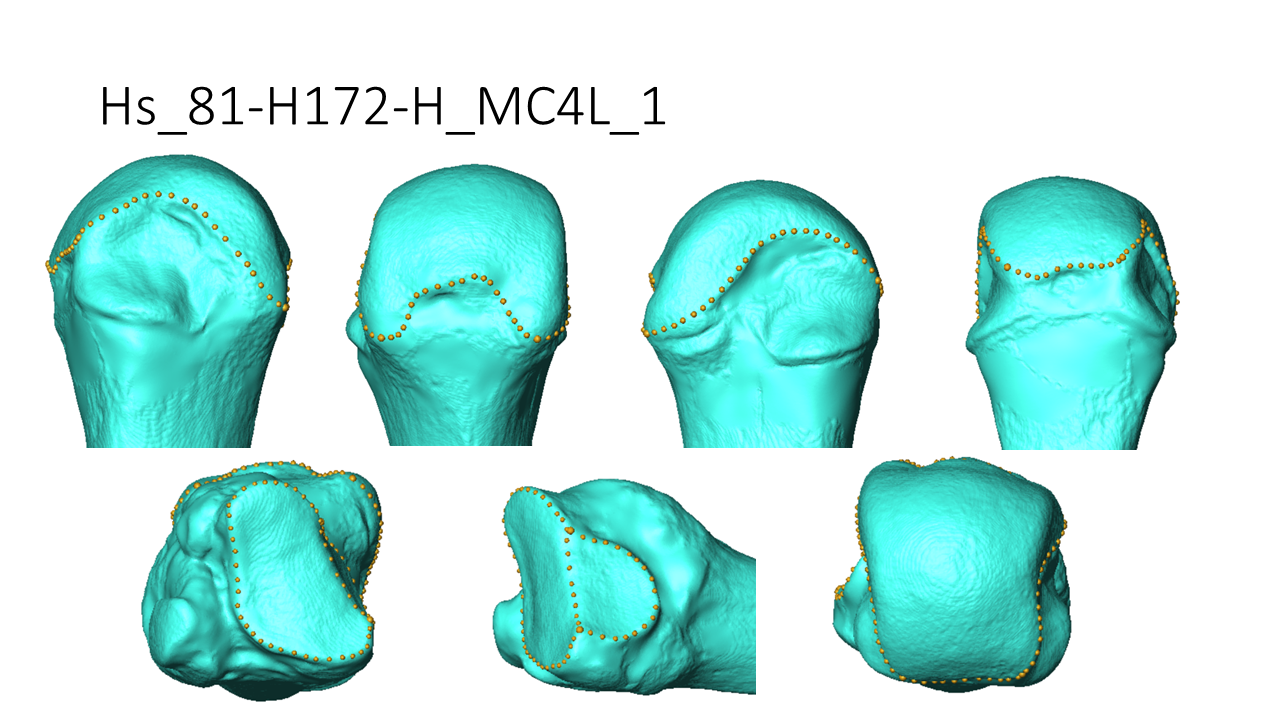

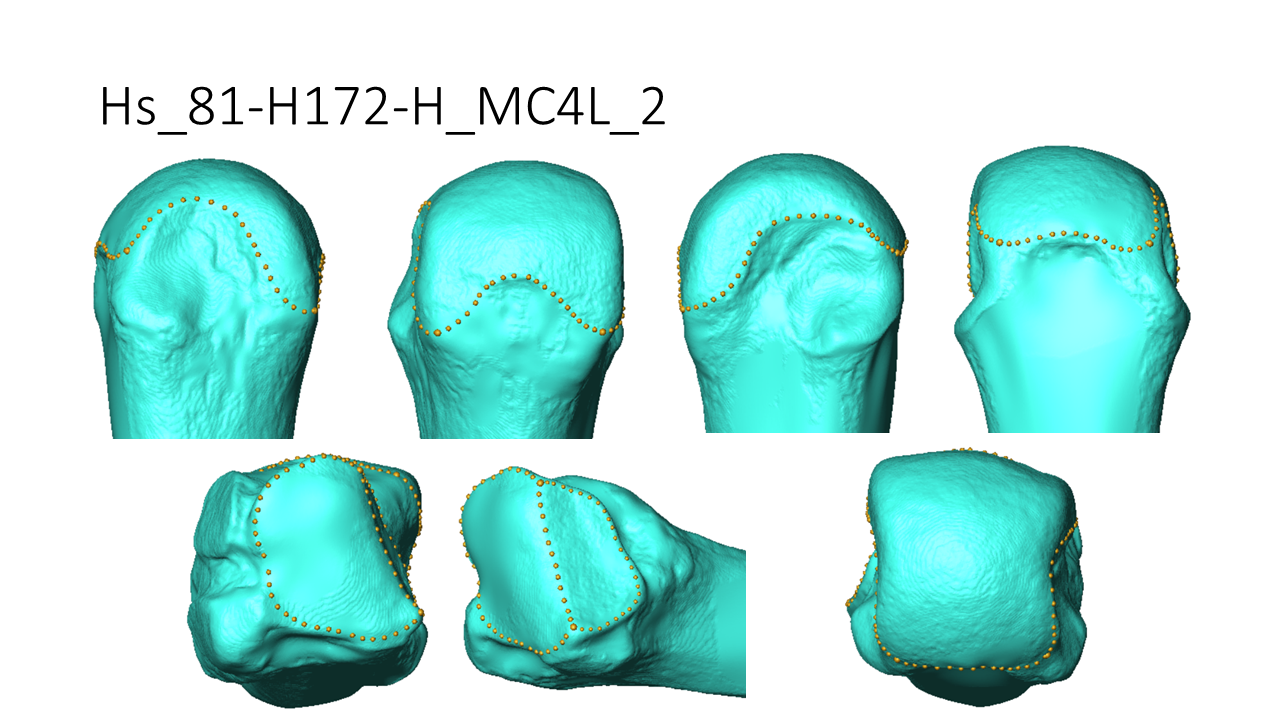

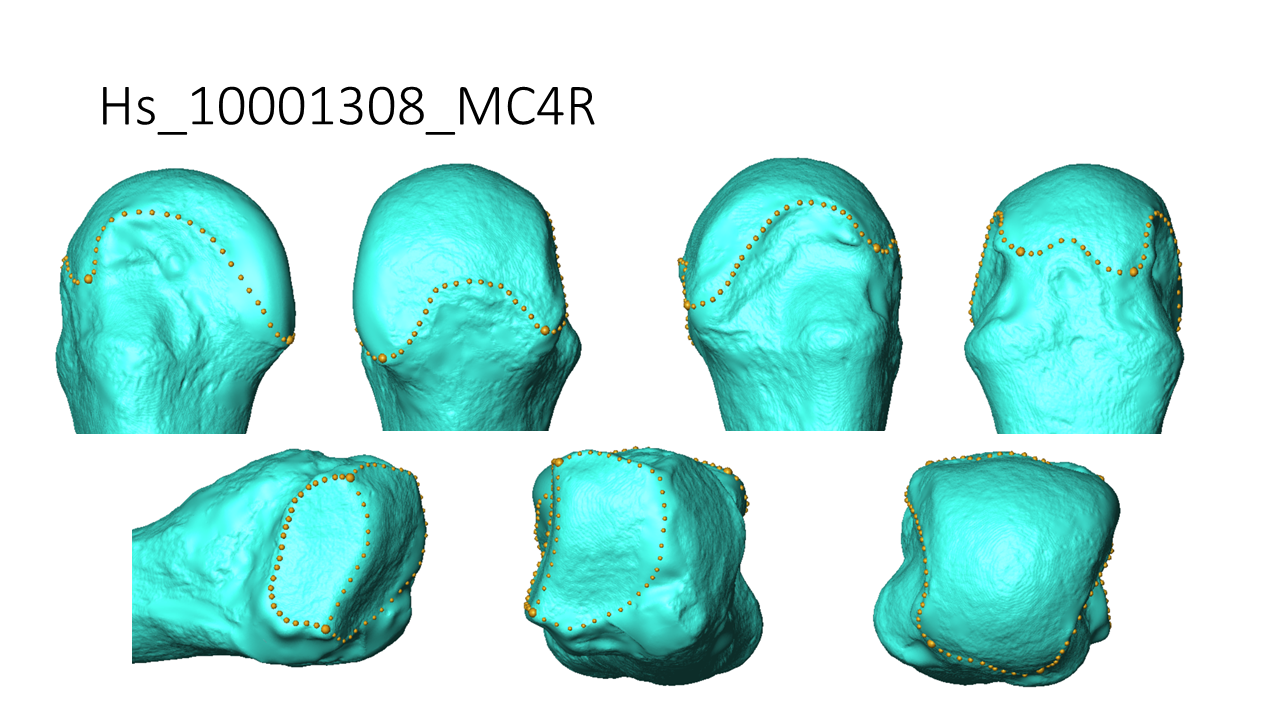

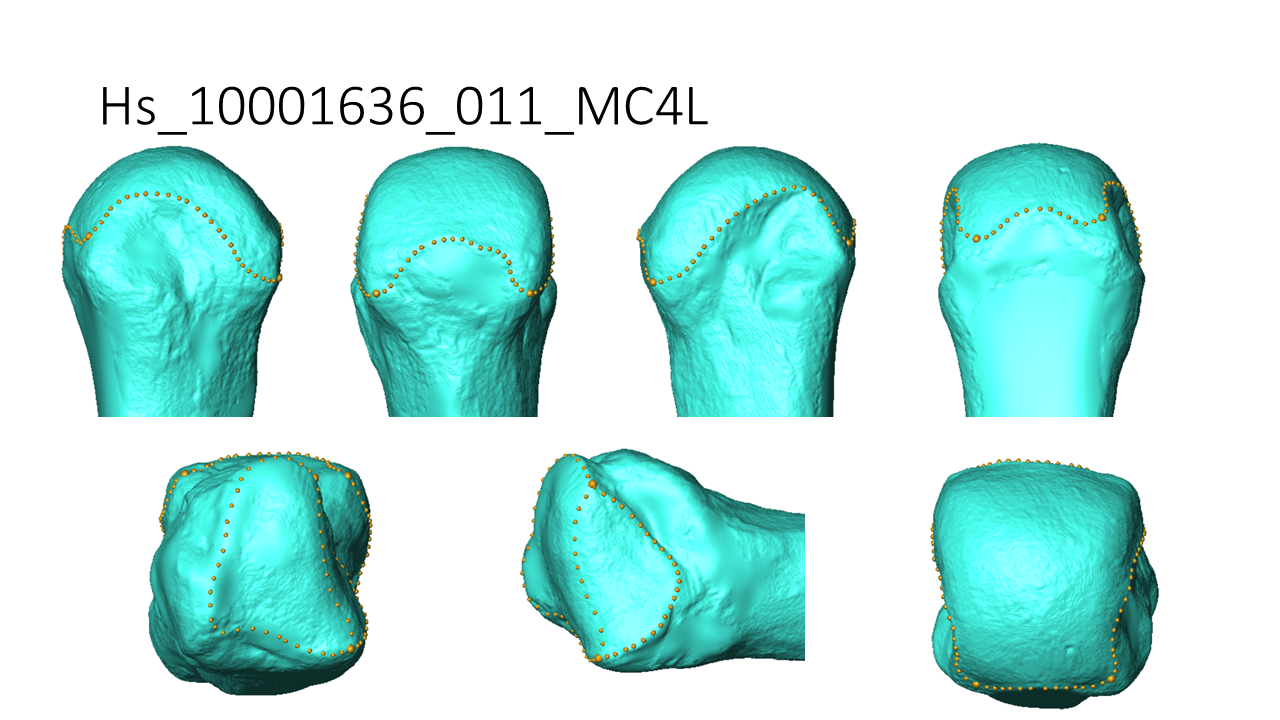


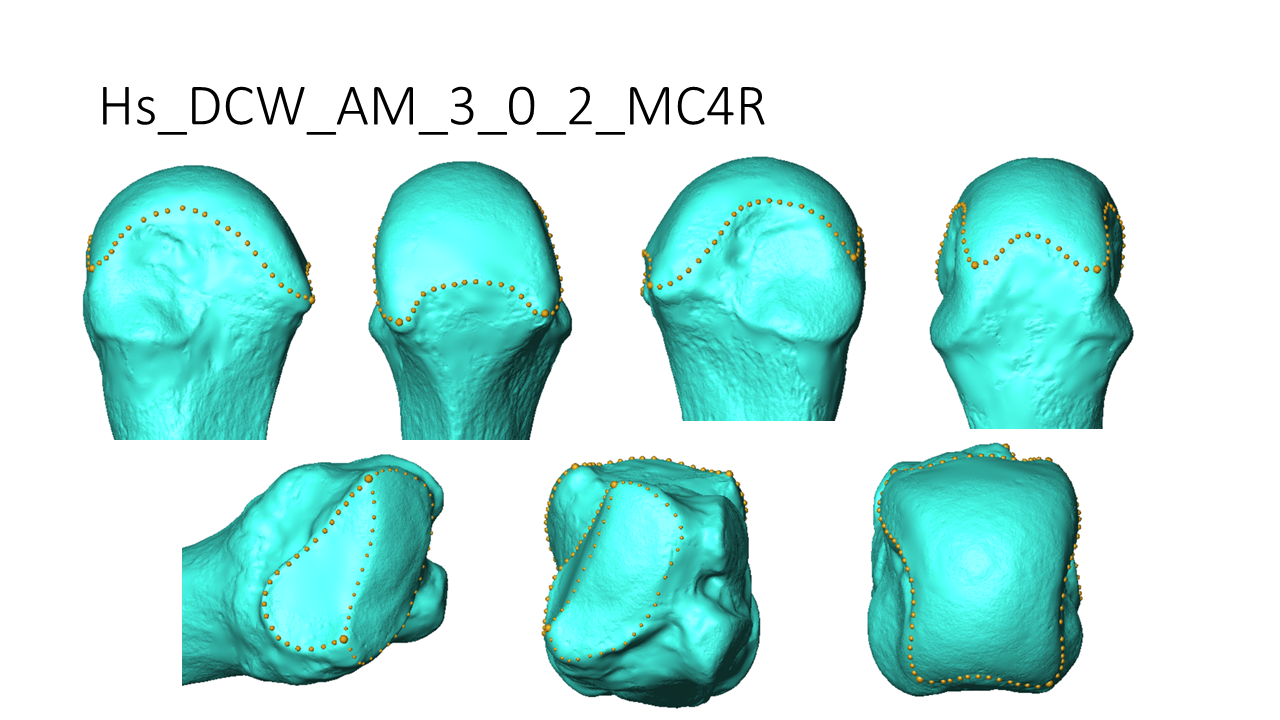

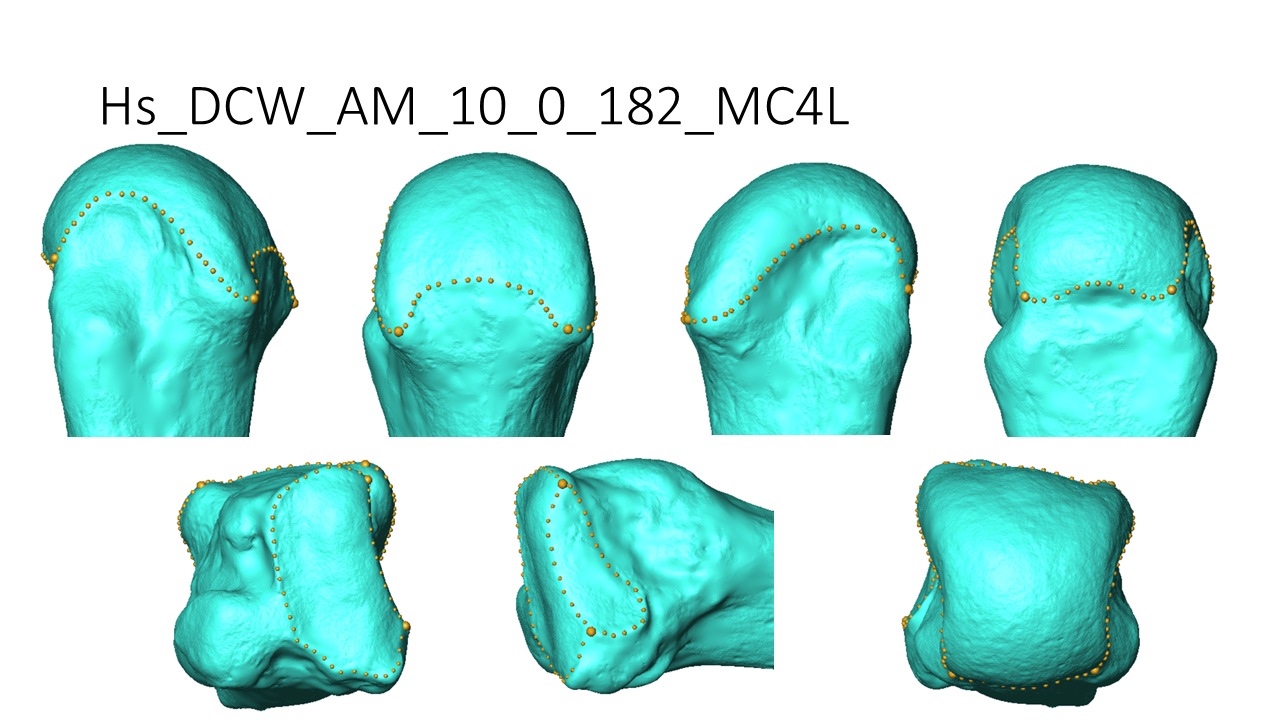

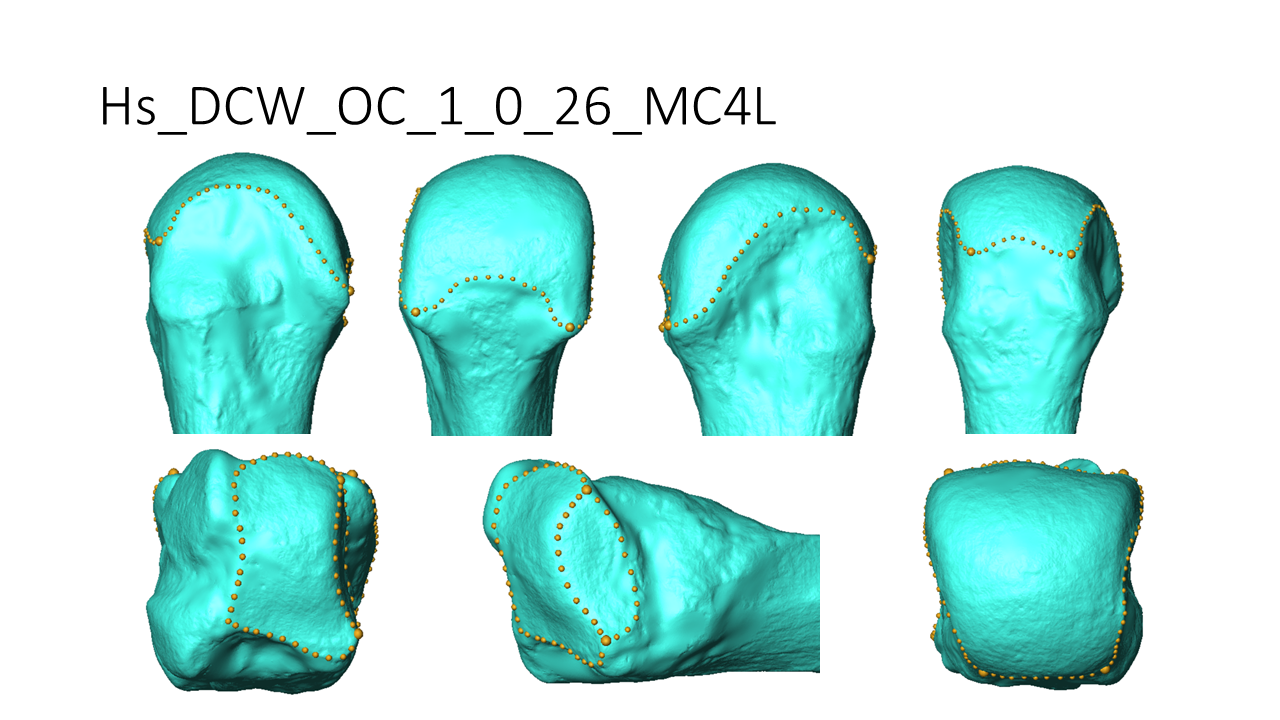

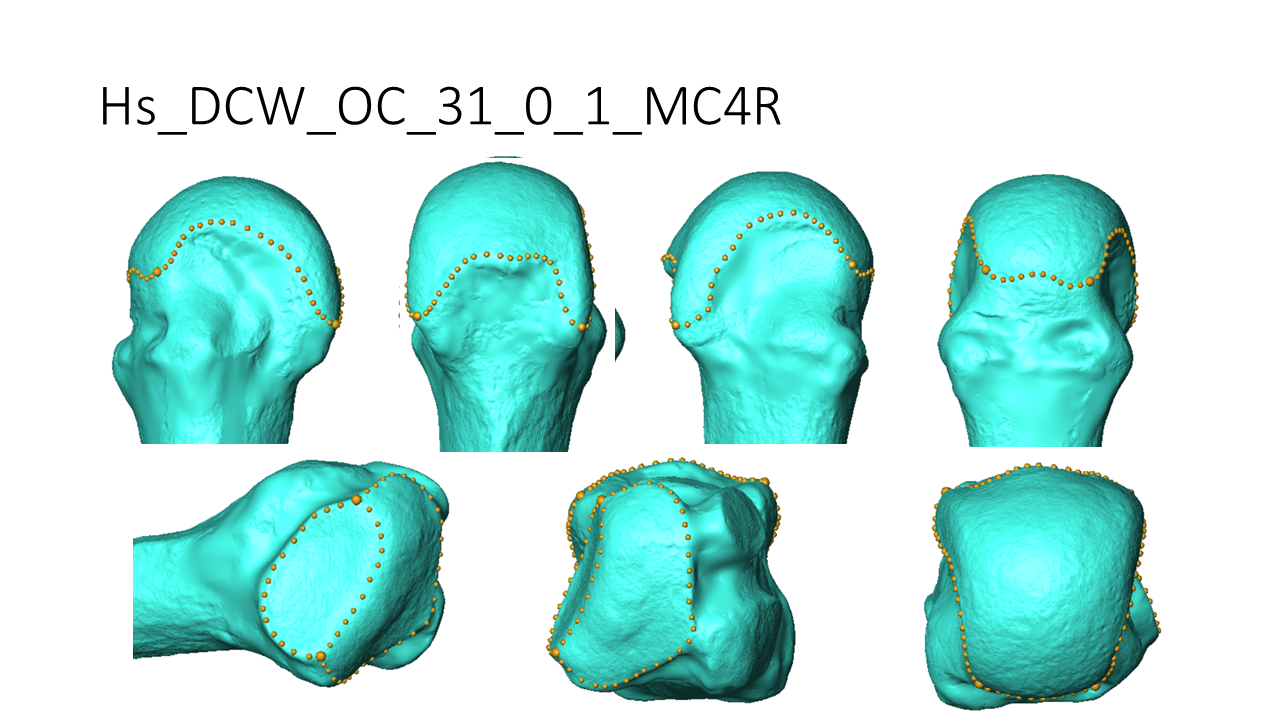


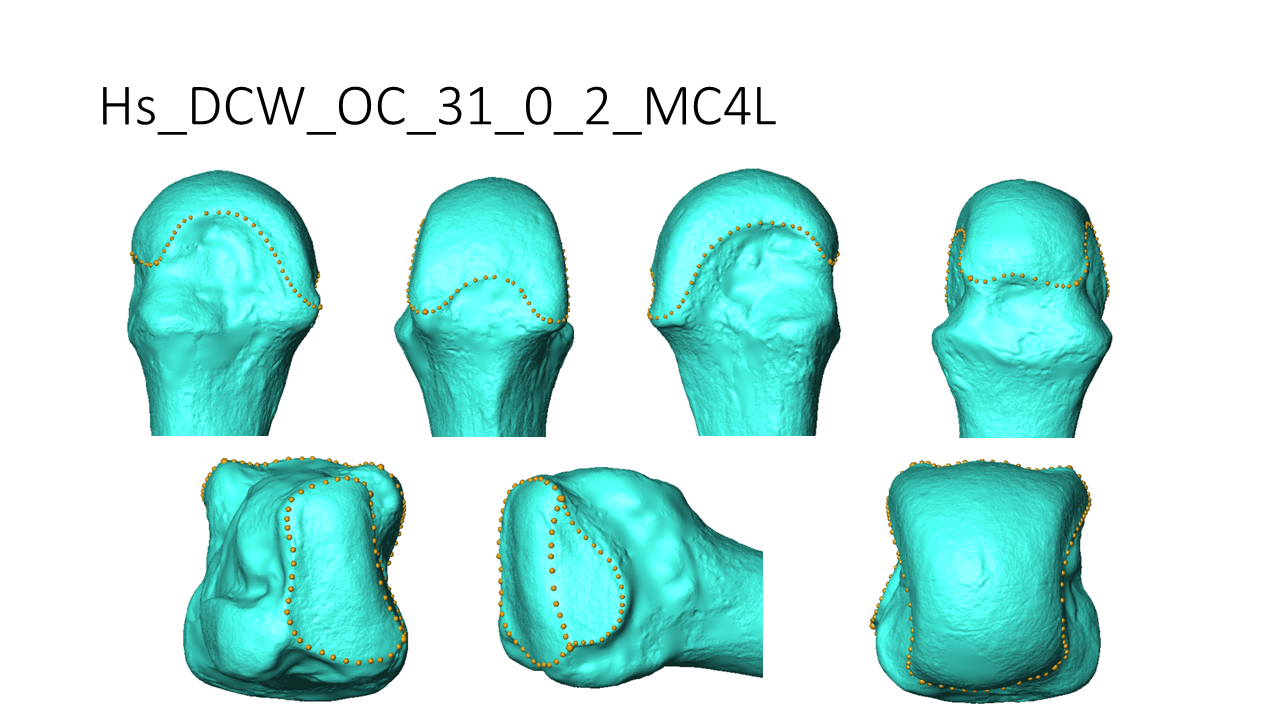

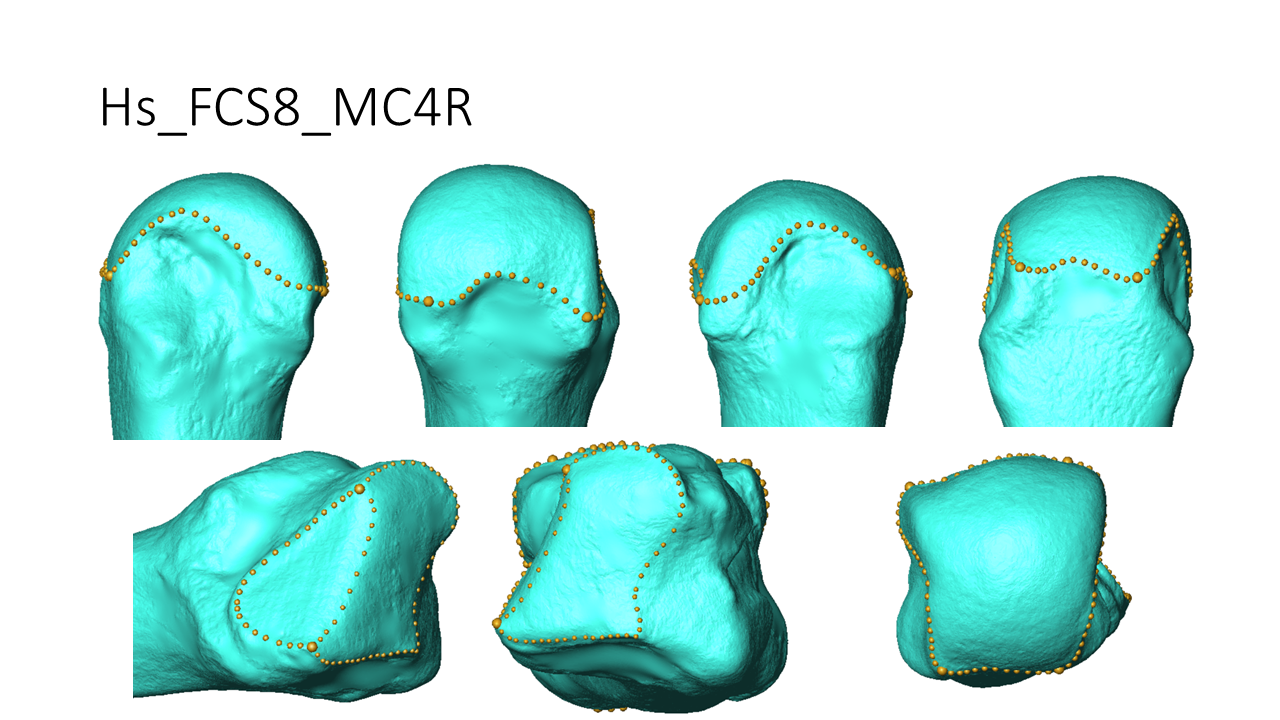


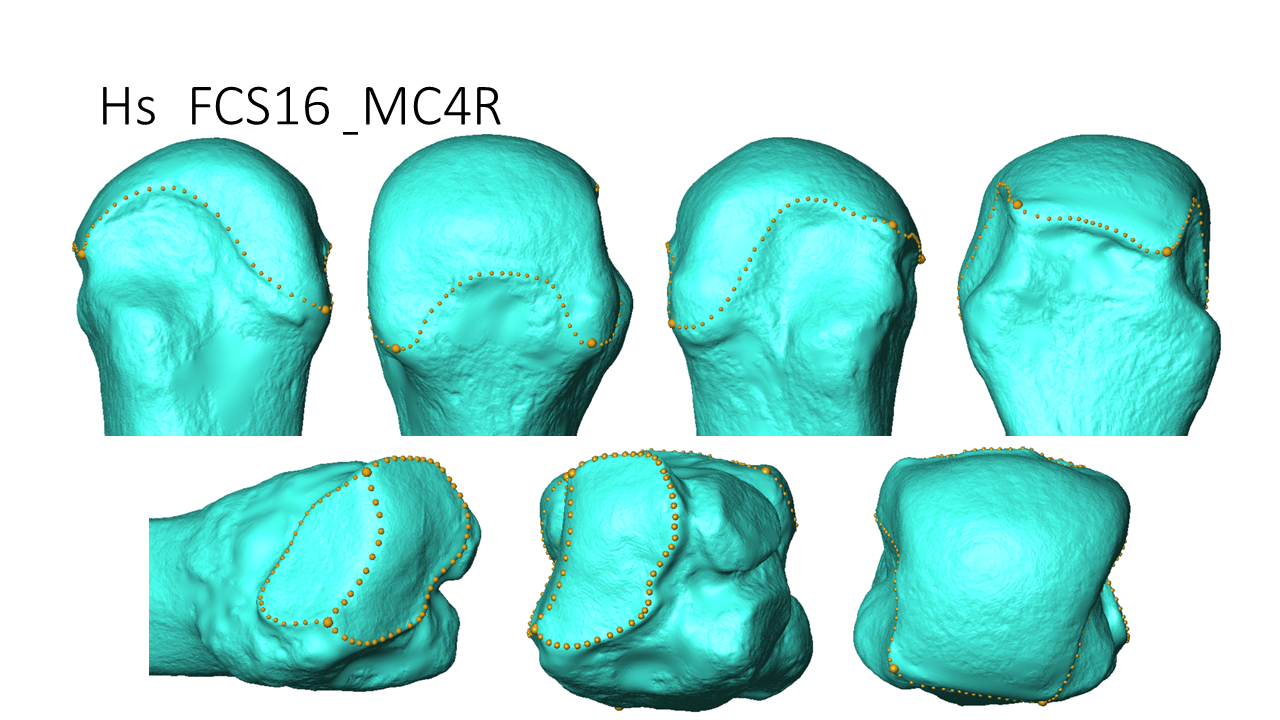


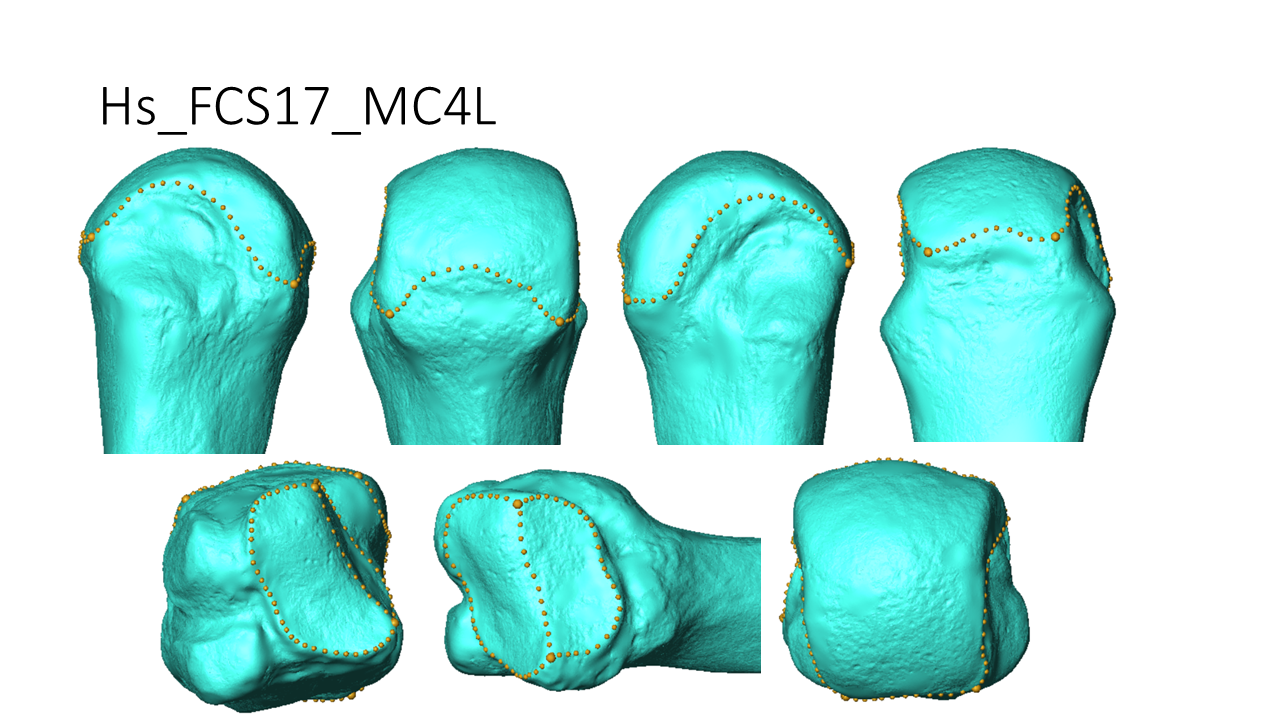

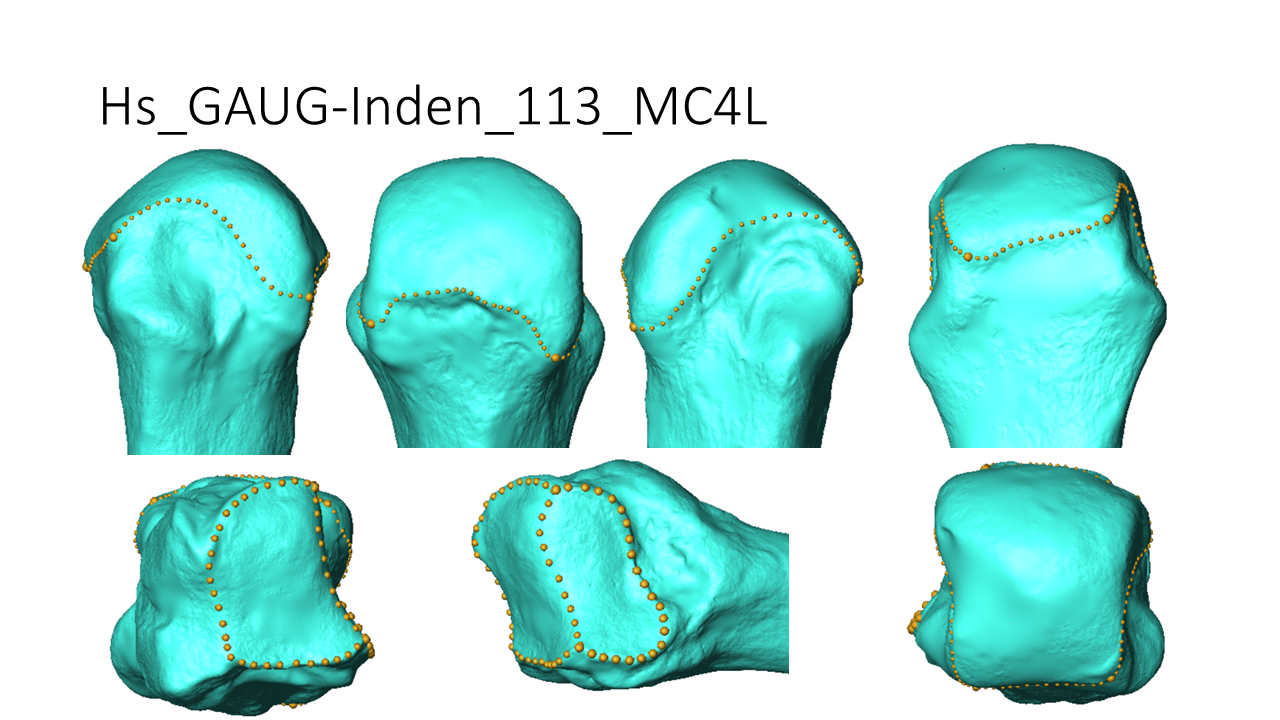

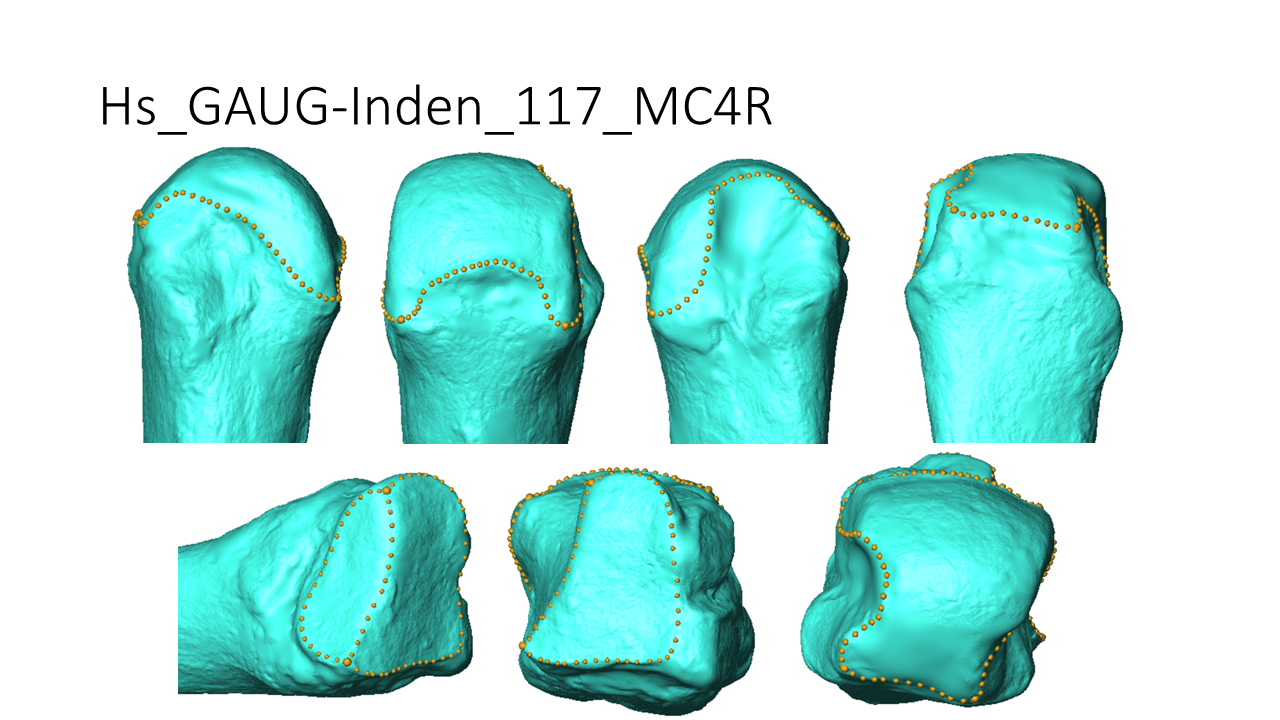

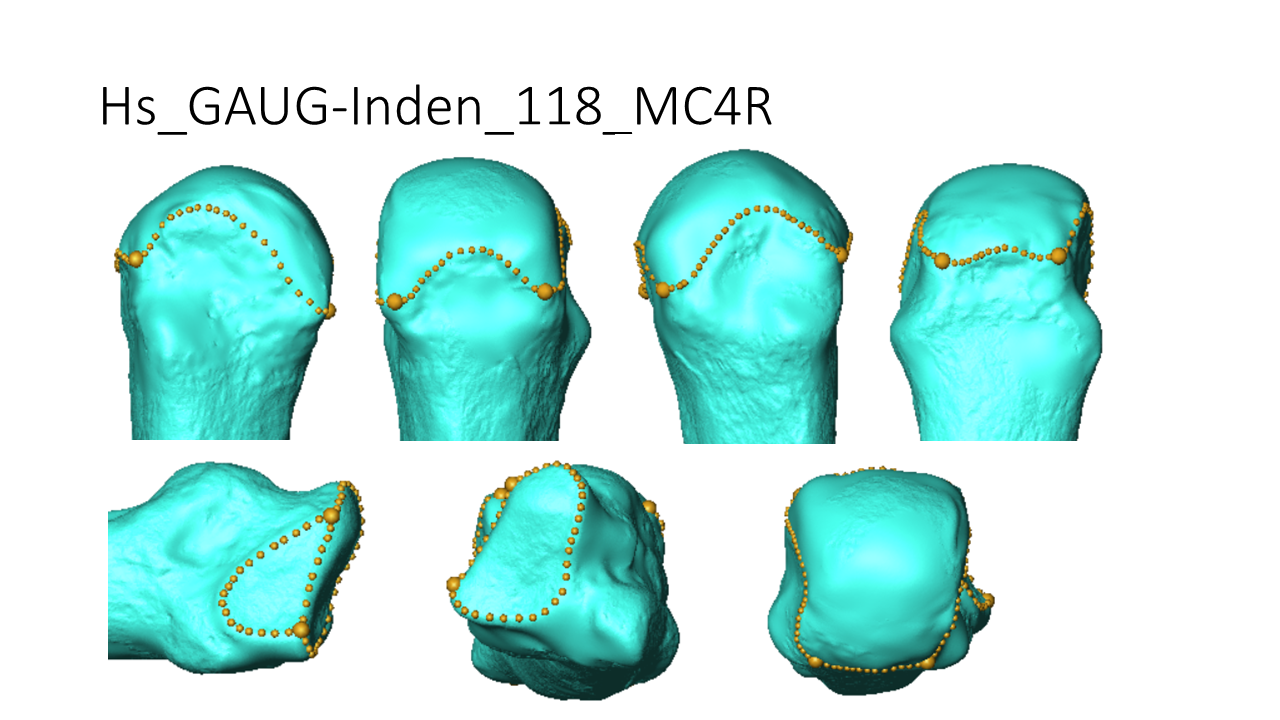


11.5. Atlas of all landmarked fifth metacarpals (n=22)
